# Supplementary material for: Radiomic tractometry reveals tract-specific imaging biomarkers in white matter
Source: Nat Commun. 2024 Jan 5;15:303. doi: 10.1038/s41467-023-44591-3 (PMC10770385; doi:10.1038/s41467-023-44591-3)
Supplement: Supplementary file 1 — Supplementary Information [file 41467_2023_44591_MOESM1_ESM.pdf]

Supplementary Material: Radiomic tractometry reveals tract-specific imaging biomarkers in white matter

Supplementary Note 1 – Number of parcels per tract

Supplementary Table 1. Number of parcels. To ensure consistent values across subjects a fixed n per dataset was used, calculated as the average of ten randomly chosen subjects. For consistency across tracts present in multiple datasets, n was only calculated on one dataset and propagated to the others.

Tract	Num Parcels n
CC_1	12
CC_2	15
CC_3	15
CC_4	17
CC_5	15
CC_6	18
CST_left	17
CST_right	17
T_PREM_left	11
T_PREM_right	11
ST_FO_left	7
ST_FO_right	7
ST_PREM_left	8
ST_PREM_right	8
ICP_left	9
ICP_right	9
SCP_left	12
SCP_right	12
T_PREF_left	13
T_PREF_right	13
T_PAR_left	12
T_PAR_right	12
ST_PAR_left	13
ST_PAR_right	12

Supplementary Note 2 – Automatic diagnosis results

SCHZ

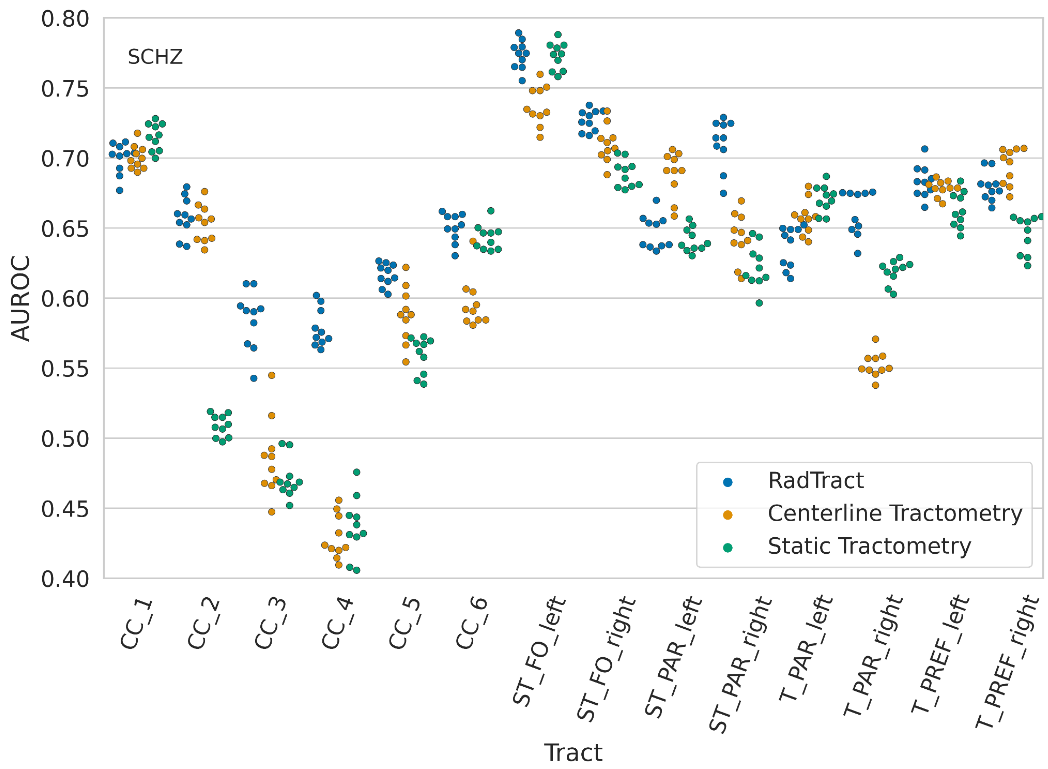

Supplementary Figure 1. Classification results for the SCHZ dataset. The individual points correspond to the repetitions of the experiment.

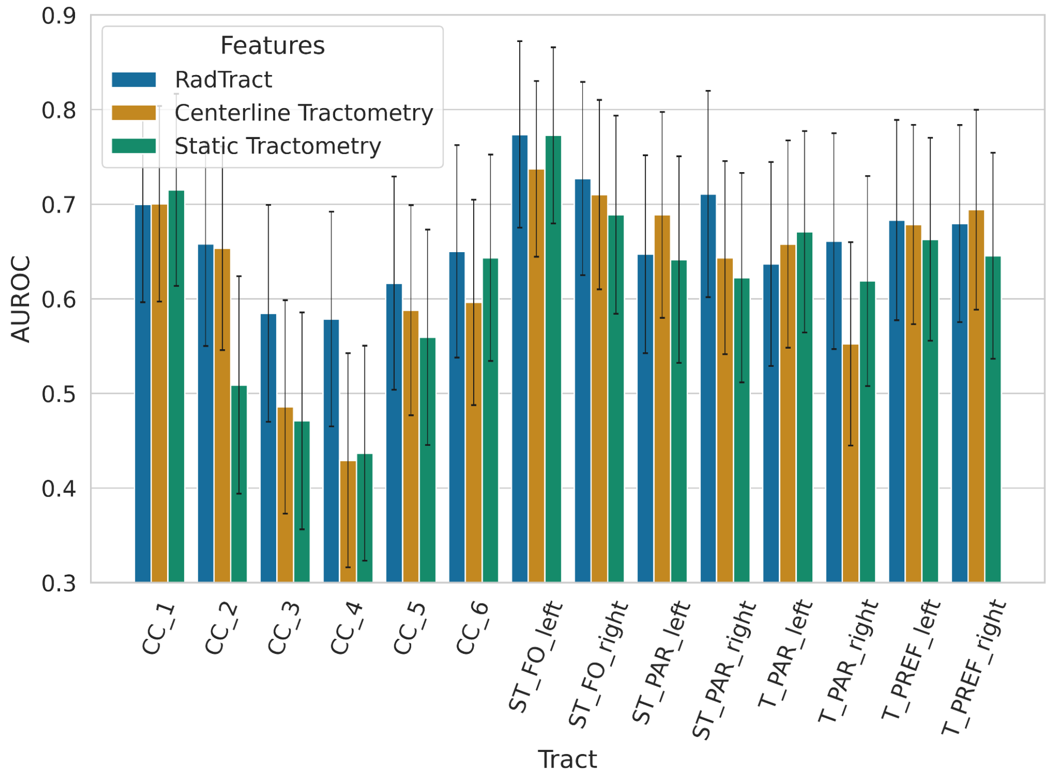

Supplementary Figure 2. Mean classification results for the SCHZ dataset with 0.95 confidence intervals of the ROC curves averaged over repetitions.

Supplementary Table 2. p-Values of statistically significant differences between first and second ranking method per tract. Tests for significance of the classification experiments were performed using Delong's method for statistical comparisons of ROC curves without correction for multiple comparisons. Details about the statistical analysis can be found in the methods section.

Tract	Rank 1 Method	Rank 2 Method	p-Value
CC_4	RadTract	Static Tractometry	4.90e-02

CAT

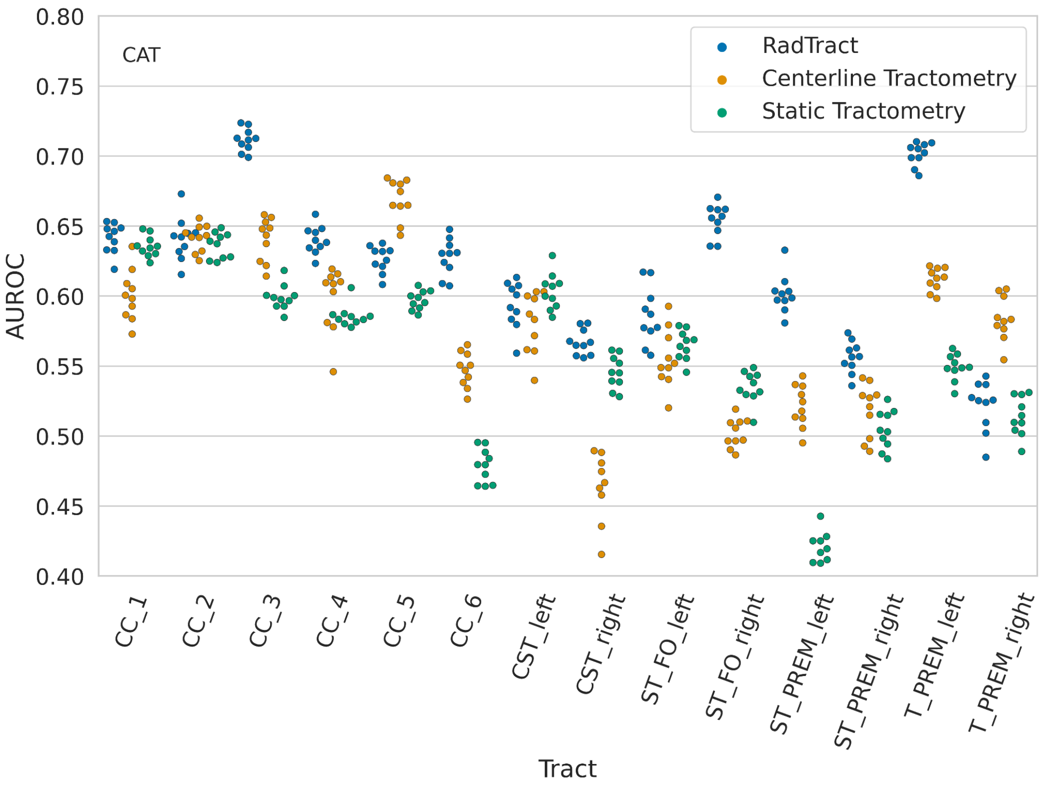

Supplementary Figure 3. Classification results for the CAT dataset. The individual points correspond to the repetitions of the experiment.

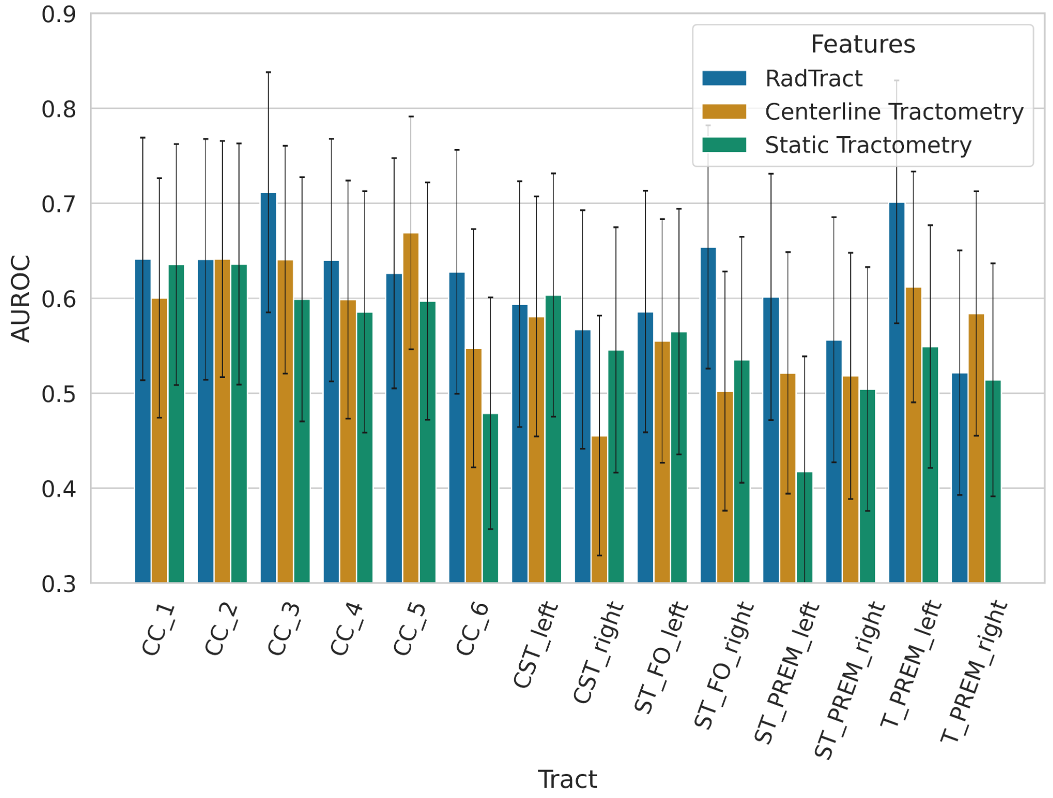

Supplementary Figure 4. Mean classification results for the CAT dataset with 0.95 confidence intervals of the ROC curves averaged over repetitions.

Supplementary Table 3. p-Values of statistically significant differences between first and second ranking method per tract. Tests for significance of the classification experiments were performed using Delong's method for statistical comparisons of ROC curves without correction for multiple comparisons. Details about the statistical analysis can be found in the methods section.

Tract	Rank 1 Method	Rank 2 Method	p-Value
CC_3	RadTract	Centerline Tractometry	1.78e-02
CST_left	Static Tractometry	RadTract	2.02e-02
ST_FO_right	RadTract	Static Tractometry	2.09e-02
ST_PREM_left	RadTract	Centerline Tractometry	9.91e-03
T_PREM_left	RadTract	Centerline Tractometry	4.4e-02

ADNI

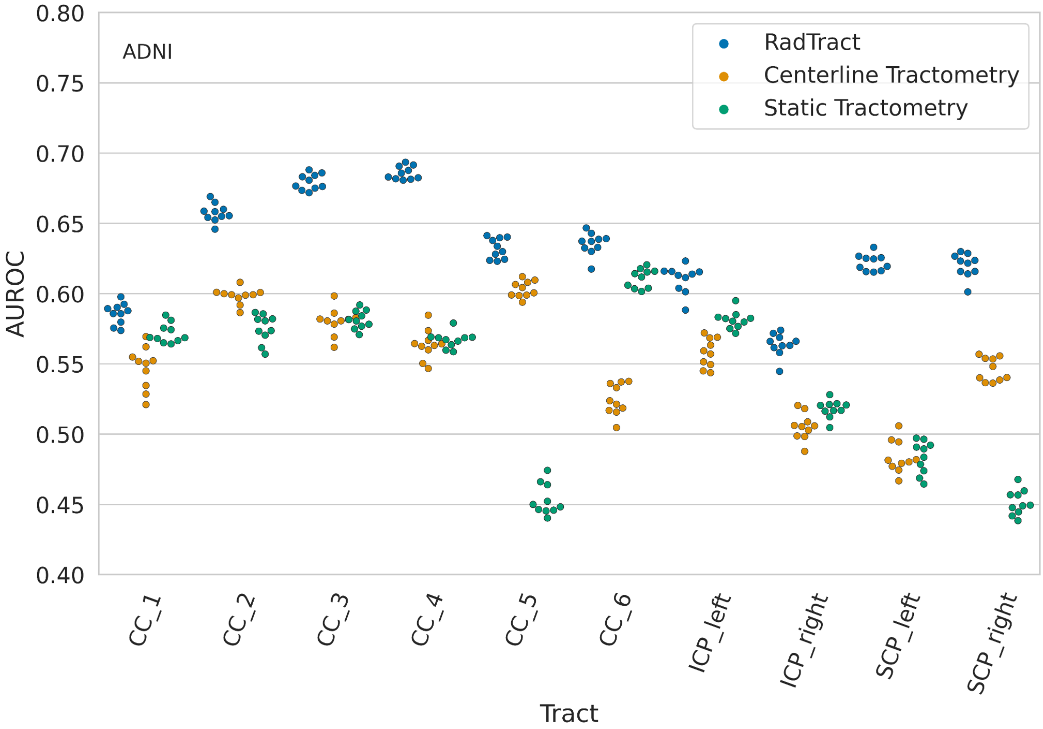

Supplementary Figure 5. Classification results for the ADNI dataset. The individual points correspond to the repetitions of the experiment.

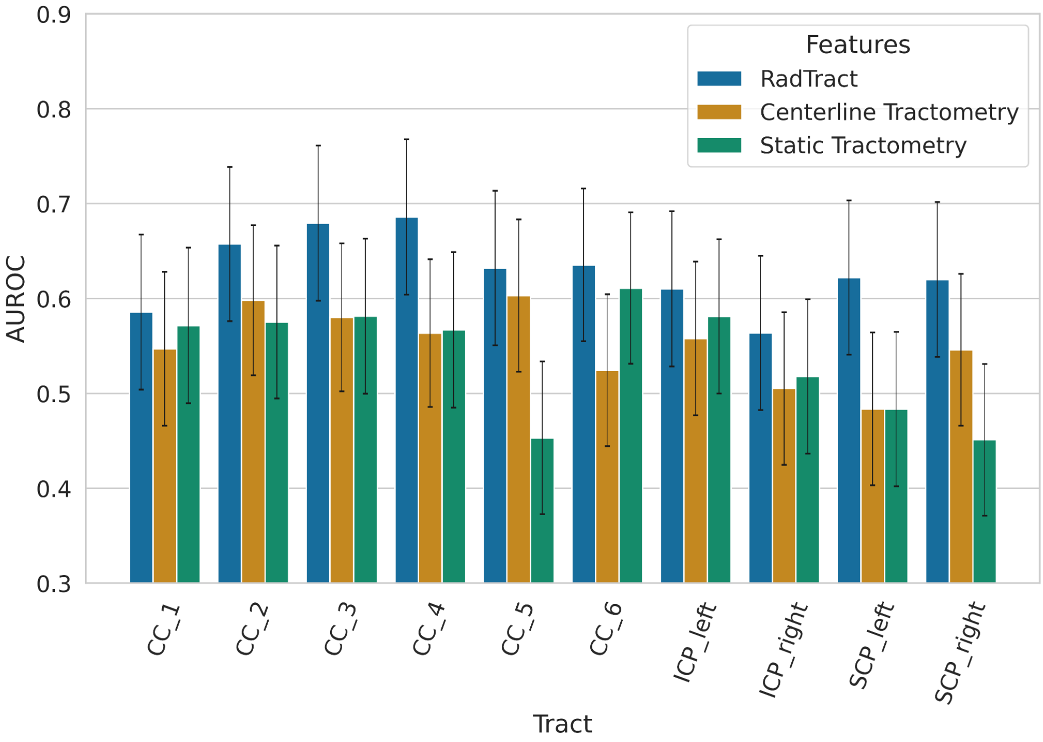

Supplementary Figure 6. Mean classification results for the ADNI dataset with 0.95 confidence intervals of the ROC curves averaged over repetitions.

Supplementary Table 4. p-Values of statistically significant differences between first and second ranking method per tract. Multiple p-values per cell correspond to different classes of the multi-class experiment. Tests for significance of the classification experiments were performed using Delong's method for statistical comparisons of ROC curves without correction for multiple comparisons. Details about the statistical analysis can be found in the methods section.

Tract	Rank 1 Method	Rank 2 Method	p-Value
CC_2	RadTract	Centerline Tractometry	3.21e-02
CC_3	RadTract	Static Tractometry	8.33e-03 / 6.68e-05
CC_4	RadTract	Static Tractometry	6.56e-03 / 8.84e-03 / 1.85e-03
ICP_right	RadTract	Static Tractometry	5.12e-03
SCP_left	RadTract	Centerline Tractometry	2.03e-03 / 9.14e-04
SCP_right	RadTract	Centerline Tractometry	2.16e-02

PPMI

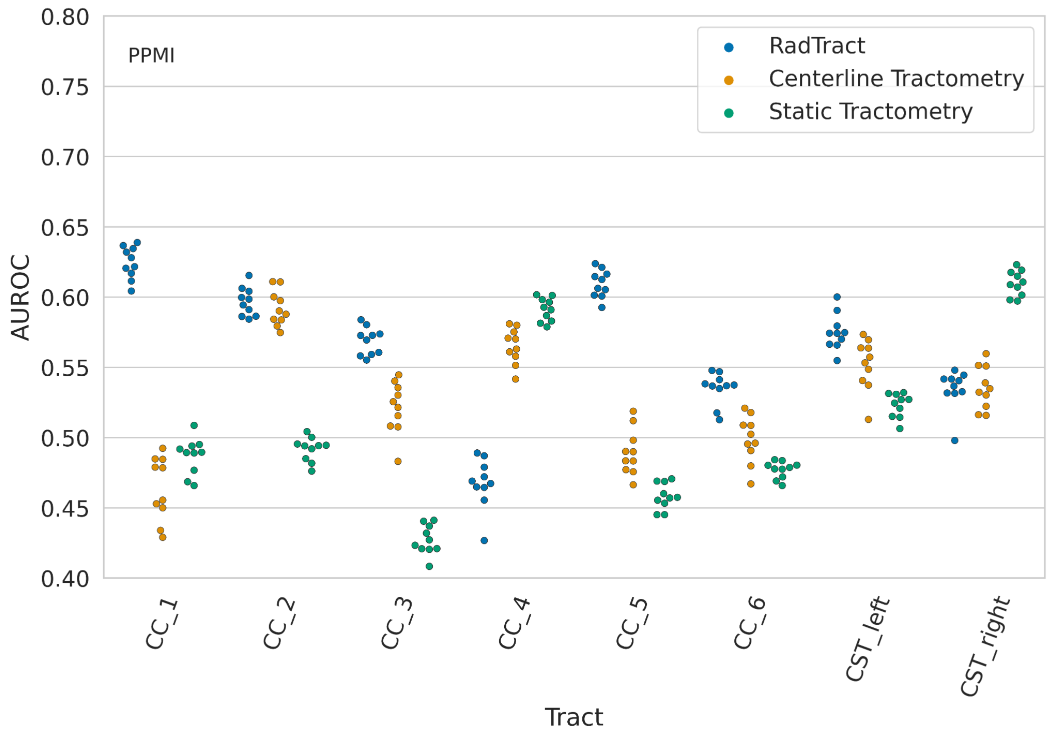

Supplementary Figure 7. Classification results for the PPMI dataset. The individual points correspond to the repetitions of the experiment.

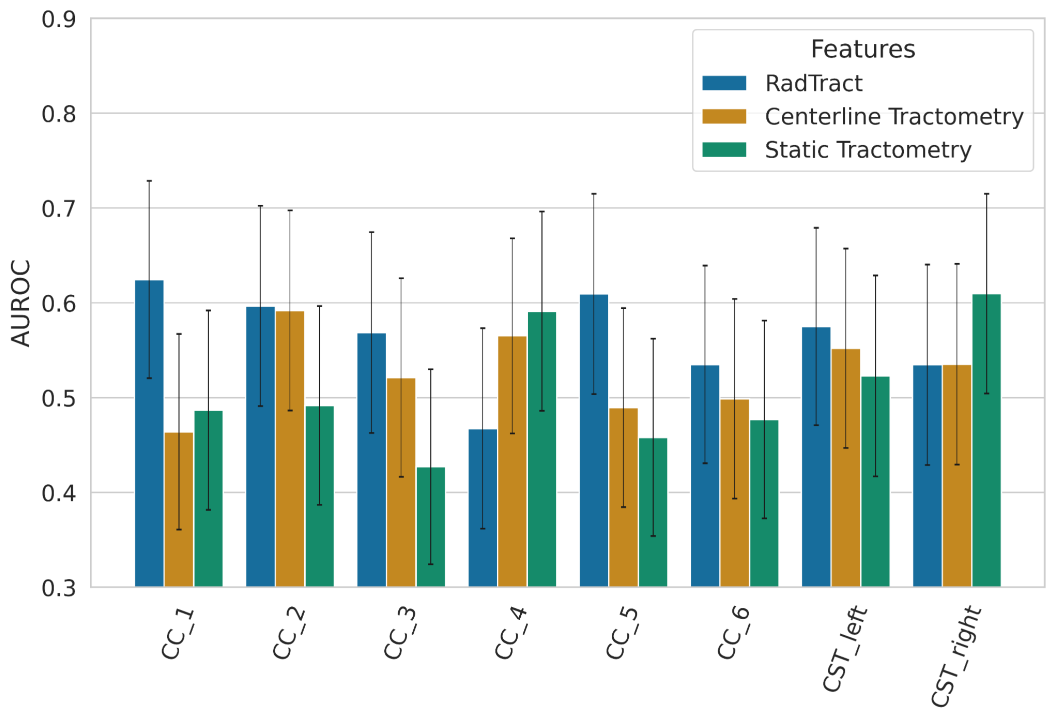

Supplementary Figure 8. Mean classification results for the PPMI dataset with 0.95 confidence intervals of the ROC curves averaged over repetitions.

Supplementary Table 5. p-Values of statistically significant differences between first and second ranking method per tract. Multiple p-values per cell correspond to different classes of the multi-class experiment. Tests for significance of the classification experiments were performed using Delong's method for statistical comparisons of ROC curves without correction for multiple comparisons. Details about the statistical analysis can be found in the methods section.

Tract	Rank 1 Method	Rank 2 Method	p-Value
CC_1	RadTract	Static Tractometry	8.97e-03 / 4.44e-02
CC_3	RadTract	Centerline Tractometry	4.27e-02
CC_5	RadTract	Centerline Tractometry	5.93e-03
CST_right	Static Tractometry	Centerline Tractometry	3.77e-02

Supplementary Note 3 – Regression of demographic and clinical parameters results

Supplementary Table 6. Summary statistics of the CAT dataset regression targets.

Target	Minimum	Maximum	Mean	STDEV
Age	18	65	38.3	11.4
Pack-Years	0	45	10.66	11.33
Education	2	24	13.01	2.88
BPRS Total	18	80	37.46	12.58
PANSS Total	30	133	66.6	20.36
GAF	20	100	69.44	17.14
OLZe	0	173	19.26	18.06

Age

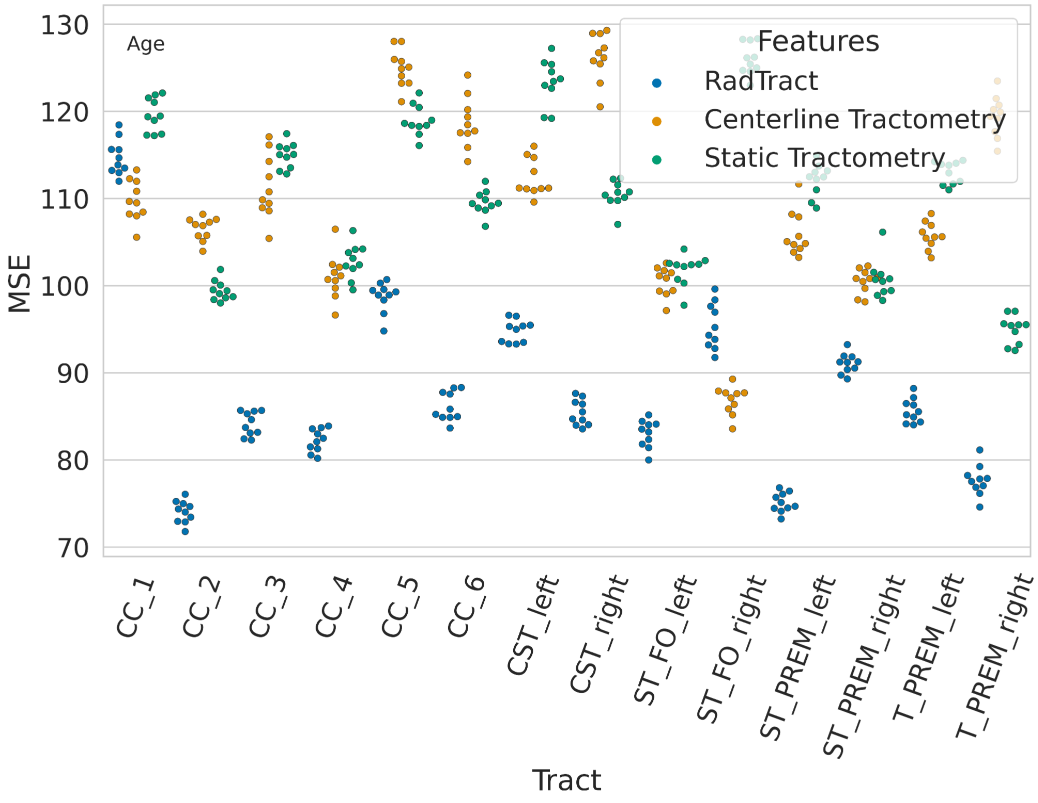

Supplementary Figure 9. Mean squared errors for the Age regression experiments on the CAT dataset.

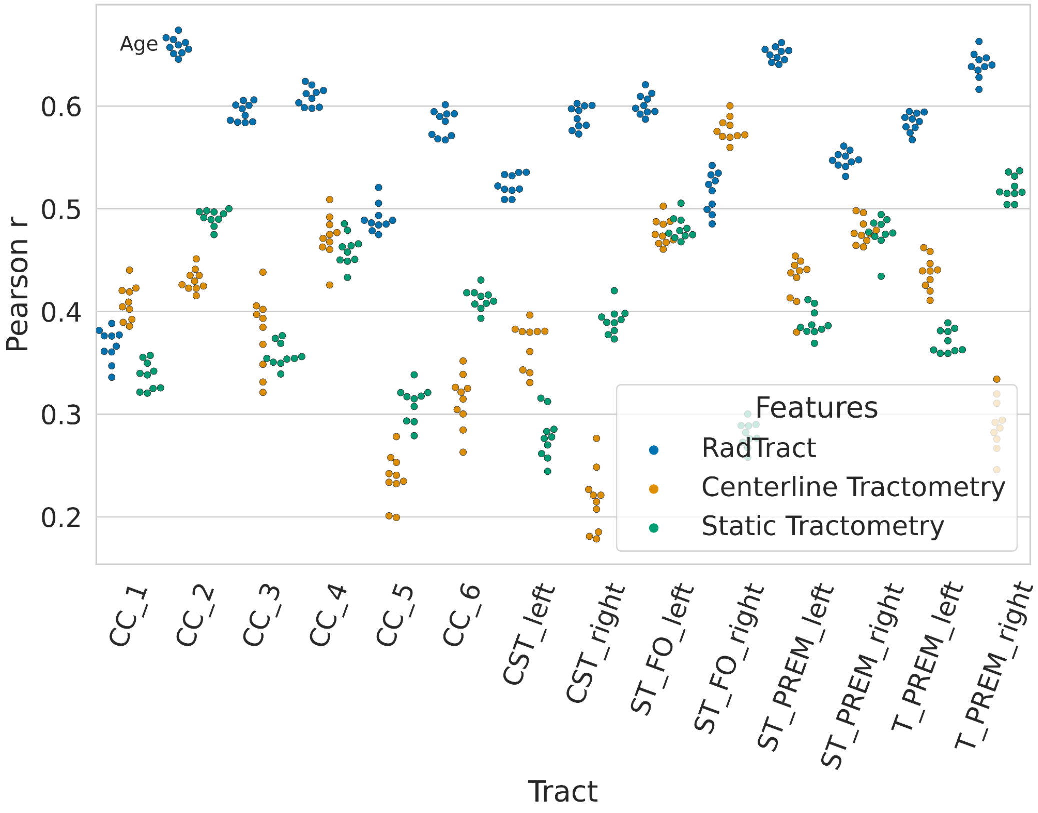

Supplementary Figure 10. Pearson’s correlations for the Age regression experiments on the CAT dataset.

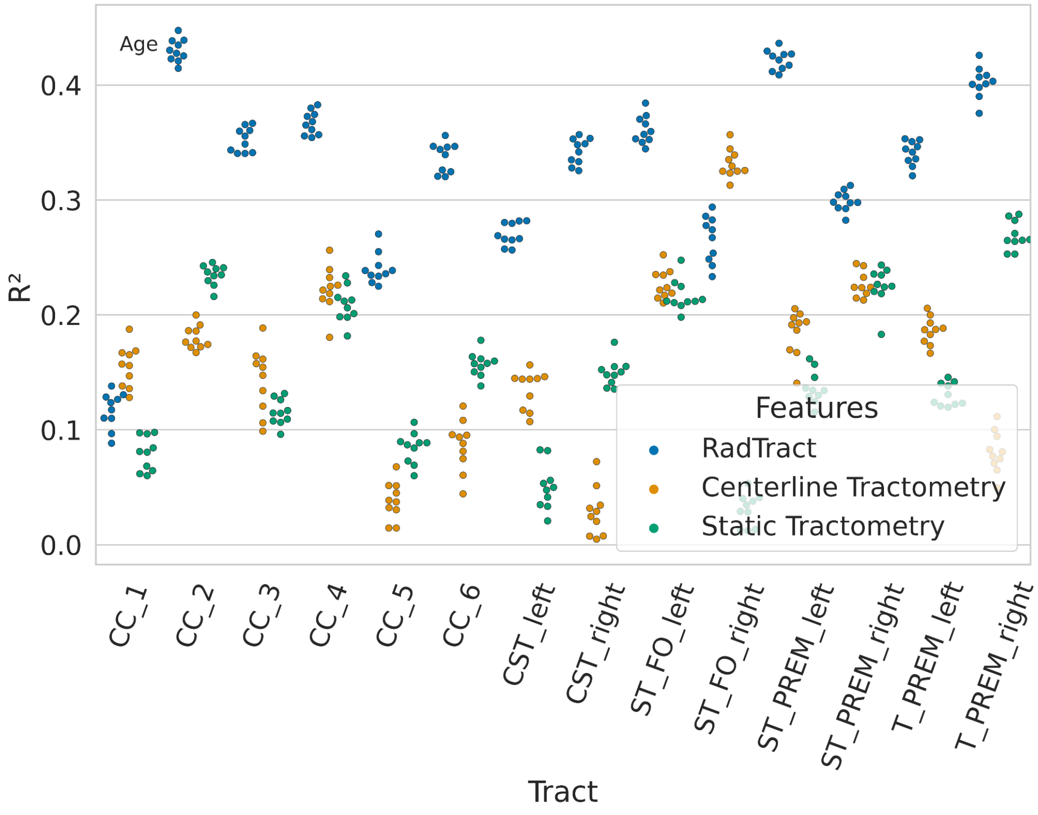

Supplementary Figure 11. Coefficients of determination for the Age regression experiments on the CAT dataset.

Pack-Years

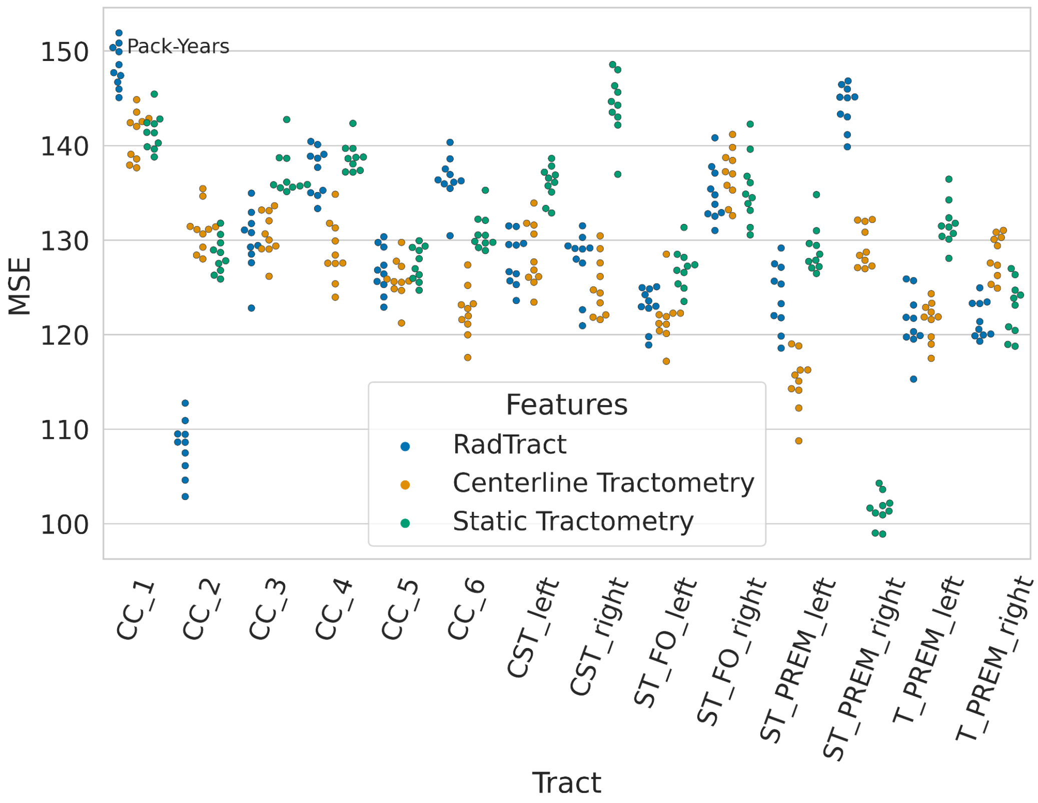

Supplementary Figure 12. Mean squared errors of the Pack-Years regression experiments on the CAT dataset.

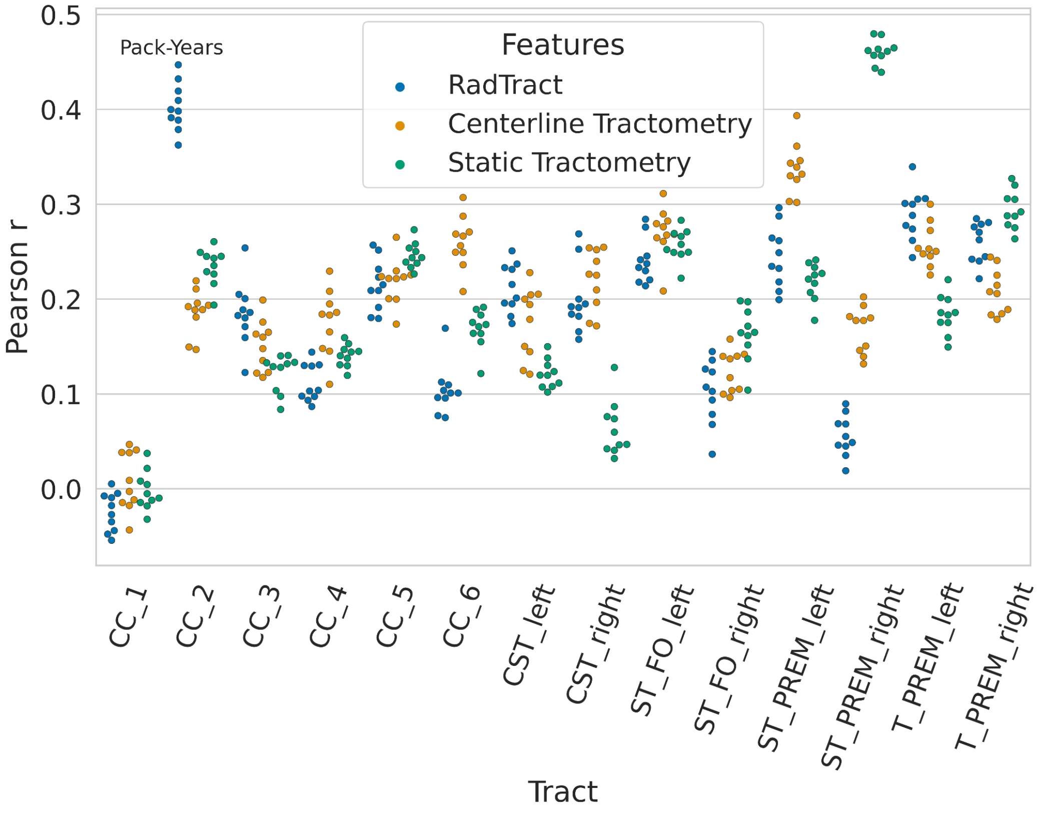

Supplementary Figure 13. Pearson's correlations for the Pack-Years regression experiments on the CAT dataset.

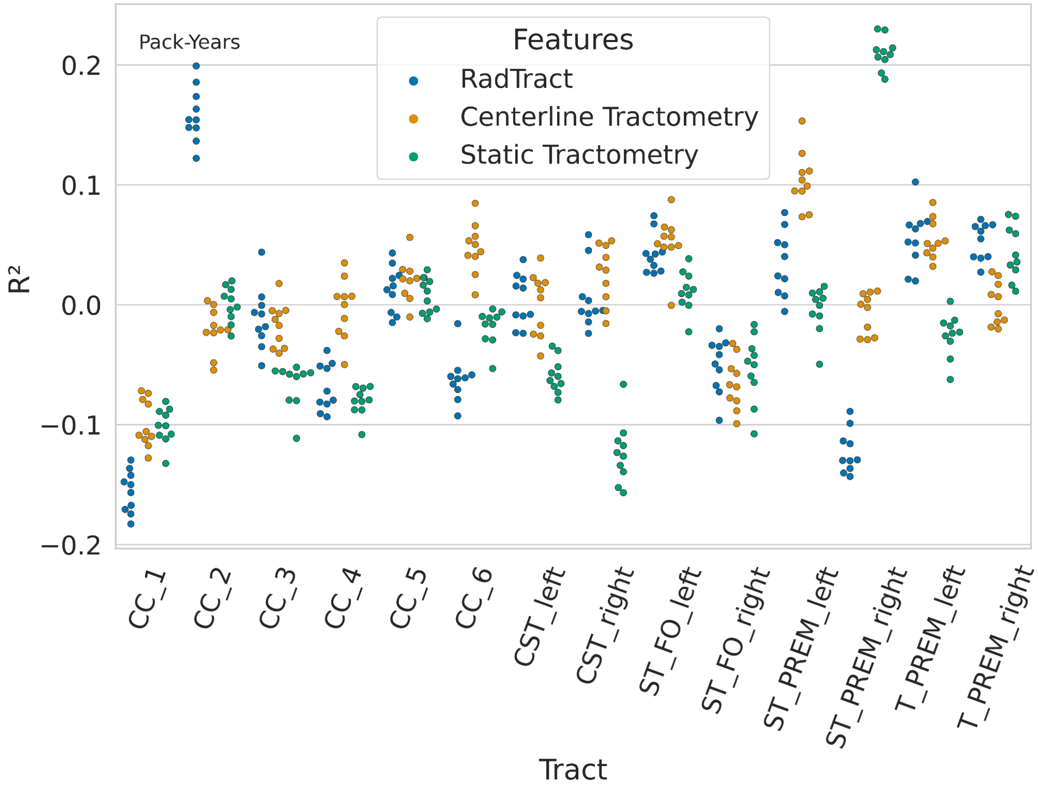

Supplementary Figure 14. Coefficients of determination for the Pack-Years regression experiments on the CAT dataset.

Education

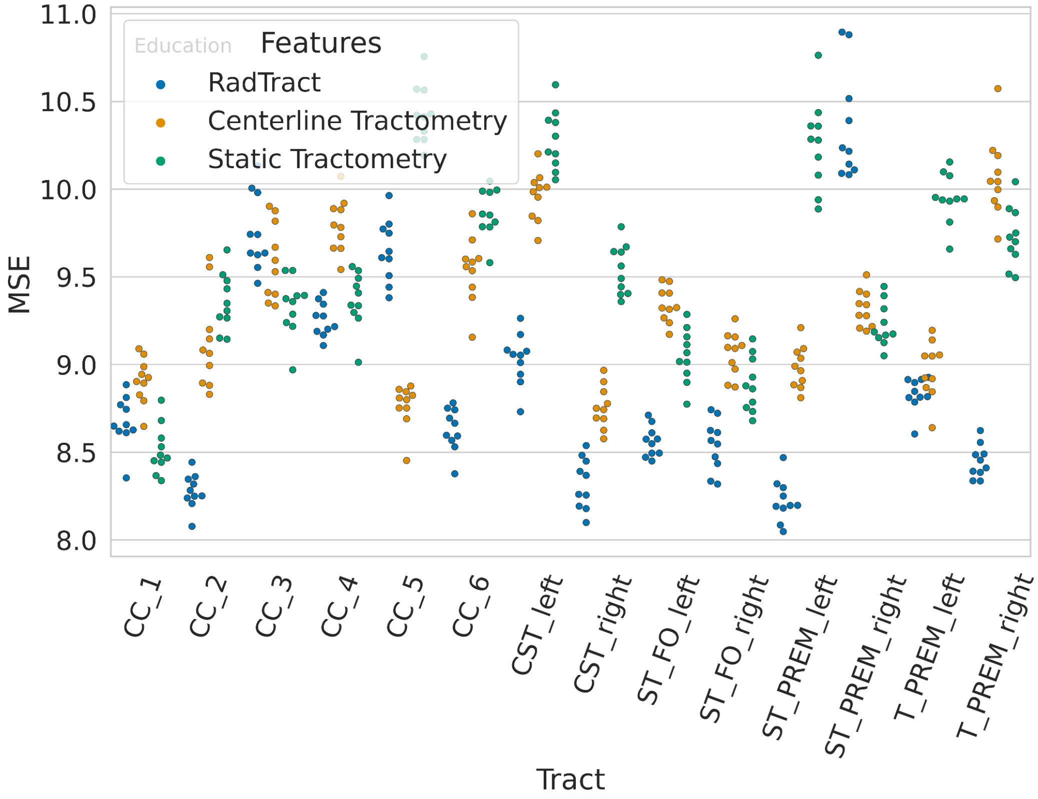

Supplementary Figure 15. Mean squared errors for the Education regression experiments on the CAT dataset.

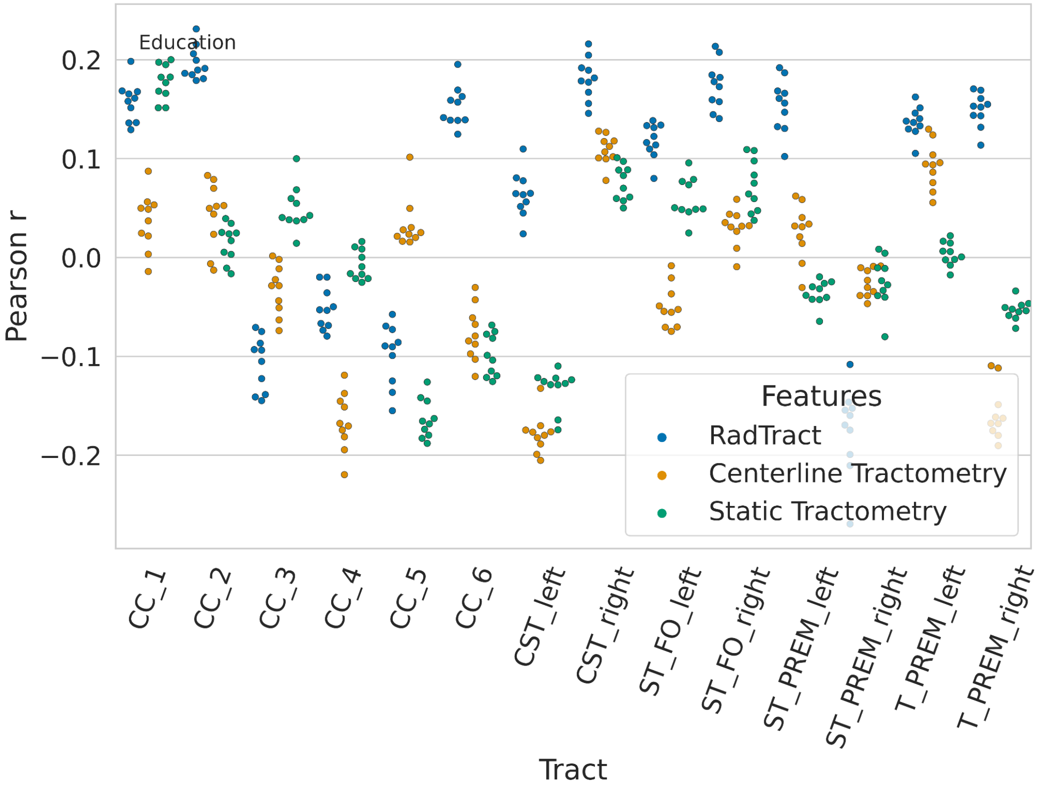

Supplementary Figure 16. Pearson's correlations for the Education regression experiments on the CAT dataset.

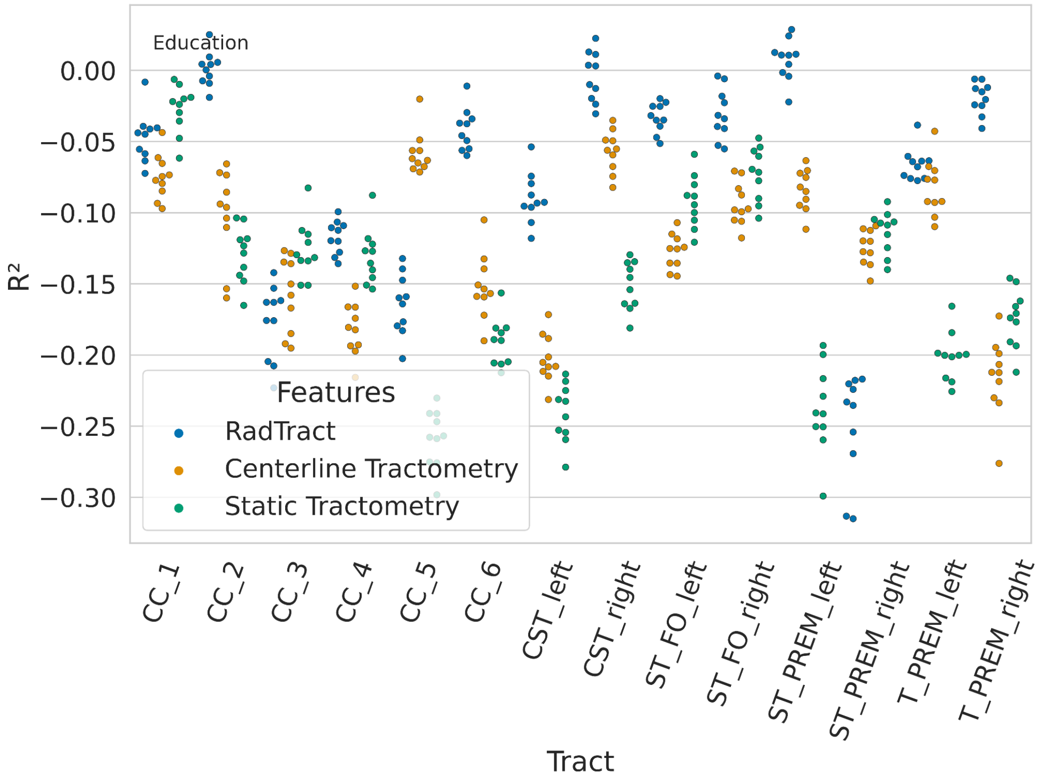

Supplementary Figure 17. Coefficients of determination for the Education regression experiments on the CAT dataset.

BPRS Total

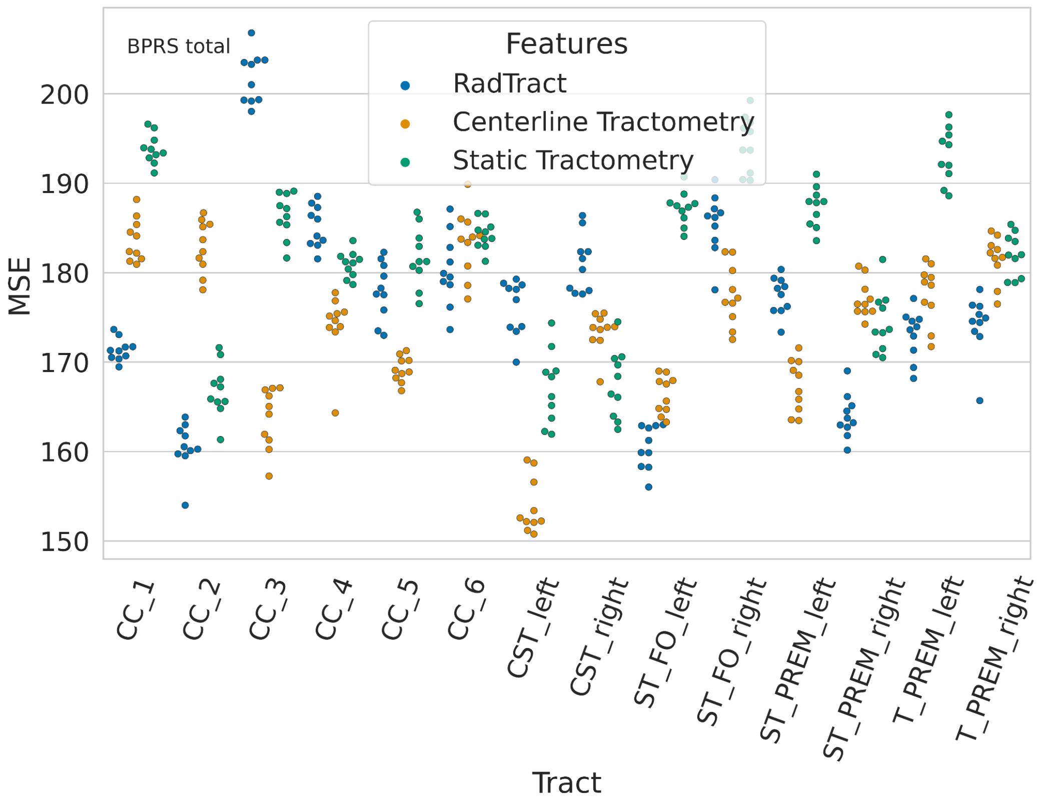

Supplementary Figure 18. Mean squared errors for the BPRS Total regression experiments on the CAT dataset.

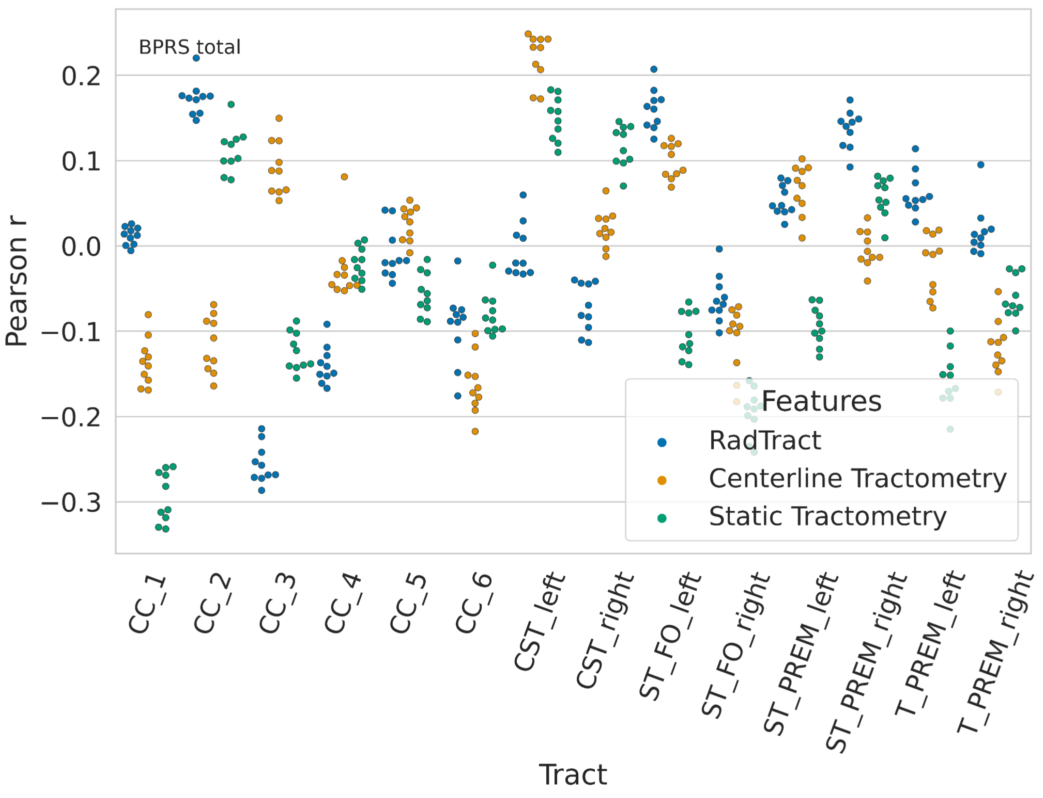

Supplementary Figure 19. Pearson’s correlations for the BPRS Total regression experiments on the CAT dataset.

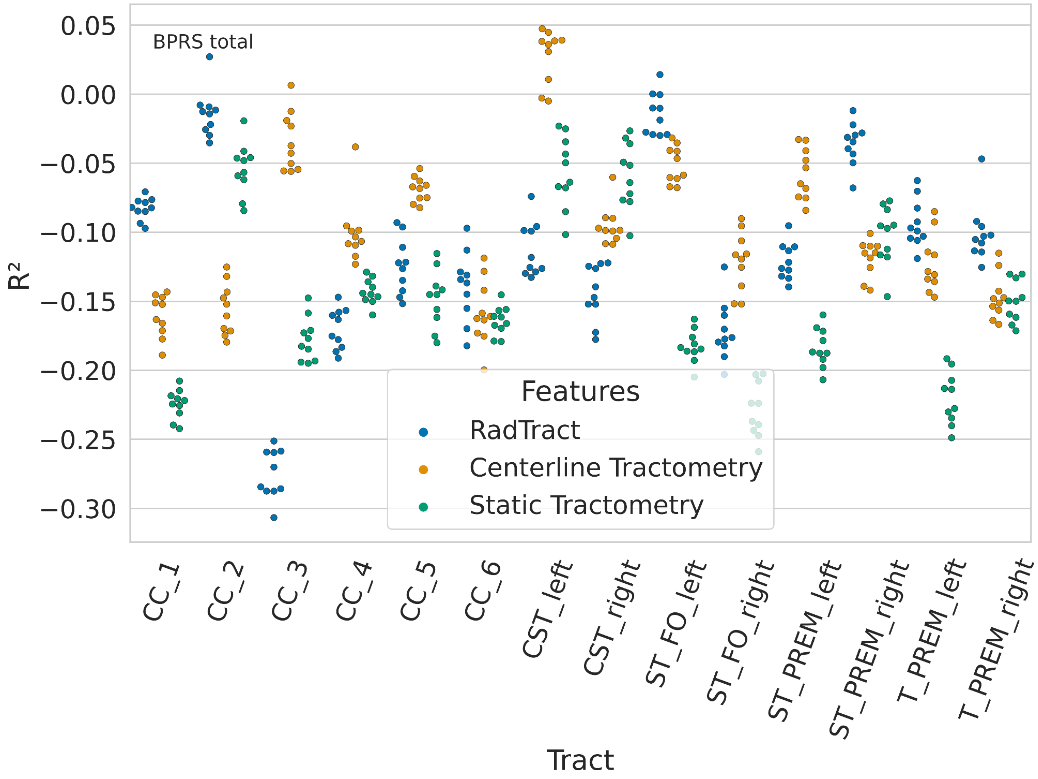

Supplementary Figure 20. Coefficients of determination for the BPRS Total regression experiments on the CAT dataset.

PANSS Total

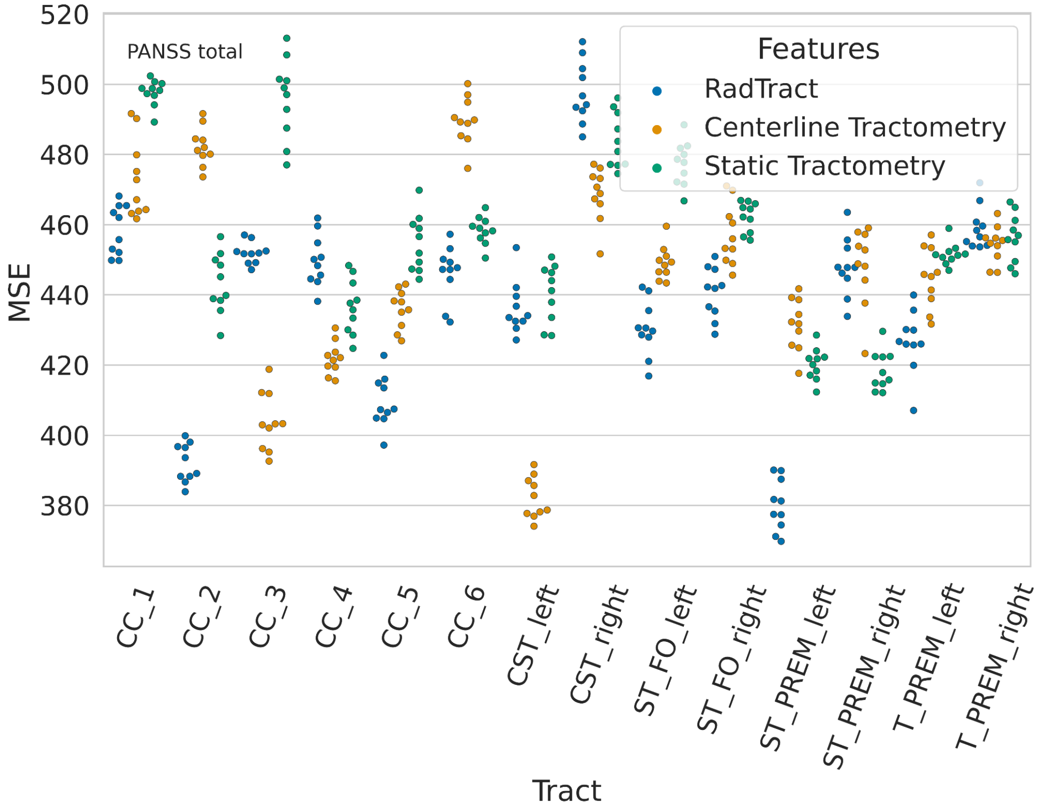

Supplementary Figure 21. Mean squared errors for the PANSS Total regression experiments on the CAT dataset.

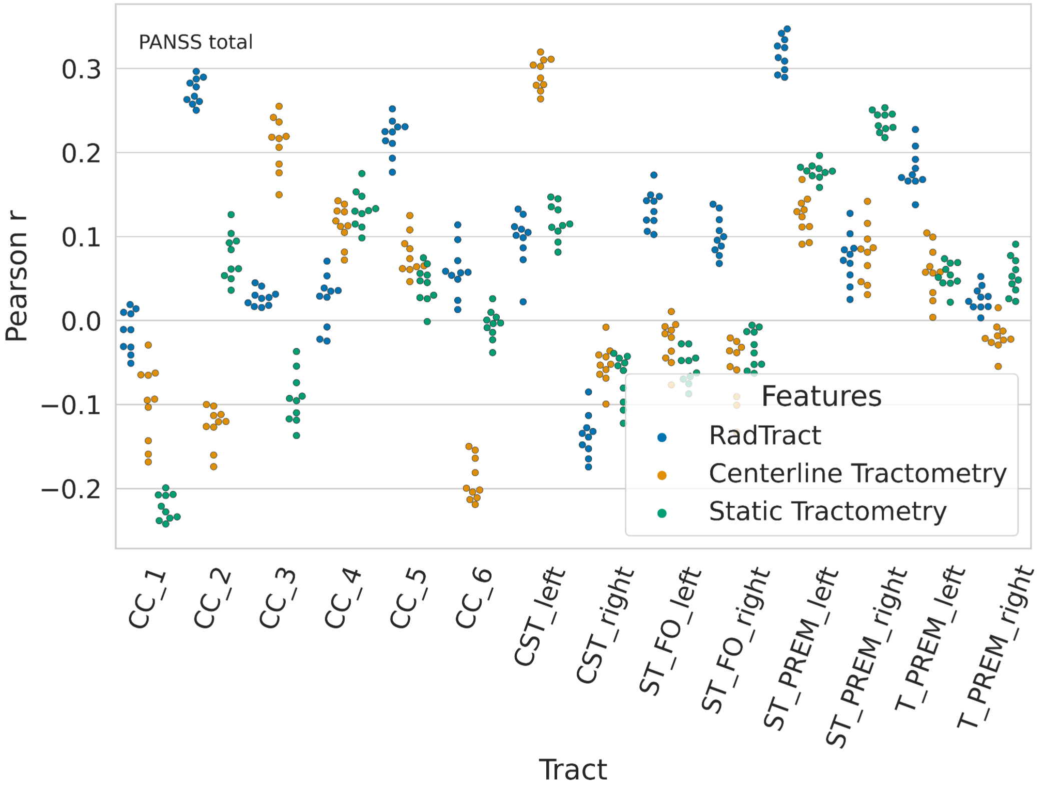

Supplementary Figure 22. Pearson’s correlations for the PANSS Total regression experiments on the CAT dataset.

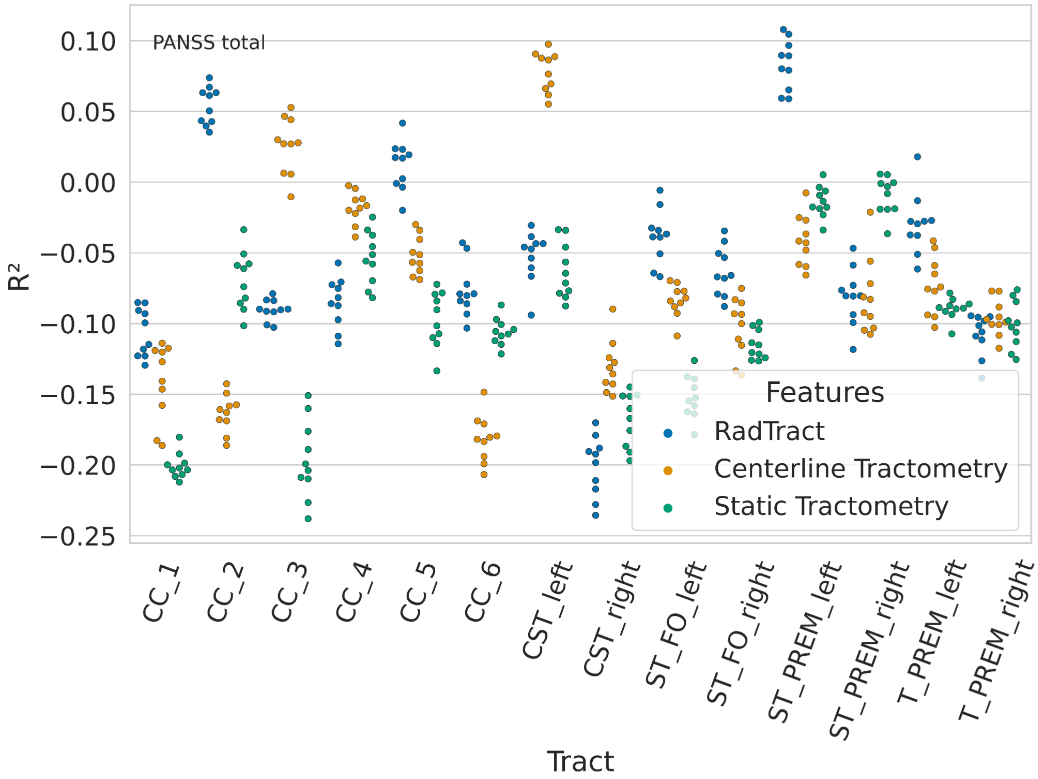

Supplementary Figure 23. Coefficients of determination for the PANSS Total regression experiments on the CAT dataset.

GAF

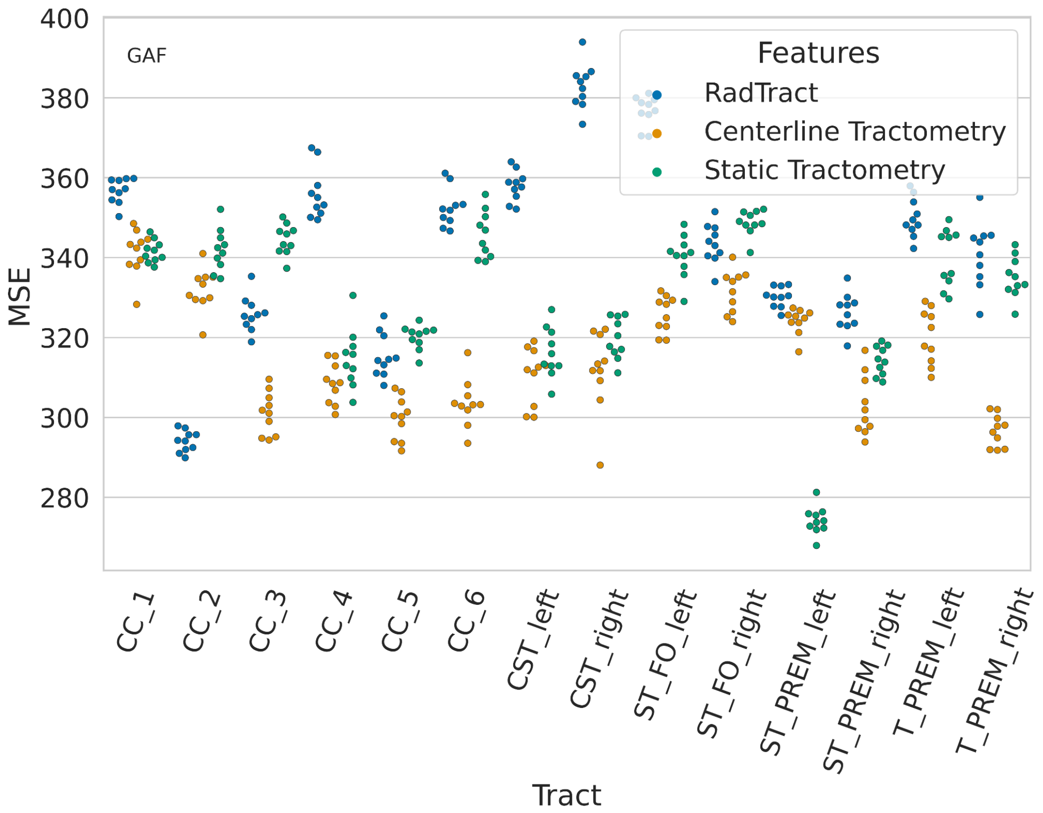

Supplementary Figure 24. Mean squared errors for the GAF regression experiments on the CAT dataset.

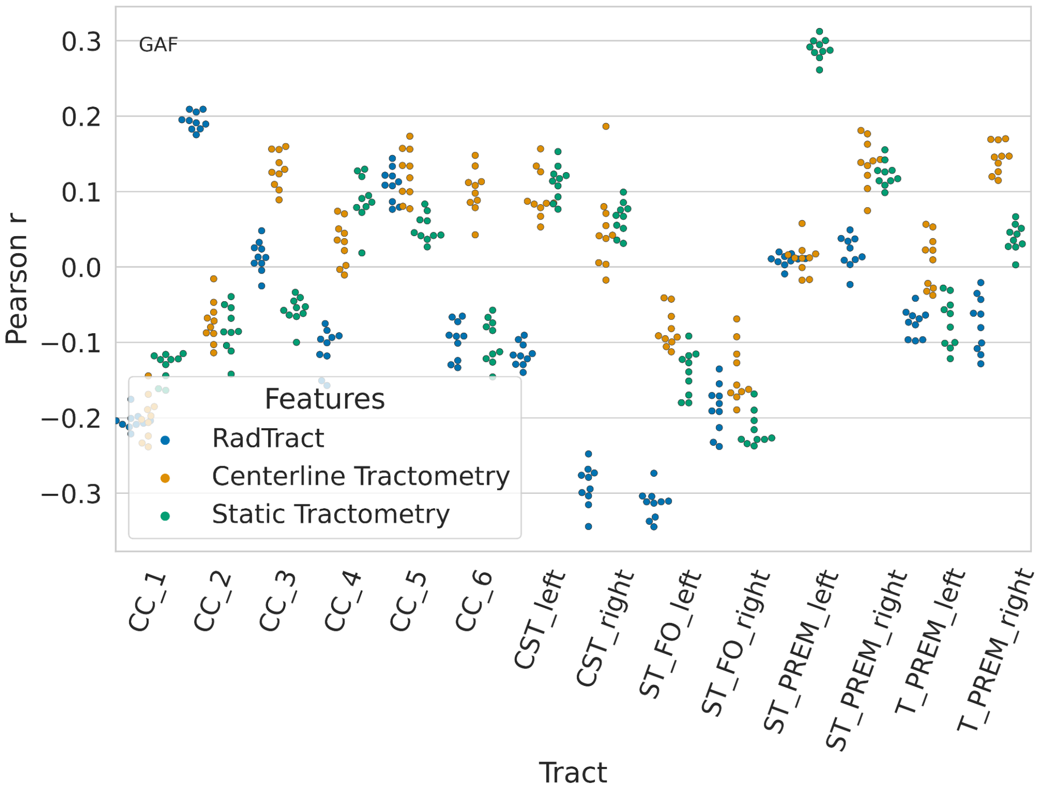

Supplementary Figure 25. Pearson’s correlations for the GAF regression experiments on the CAT dataset.

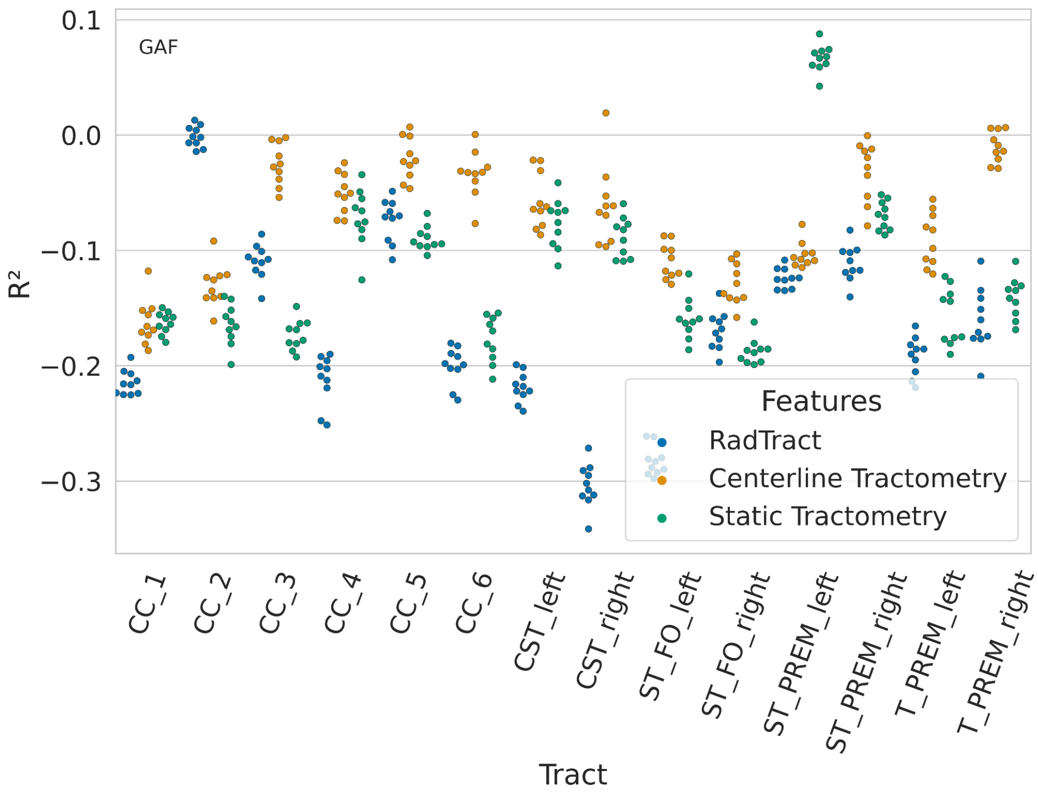

Supplementary Figure 26. Coefficients of determination for the GAF regression experiments on the CAT dataset.

OLZe

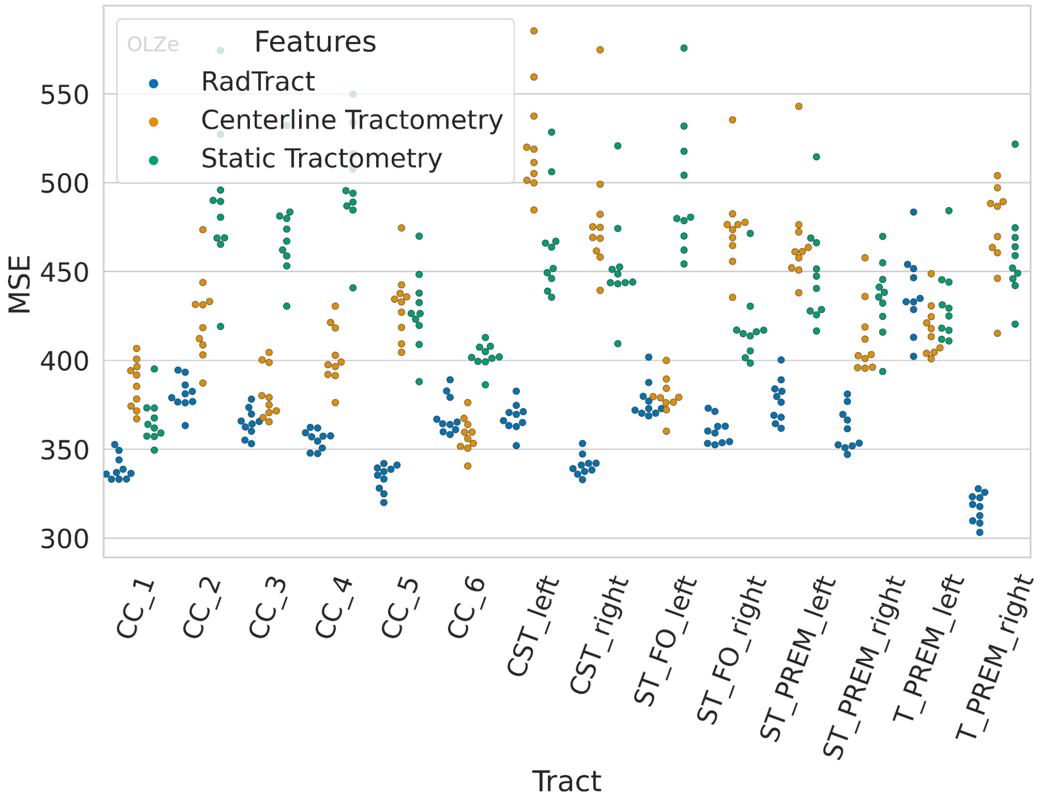

Supplementary Figure 27. Mean squared errors for the OLZe regression experiments on the CAT dataset.

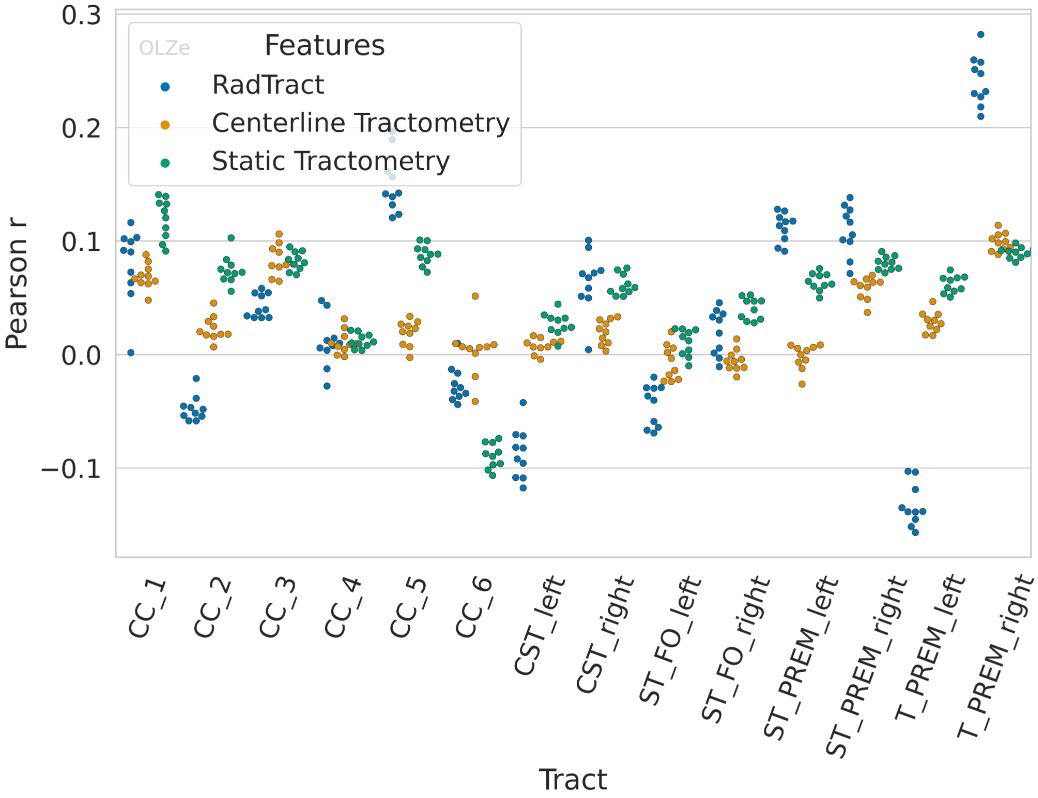

Supplementary Figure 28. Pearson's correlations for the OLZe regression experiments on the CAT dataset.

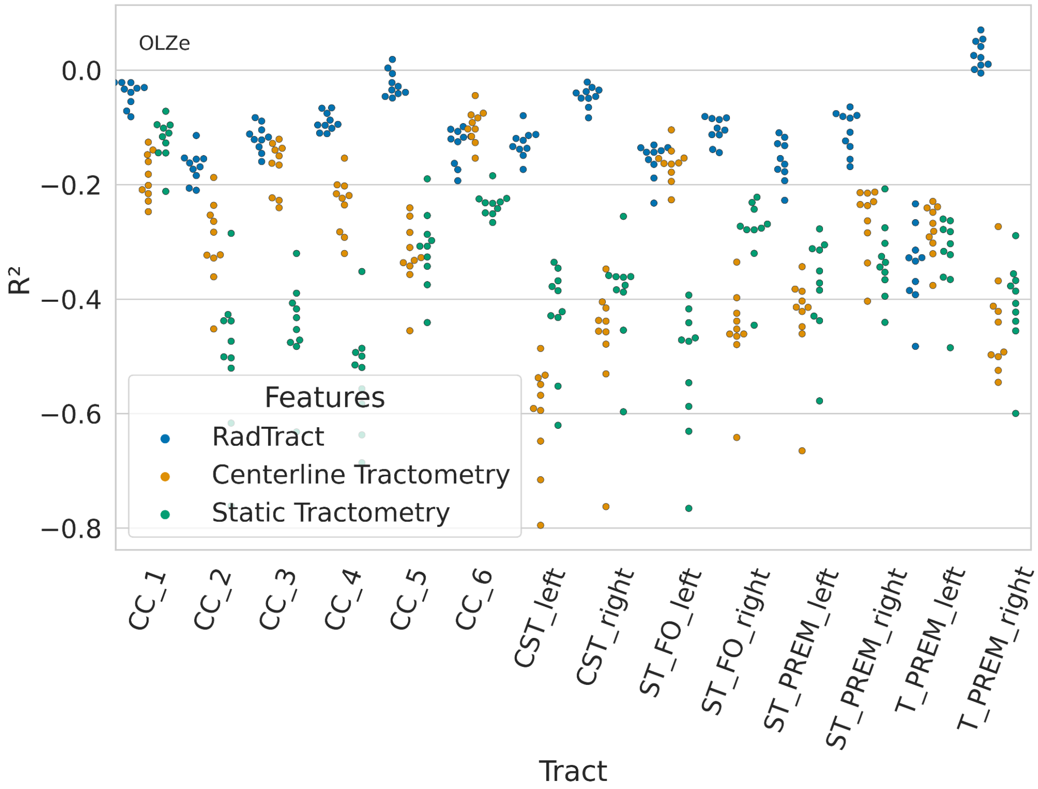

Supplementary Figure 29. Coefficients of determination for the OLZe regression experiments on the CAT dataset.

Supplementary Note 4 – Top ten features per dataset

SCHZ

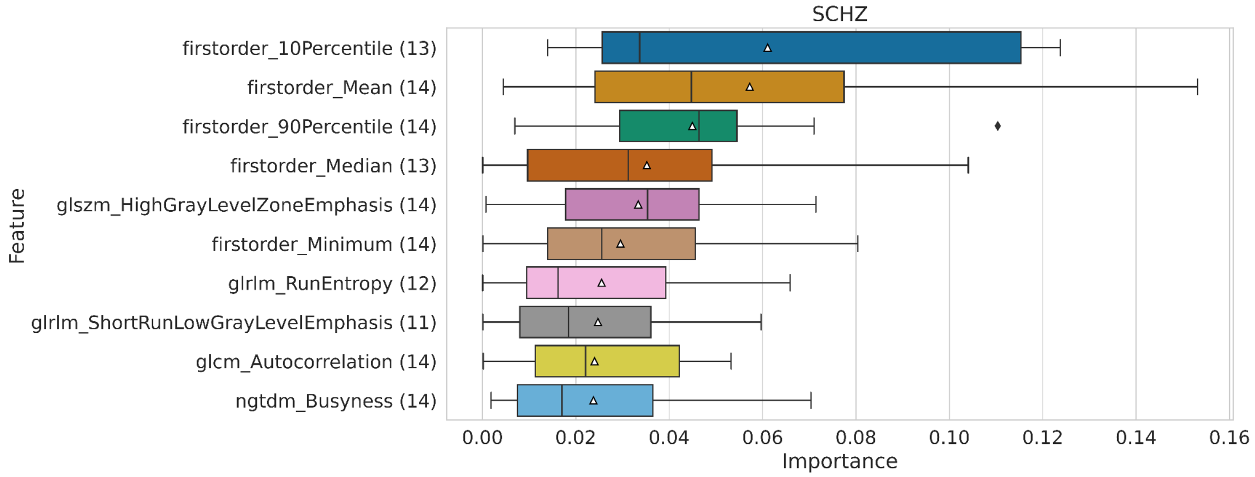

Supplementary Figure 30. Top ten most important features across all tracts of the SCHZ dataset. The number of samples is indicated in brackets in the graph legend.

CAT

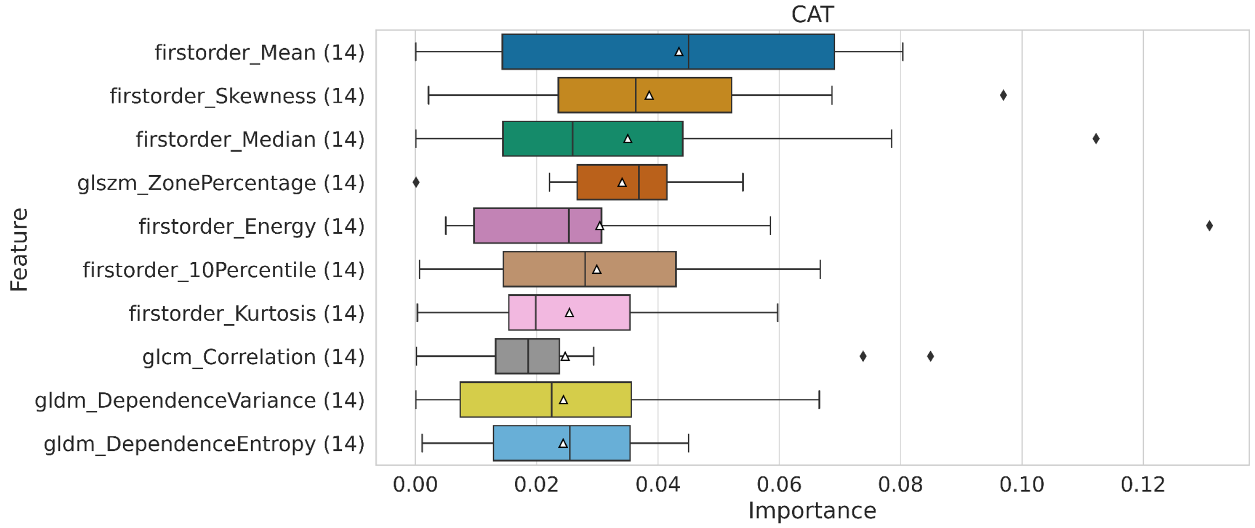

Supplementary Figure 31. Top ten most important features across all tracts of the CAT dataset. The number of samples is indicated in brackets in the graph legend.

ADNI

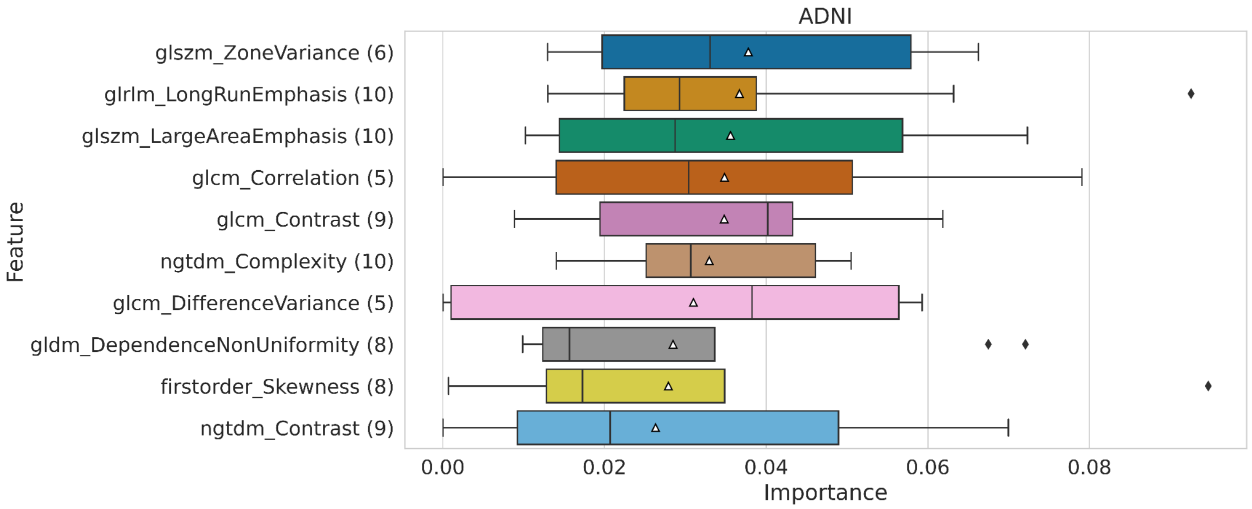

Supplementary Figure 32. Top ten most important features across all tracts of the ADNI dataset. The number of samples is indicated in brackets in the graph legend.

PPMI

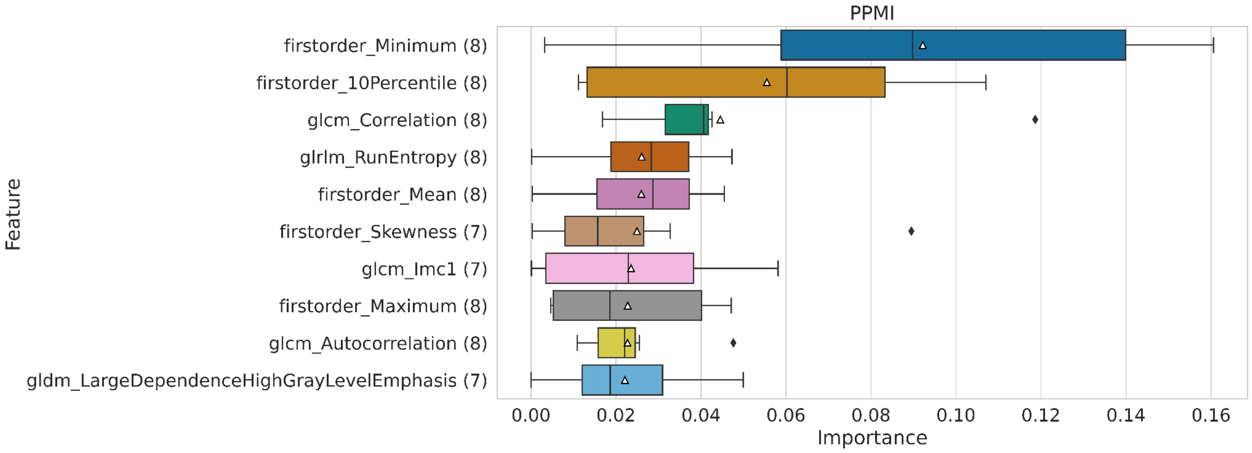

Supplementary Figure 33. Top ten most important features across all tracts of the PPMI dataset. The number of samples is indicated in brackets in the graph legend.

Supplementary Note 5 – Parcel Importance

SCHZ

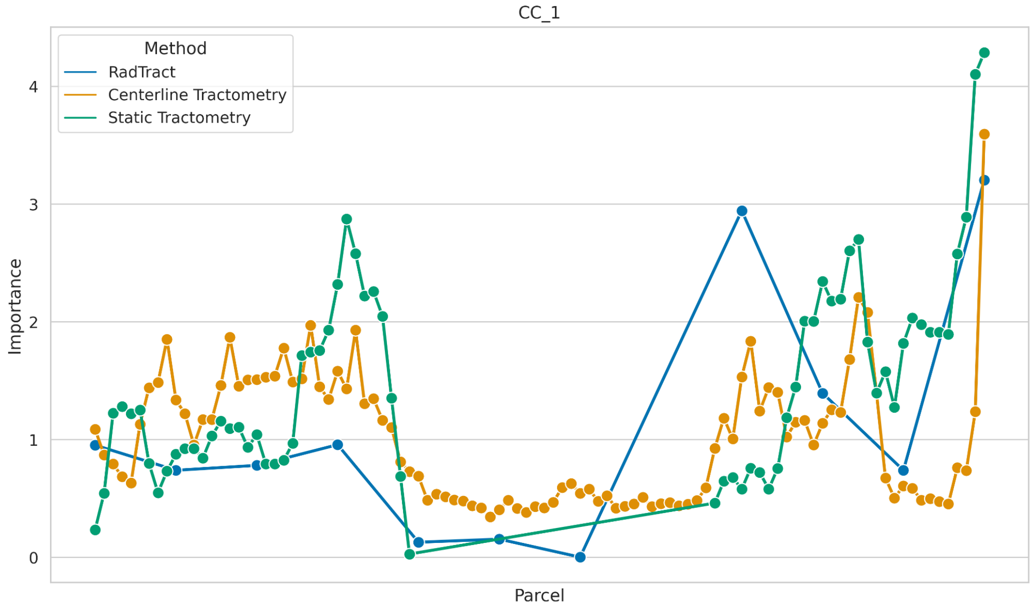

Supplementary Figure 34. Parcel importances for all three methods along the tract (SCHZ, CC_1), aggregated over features.

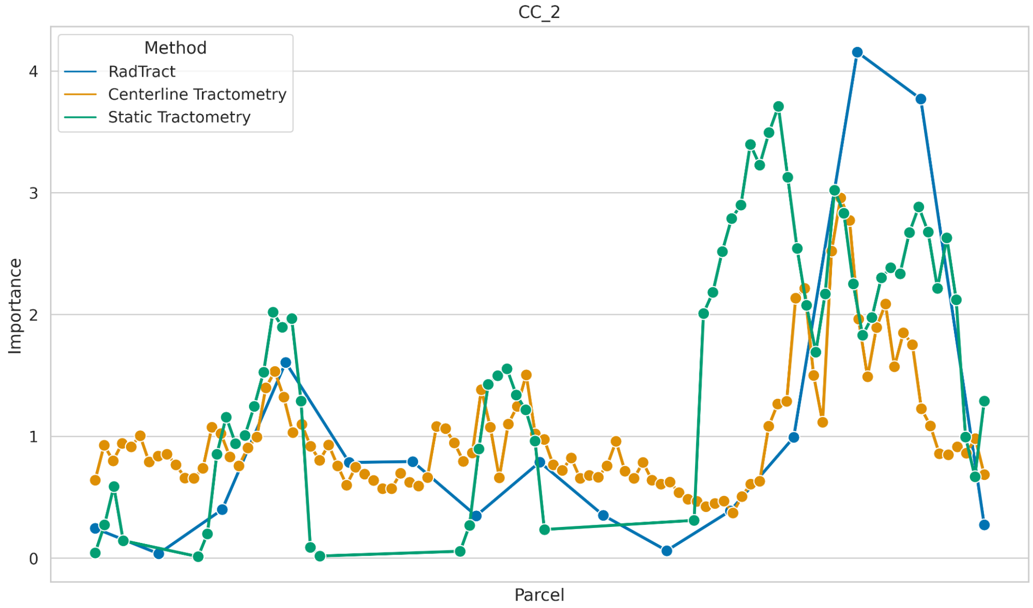

Supplementary Figure 35. Parcel importances for all three methods along the tract (SCHZ, CC_2), aggregated over features.

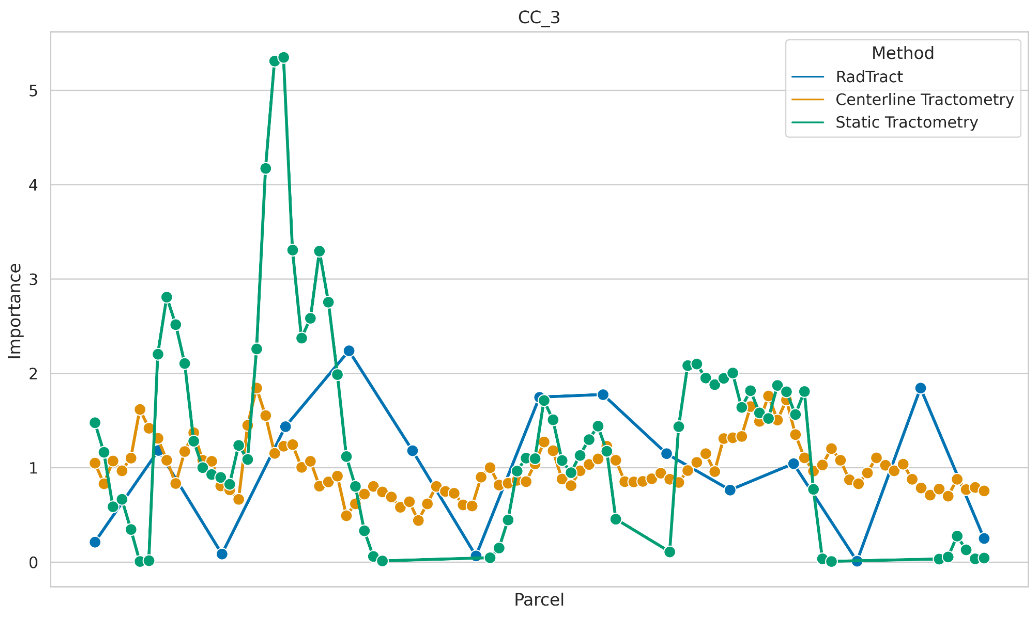

Supplementary Figure 36. Parcel importances for all three methods along the tract (SCHZ, CC_3), aggregated over features.

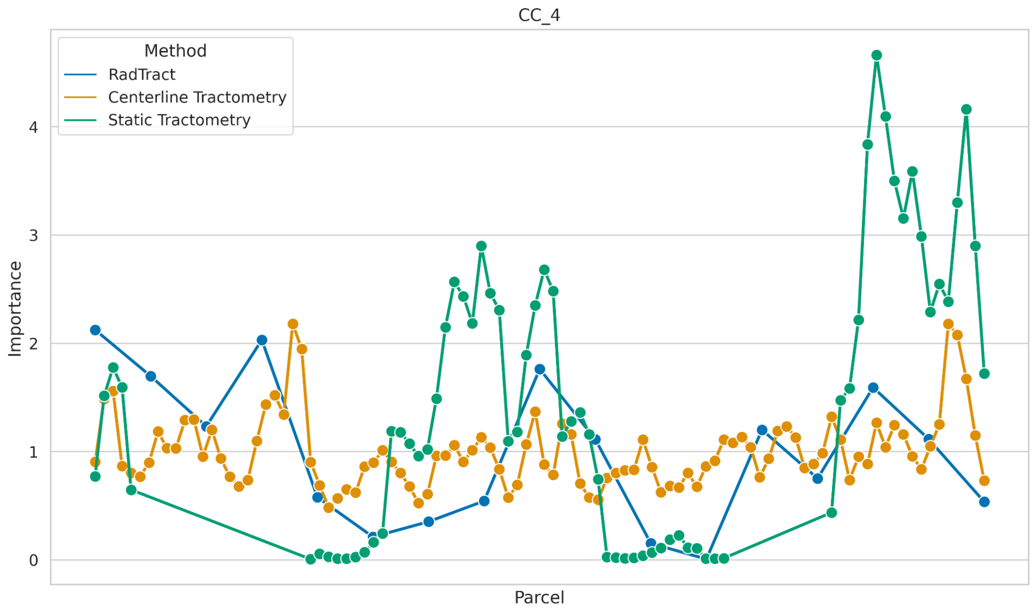

Supplementary Figure 37. Parcel importances for all three methods along the tract (SCHZ, CC_4), aggregated over features.

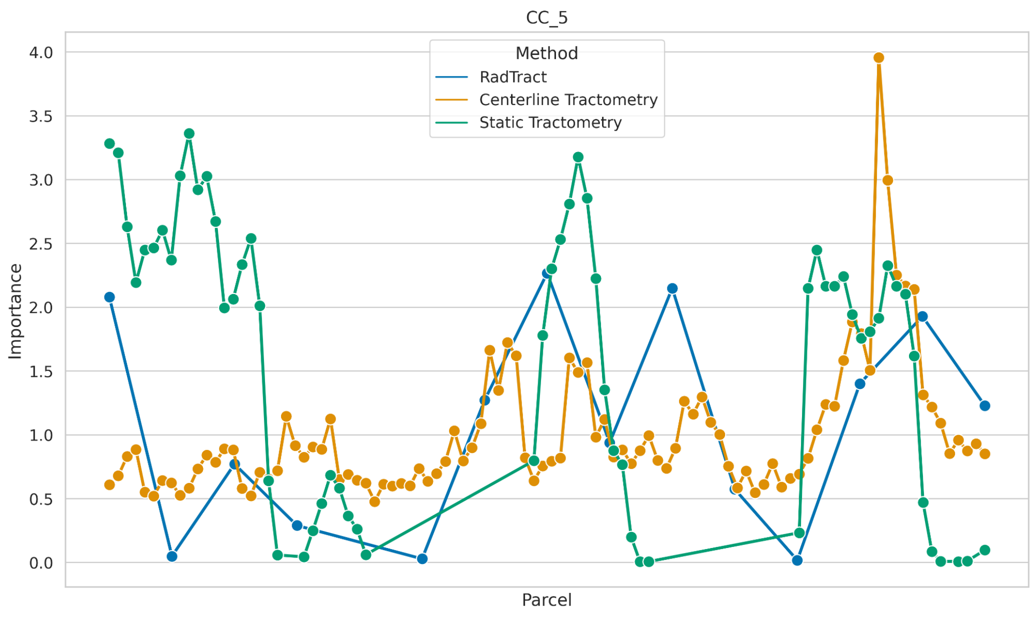

Supplementary Figure 38. Parcel importances for all three methods along the tract (SCHZ, CC_5), aggregated over features.

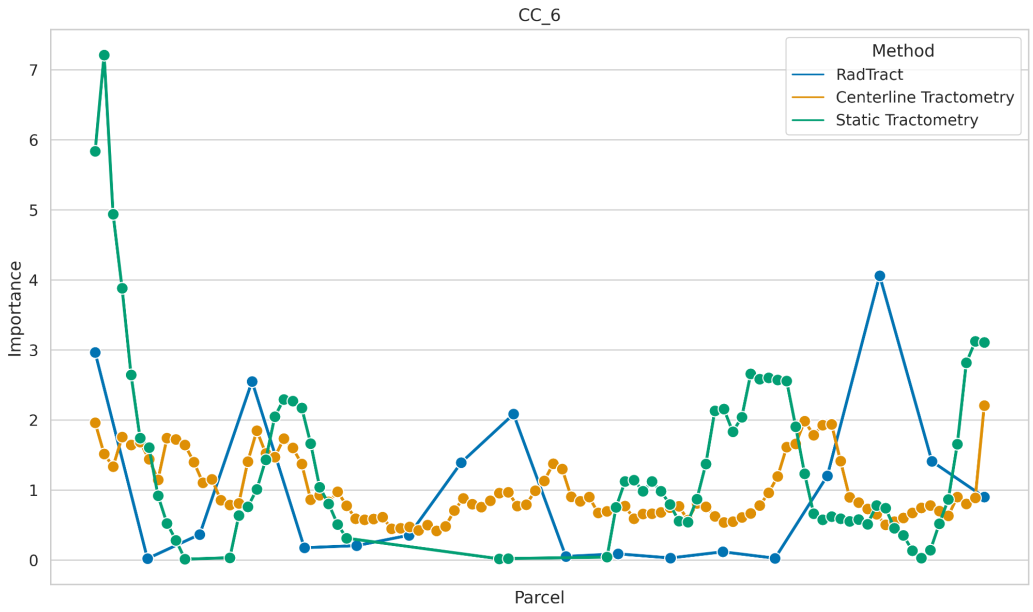

Supplementary Figure 39. Parcel importances for all three methods along the tract (SCHZ, CC_6), aggregated over features.

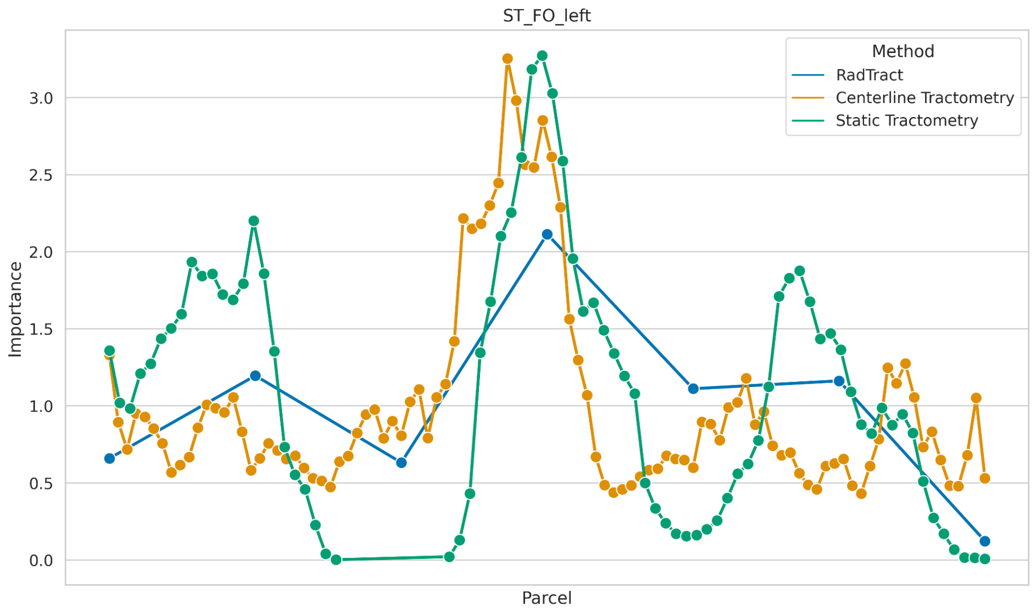

Supplementary Figure 40. Parcel importances for all three methods along the tract (SCHZ, ST_FO_left), aggregated over features.

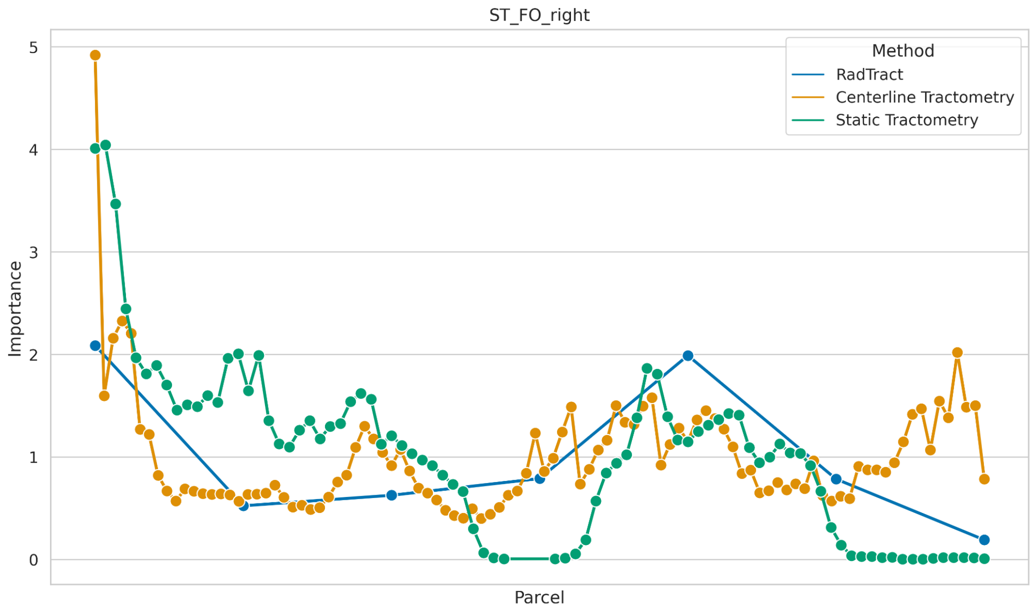

Supplementary Figure 41. Parcel importances for all three methods along the tract (SCHZ, ST_FO_right), aggregated over features.

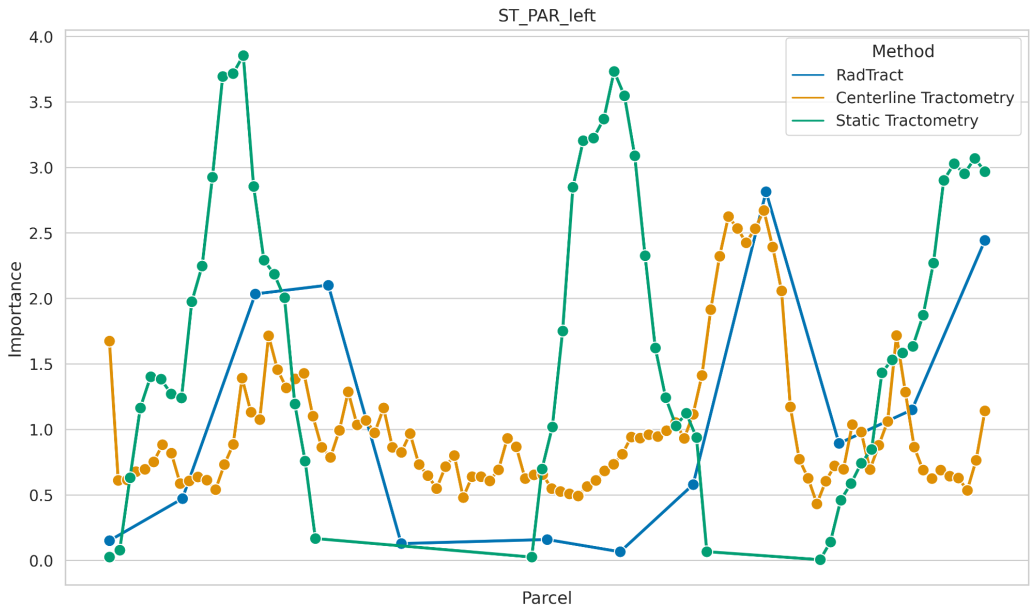

Supplementary Figure 42. Parcel importances for all three methods along the tract (SCHZ, ST_PAR_left), aggregated over features.

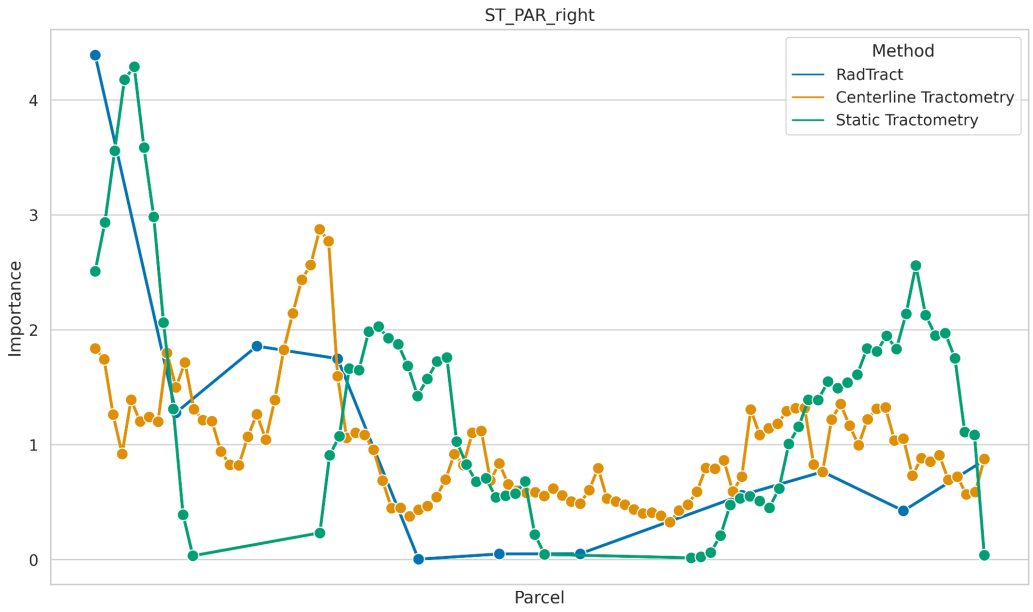

Supplementary Figure 43. Parcel importances for all three methods along the tract (SCHZ, ST_PAR_right), aggregated over features.

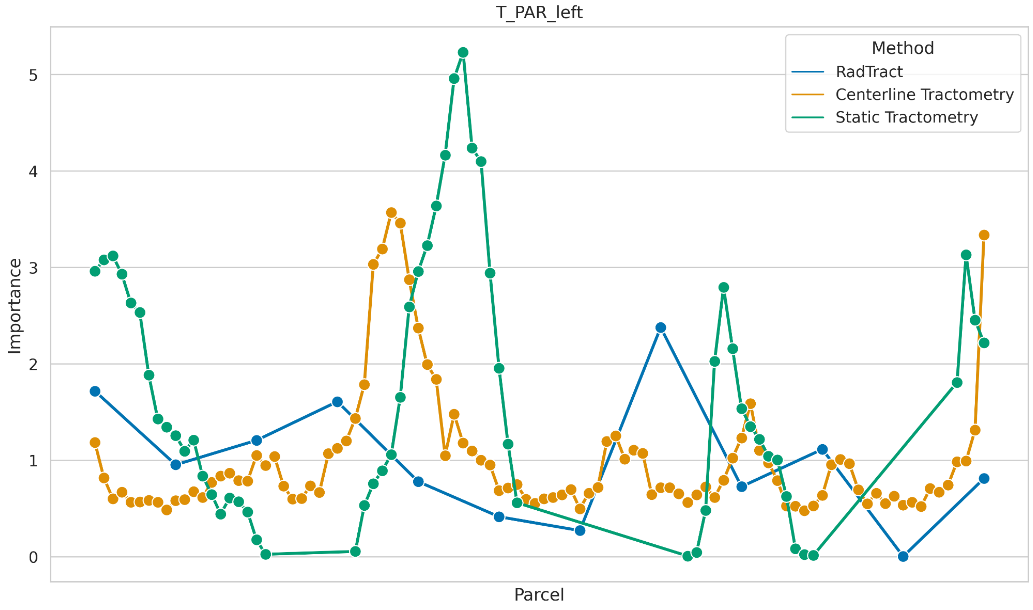

Supplementary Figure 44. Parcel importances for all three methods along the tract (SCHZ, T_PAR_left), aggregated over features.

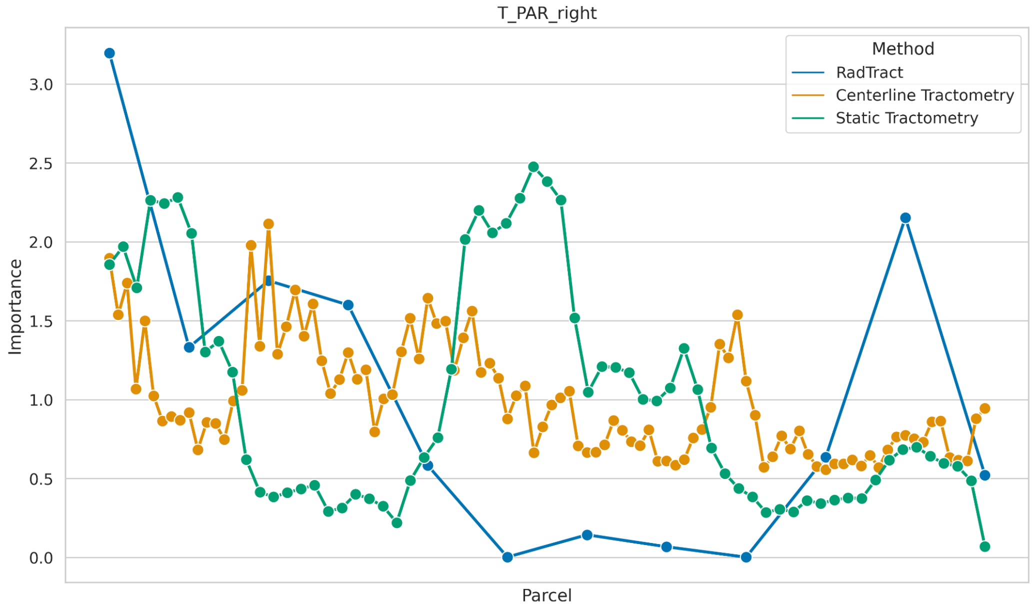

Supplementary Figure 45. Parcel importances for all three methods along the tract (SCHZ, T_PAR_right), aggregated over features.

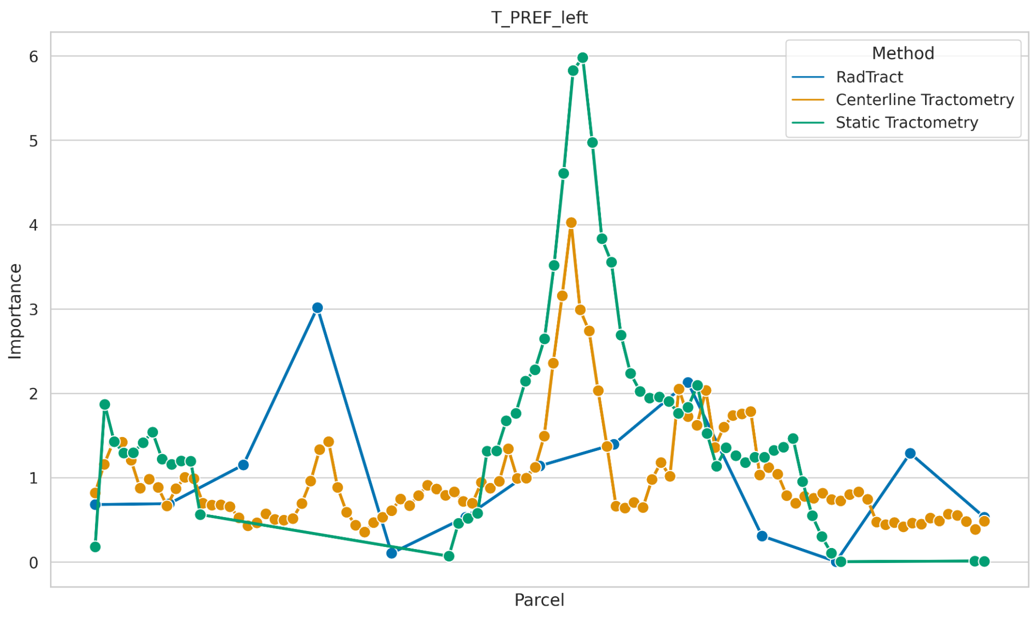

Supplementary Figure 46. Parcel importances for all three methods along the tract (SCHZ, T_PREF_left), aggregated over features.

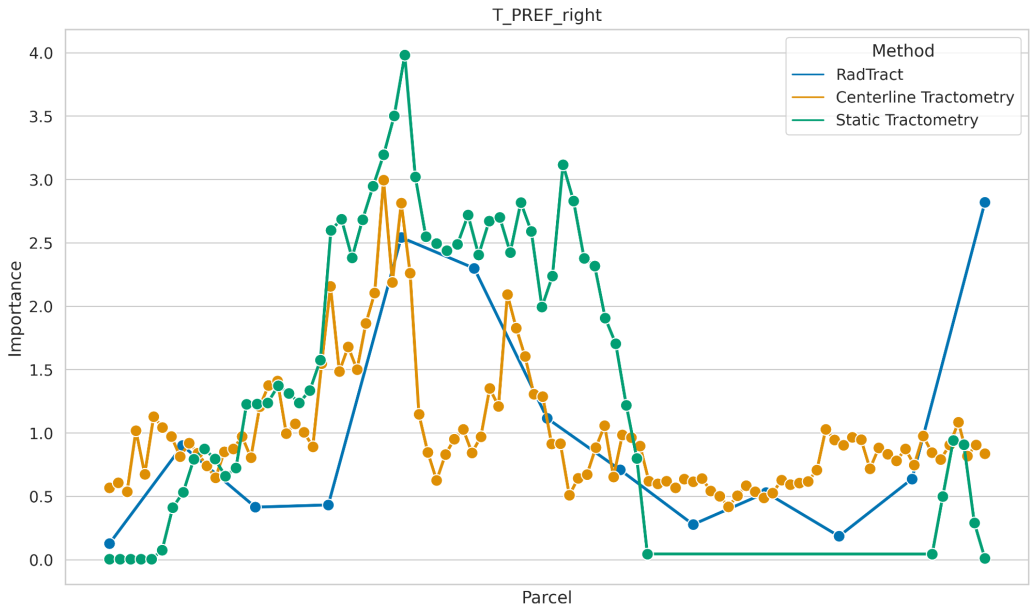

Supplementary Figure 47. Parcel importances for all three methods along the tract (SCHZ, T_PREF_right), aggregated over features.

CAT

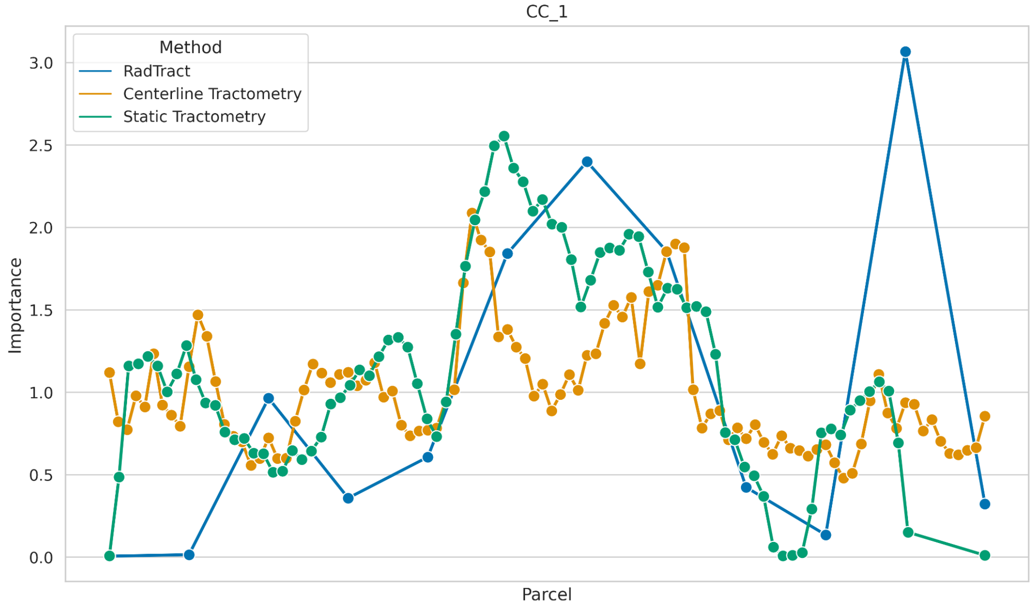

Supplementary Figure 48. Parcel importances for all three methods along the tract (CAT, CC_1), aggregated over features.

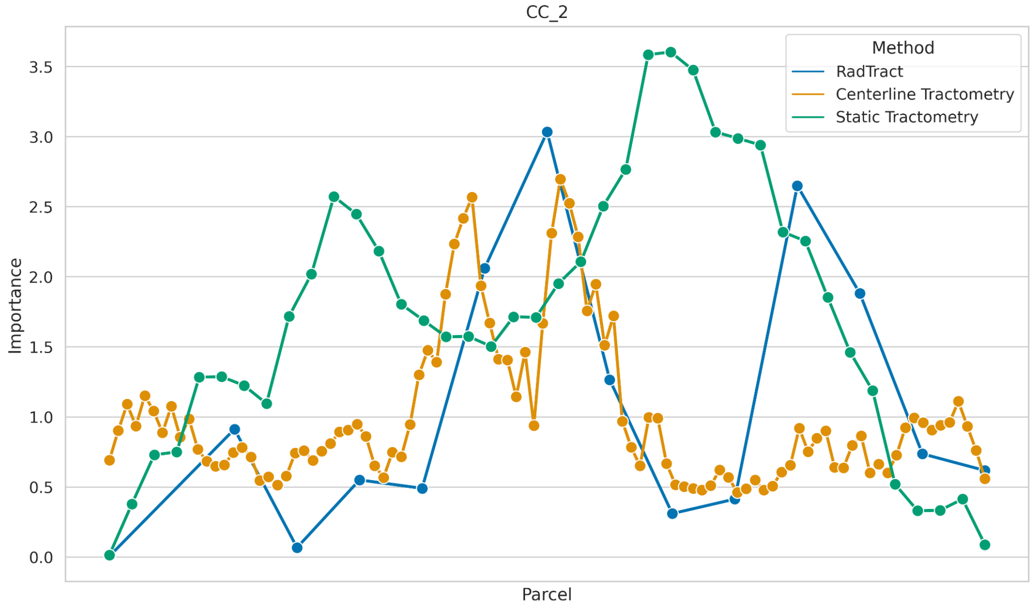

Supplementary Figure 49. Parcel importances for all three methods along the tract (CAT, CC_2), aggregated over features.

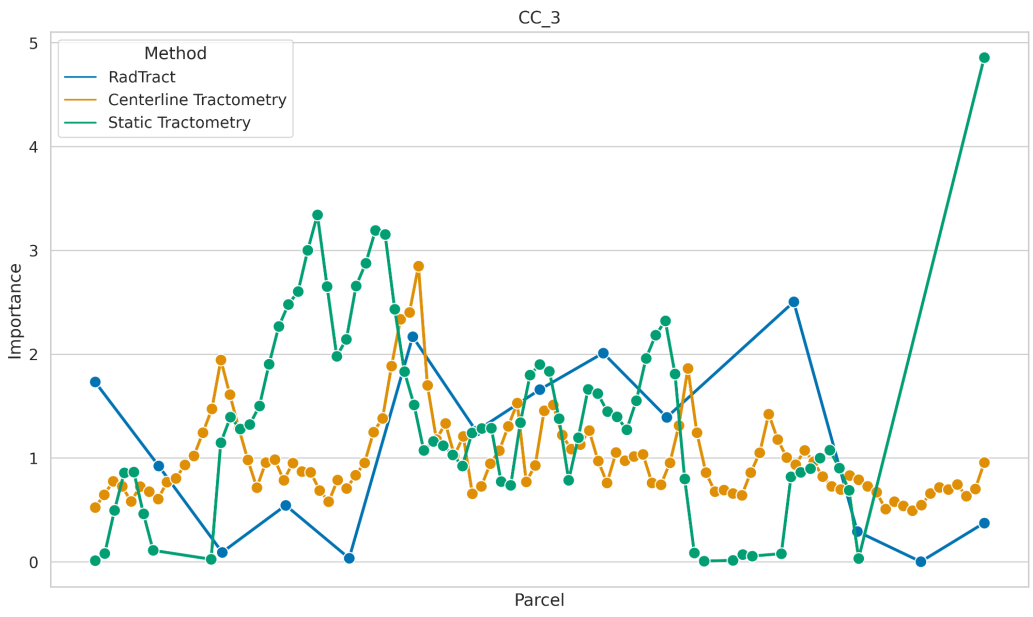

Supplementary Figure 50. Parcel importances for all three methods along the tract (CAT, CC_3), aggregated over features.

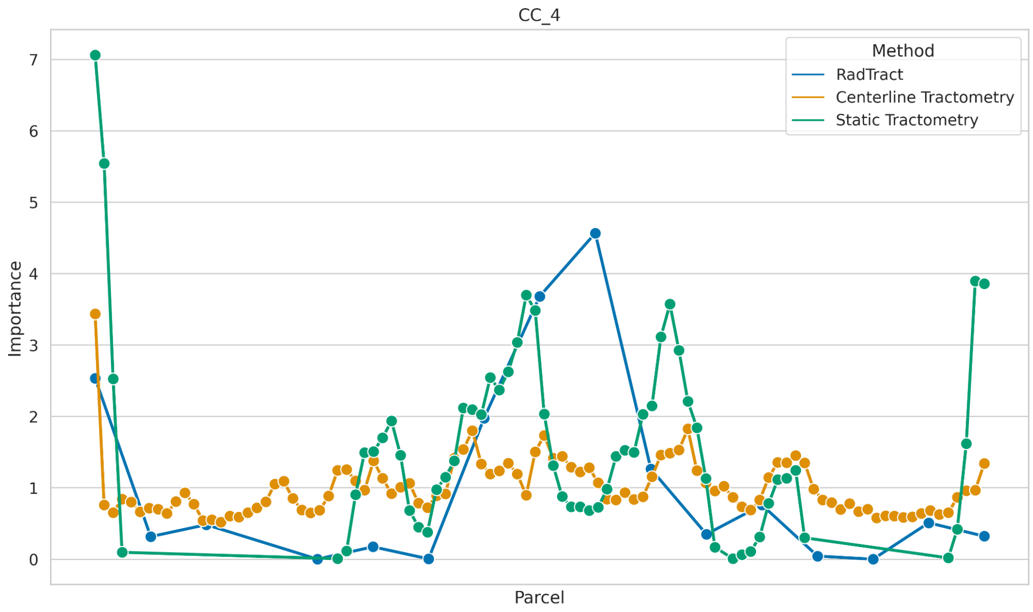

Supplementary Figure 51. Parcel importances for all three methods along the tract (CAT, CC_4), aggregated over features.

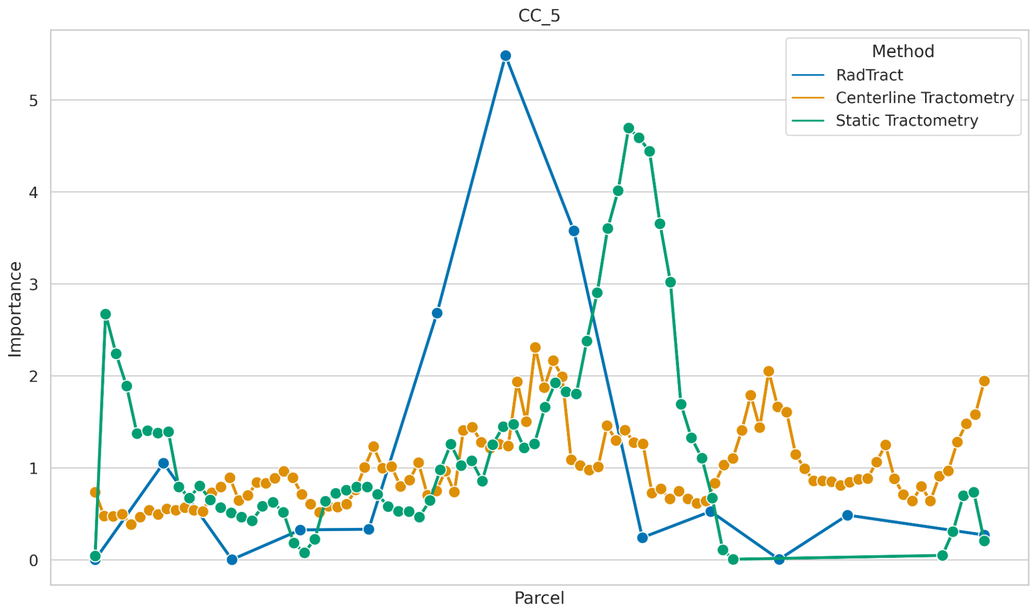

Supplementary Figure 52. Parcel importances for all three methods along the tract (CAT, CC_5), aggregated over features.

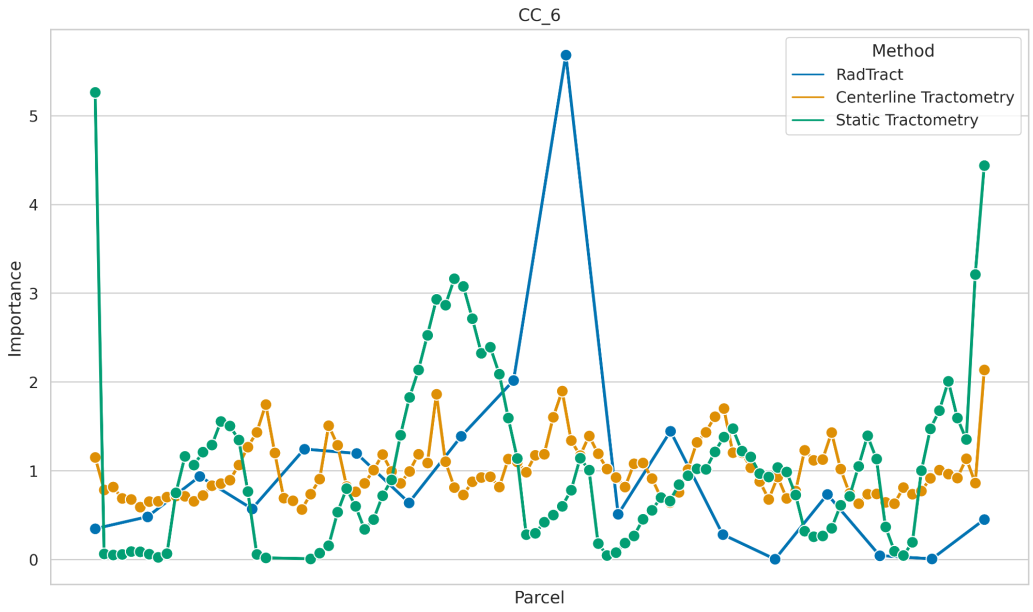

Supplementary Figure 53. Parcel importances for all three methods along the tract (CAT, CC_6), aggregated over features.

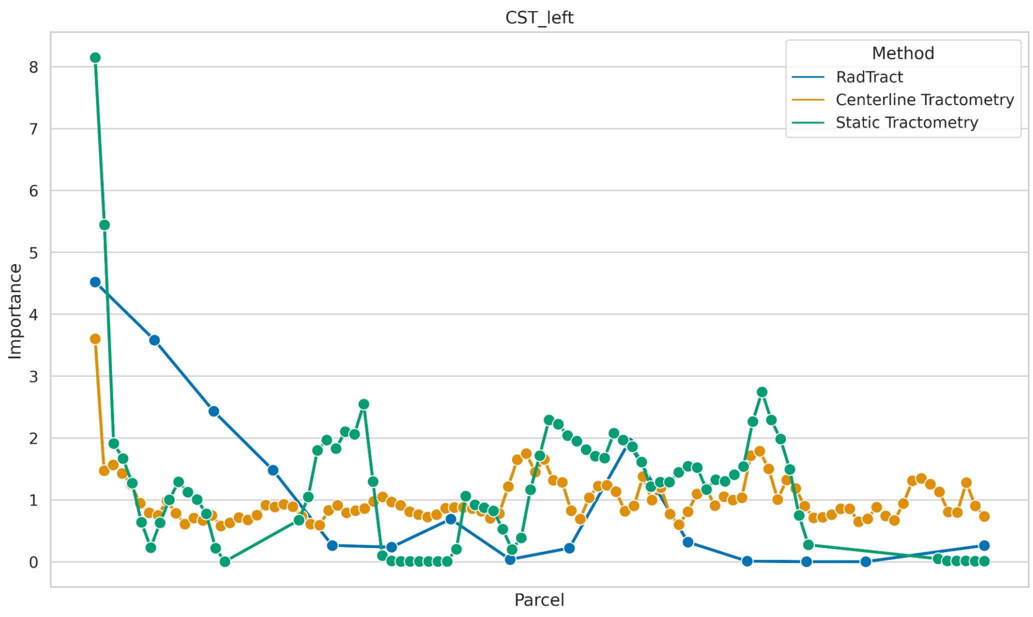

Supplementary Figure 54. Parcel importances for all three methods along the tract (CAT, CST_left), aggregated over features.

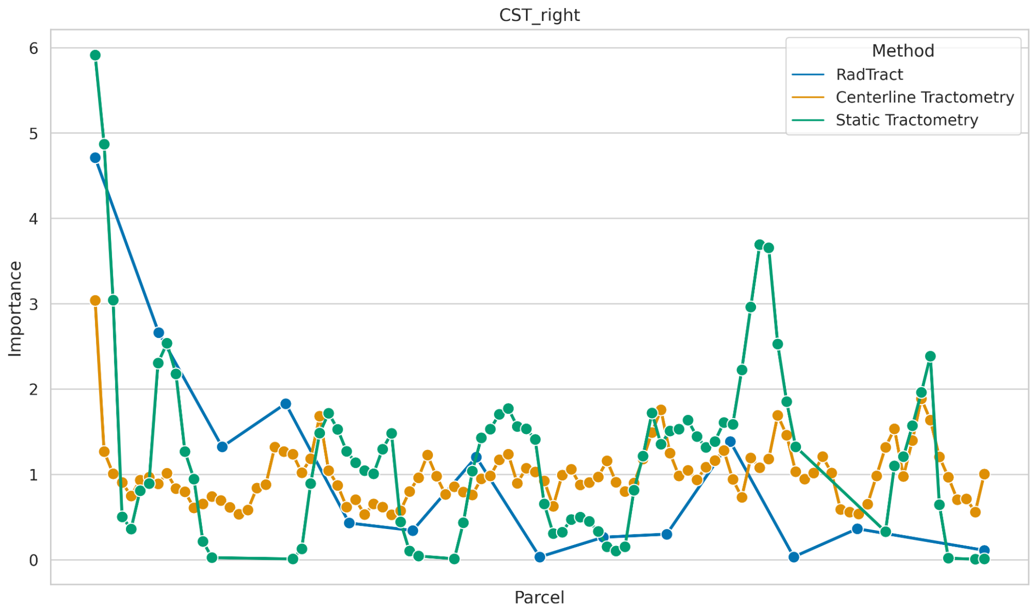

Supplementary Figure 55. Parcel importances for all three methods along the tract (CAT, CST_right), aggregated over features.

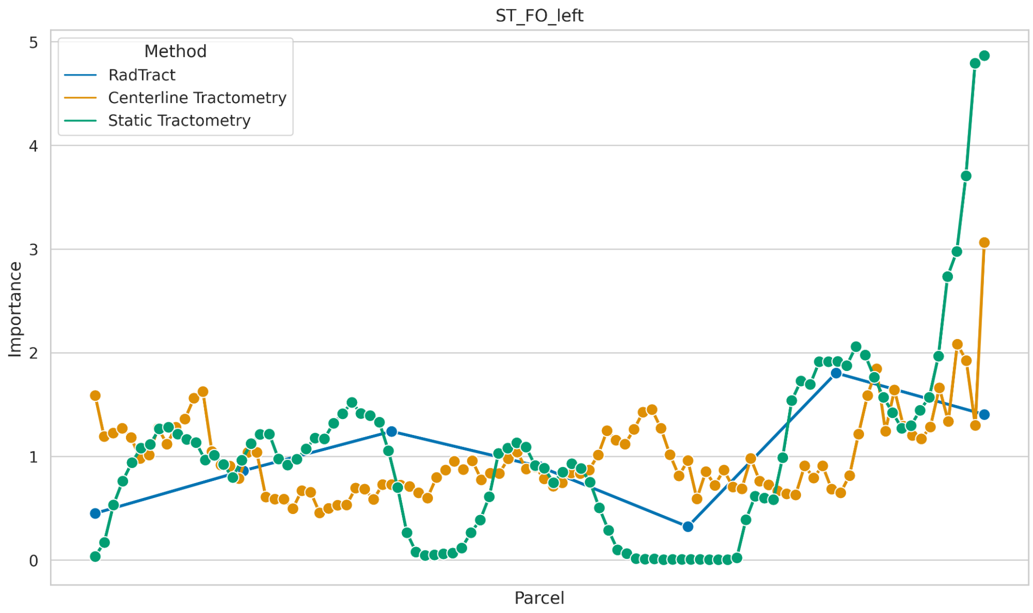

Supplementary Figure 56. Parcel importances for all three methods along the tract (CAT, ST_FO_left), aggregated over features.

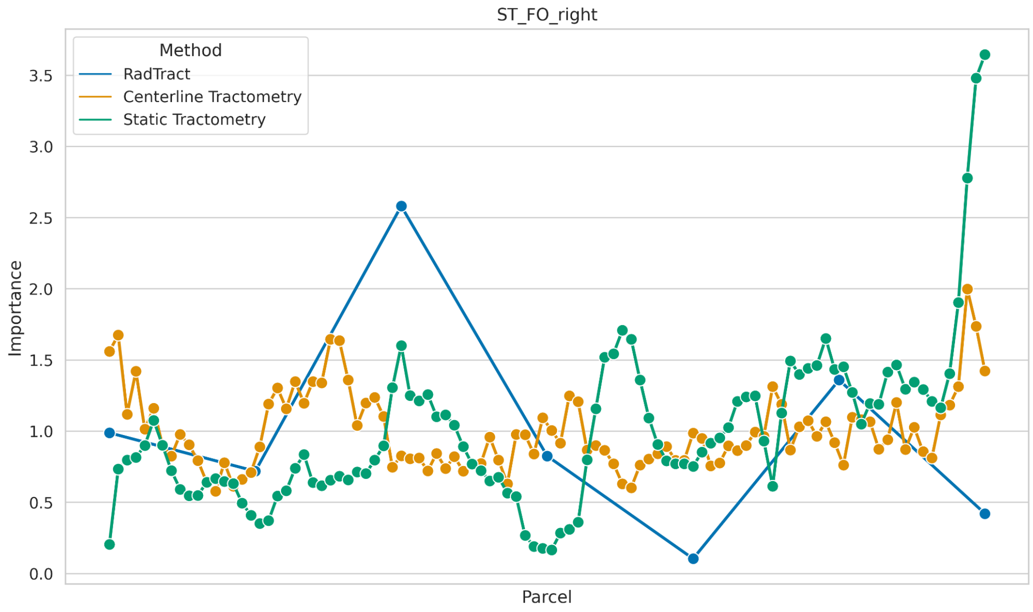

Supplementary Figure 57. Parcel importances for all three methods along the tract (CAT, ST_FO_right), aggregated over features.

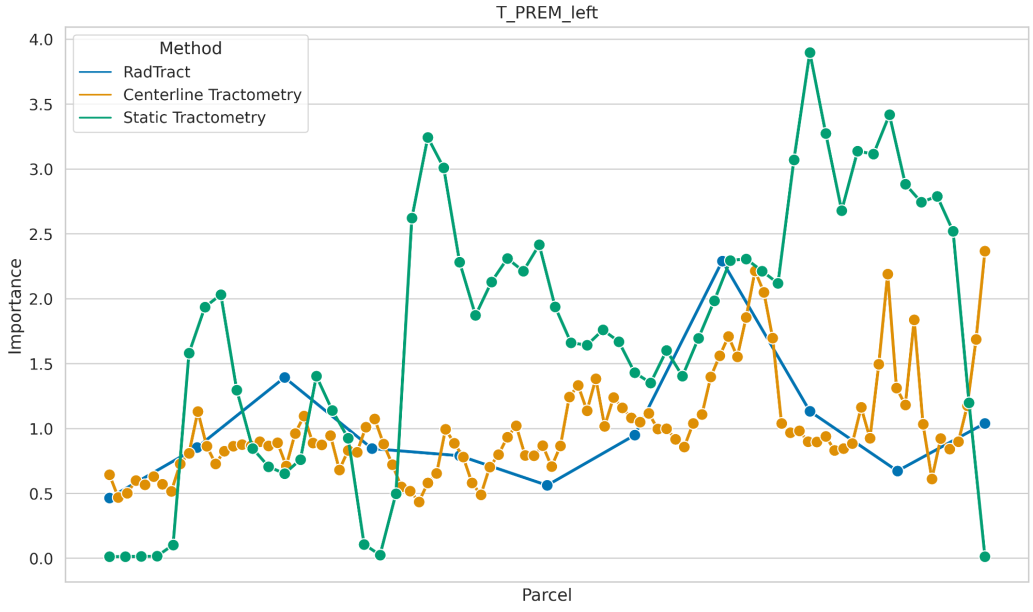

Supplementary Figure 58. Parcel importances for all three methods along the tract (CAT, T_PREM_left), aggregated over features.

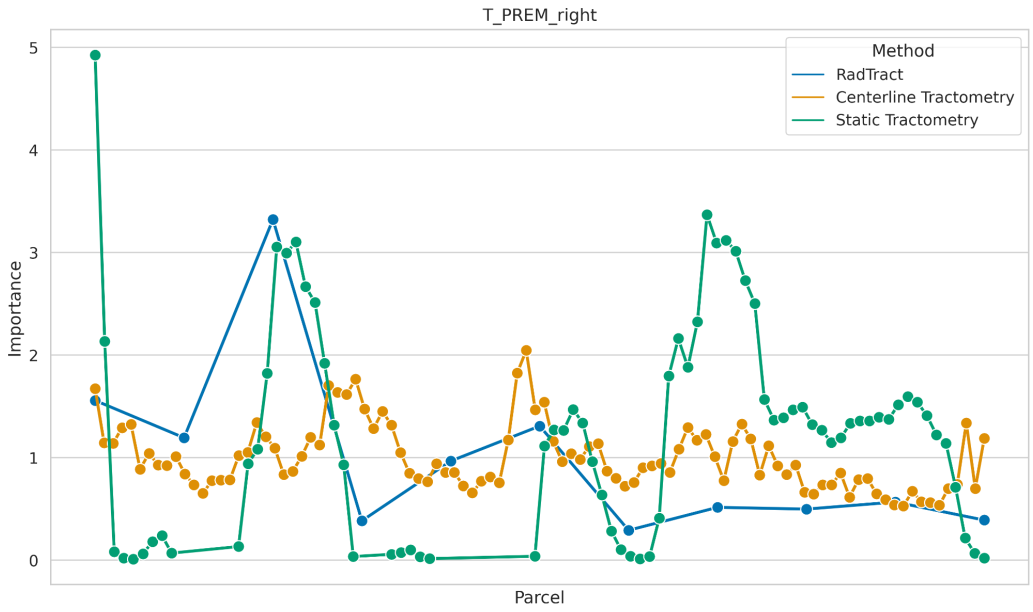

Supplementary Figure 59. Parcel importances for all three methods along the tract (CAT, T_PREM_right), aggregated over features.

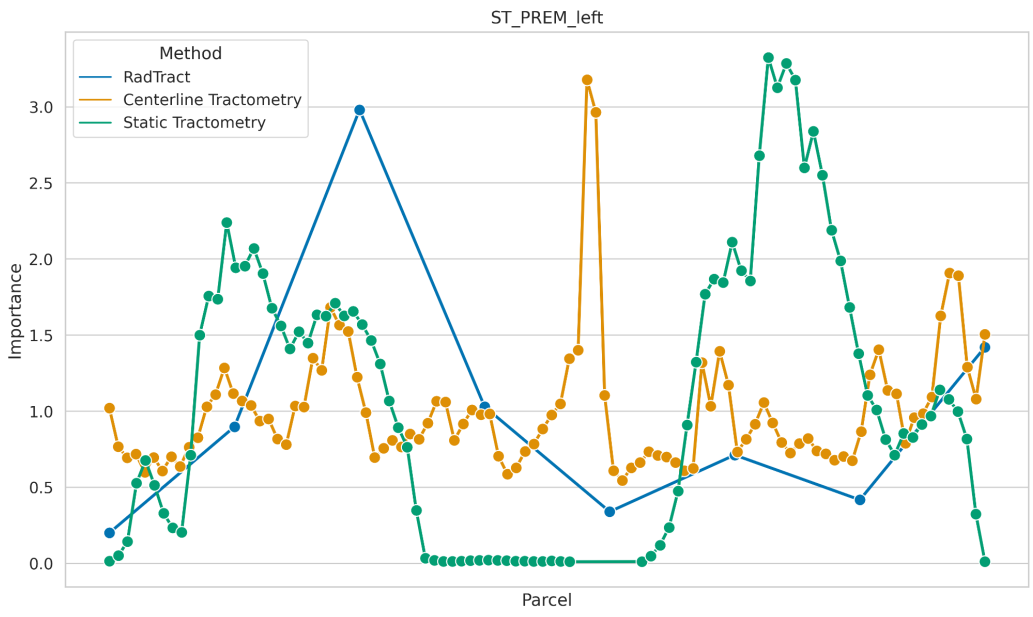

Supplementary Figure 60. Parcel importances for all three methods along the tract (CAT, ST_PREM_left), aggregated over features.

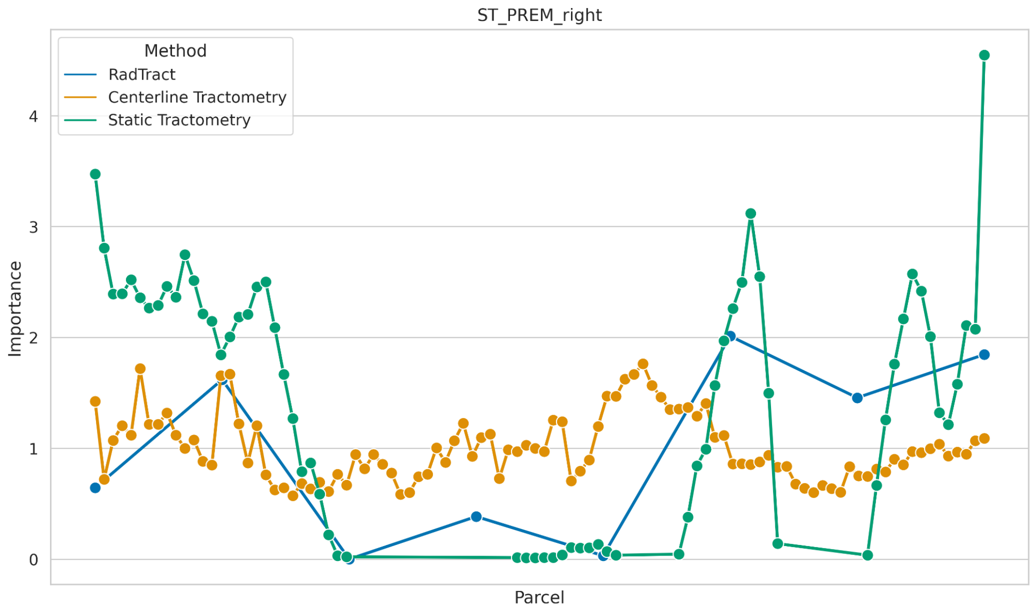

Supplementary Figure 61. Parcel importances for all three methods along the tract (CAT, ST_PREM_right), aggregated over features.

ADNI

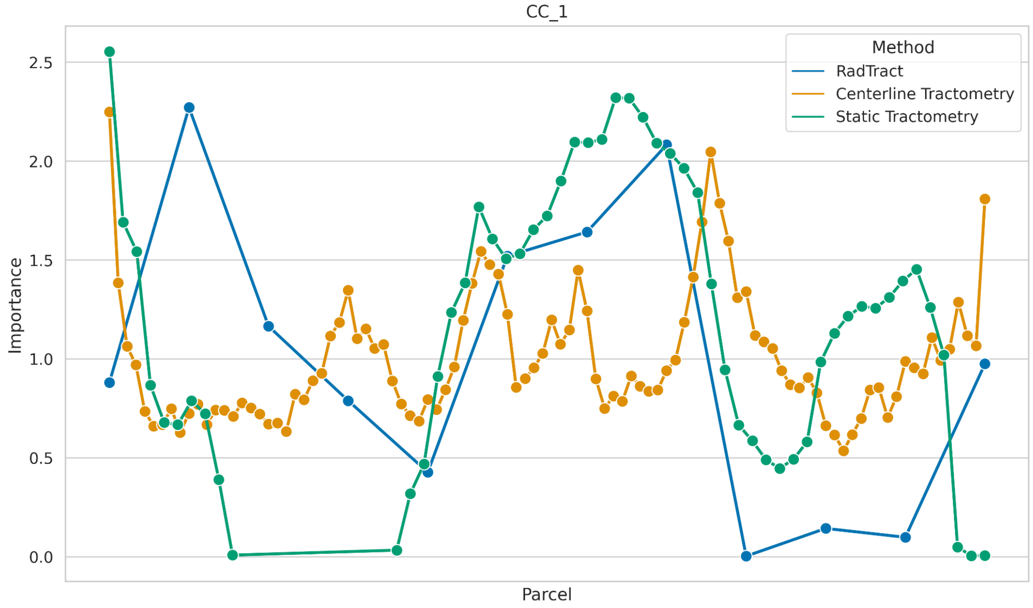

Supplementary Figure 62. Parcel importances for all three methods along the tract (ADNI, CC_1), aggregated over features.

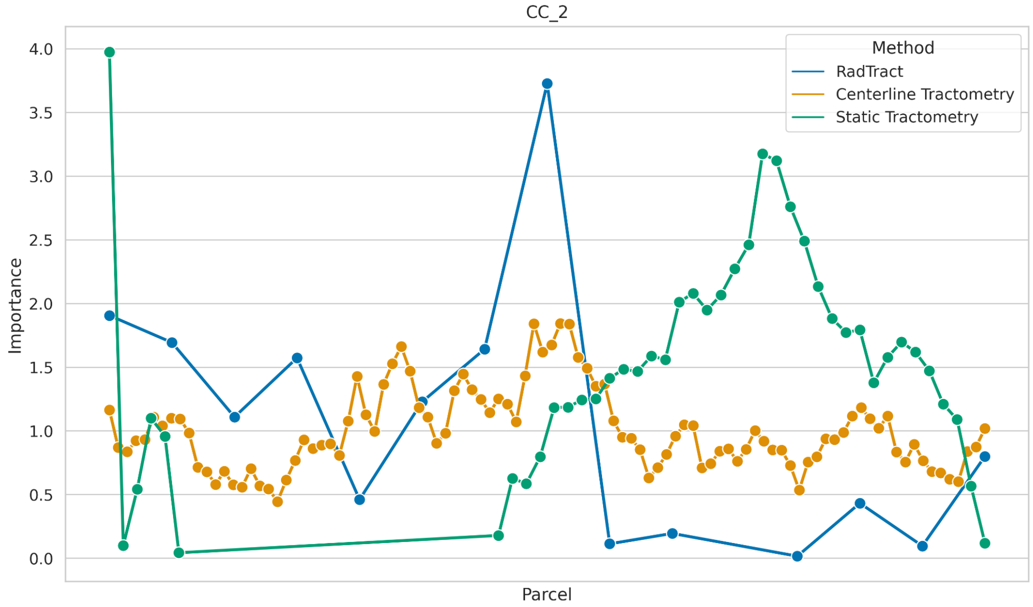

Supplementary Figure 63. Parcel importances for all three methods along the tract (ADNI, CC_2), aggregated over features.

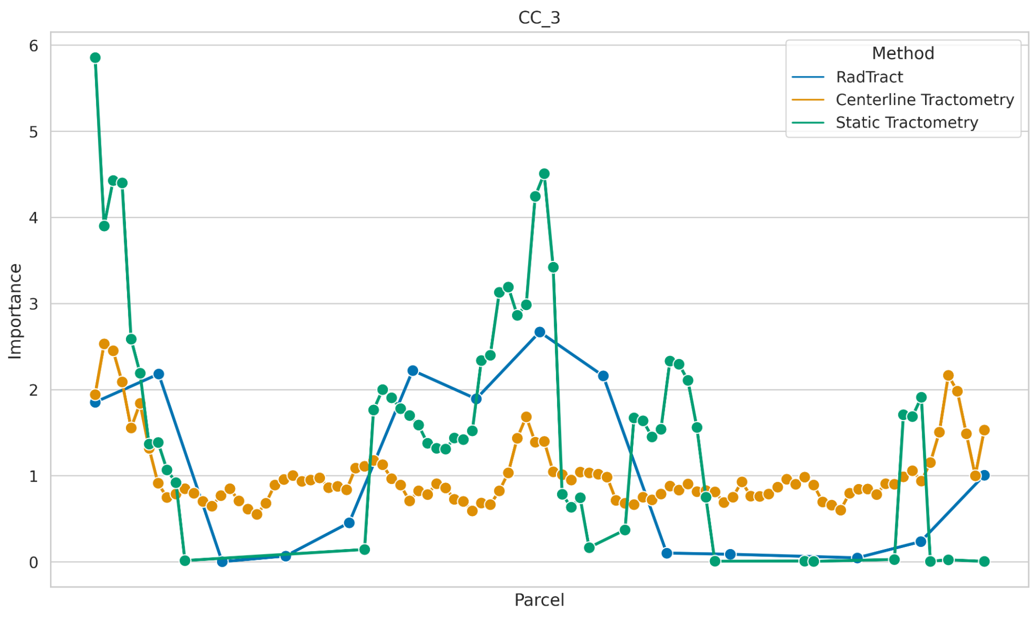

Supplementary Figure 64. Parcel importances for all three methods along the tract (ADNI, CC_3), aggregated over features.

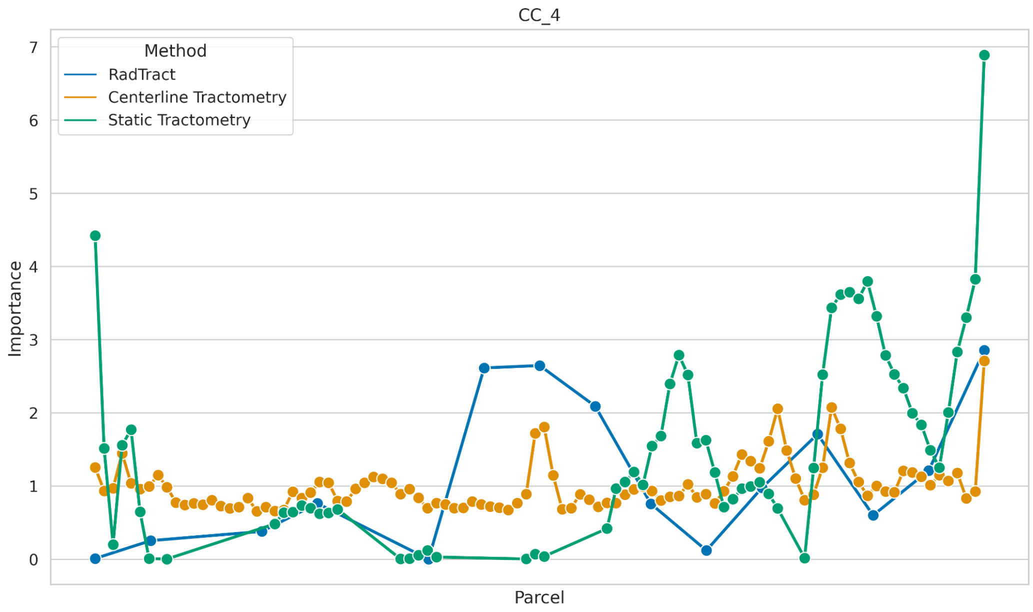

Supplementary Figure 65. Parcel importances for all three methods along the tract (ADNI, CC_4), aggregated over features.

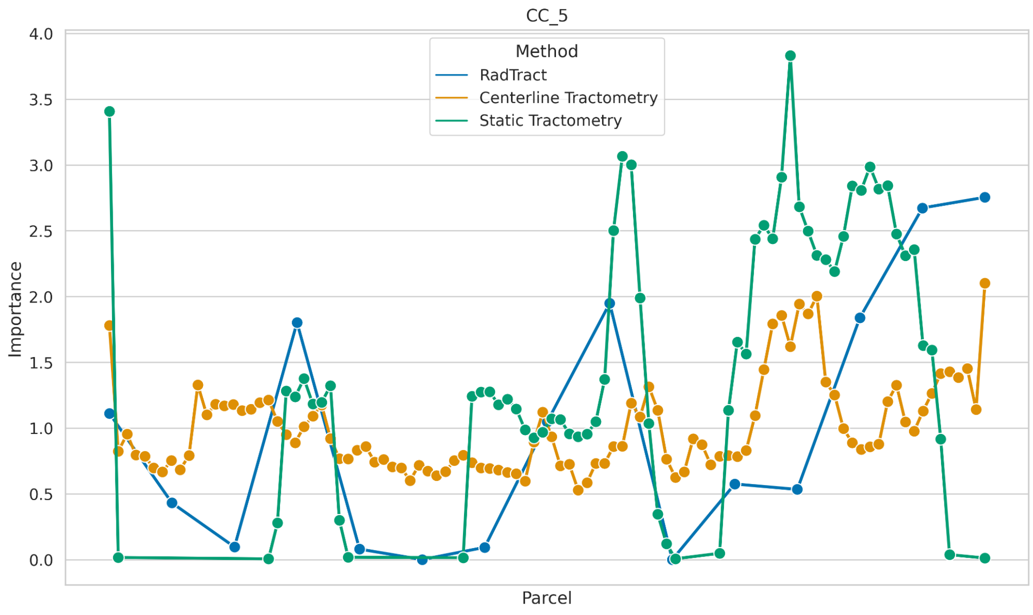

Supplementary Figure 66. Parcel importances for all three methods along the tract (ADNI, CC_5), aggregated over features.

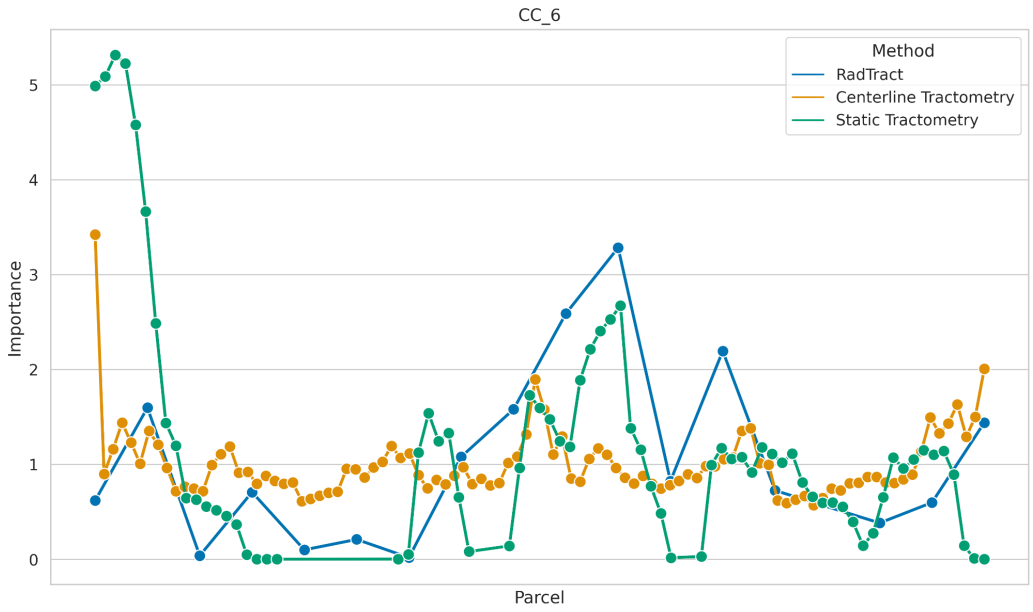

Supplementary Figure 67. Parcel importances for all three methods along the tract (ADNI, CC_6), aggregated over features.

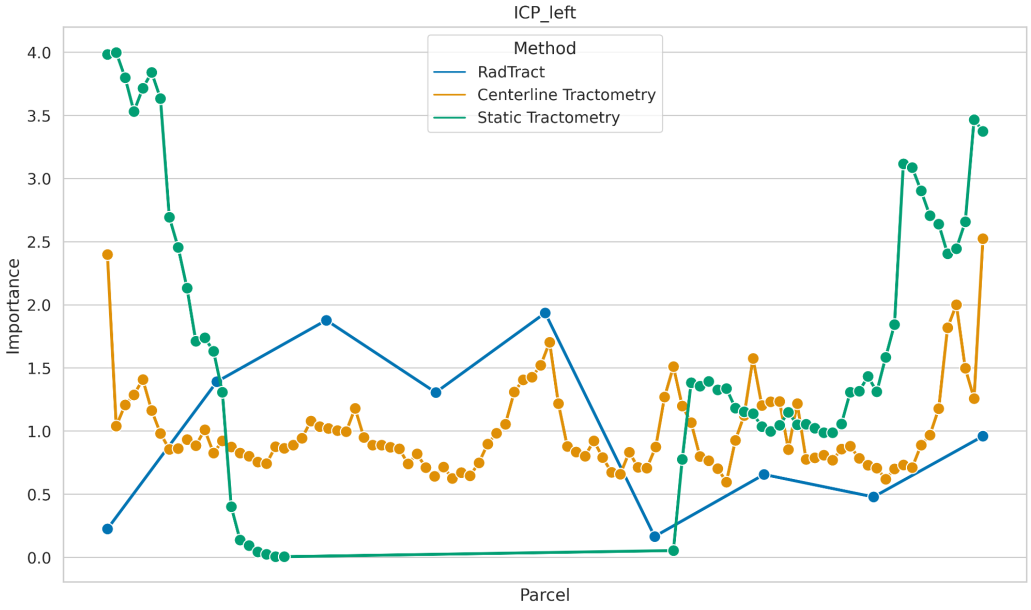

Supplementary Figure 68. Parcel importances for all three methods along the tract (ADNI, ICP_left), aggregated over features.

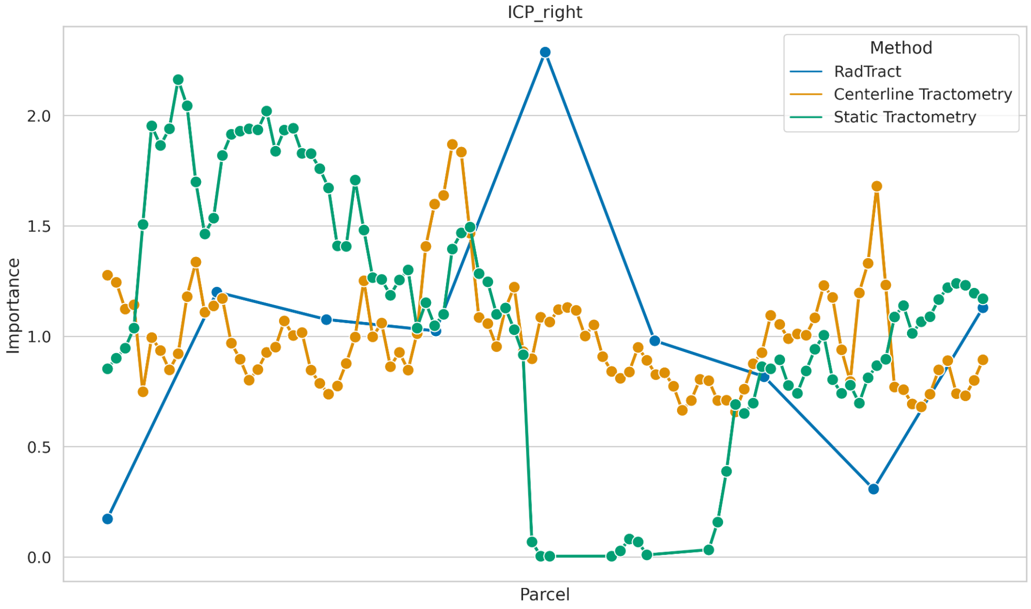

Supplementary Figure 69. Parcel importances for all three methods along the tract (ADNI, ICP_right), aggregated over features.

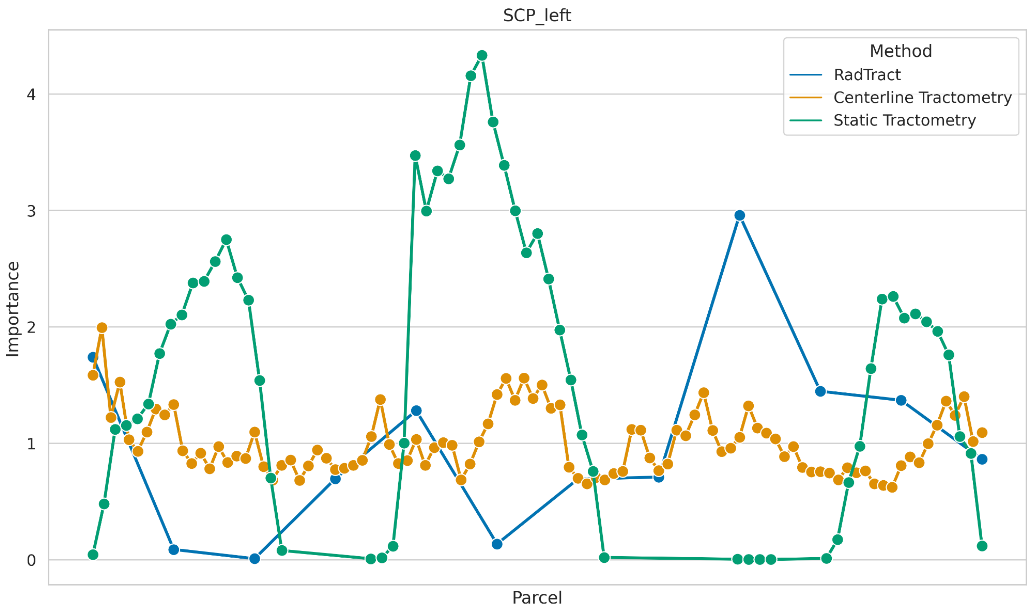

Supplementary Figure 70. Parcel importances for all three methods along the tract (ADNI, SCP_left), aggregated over features.

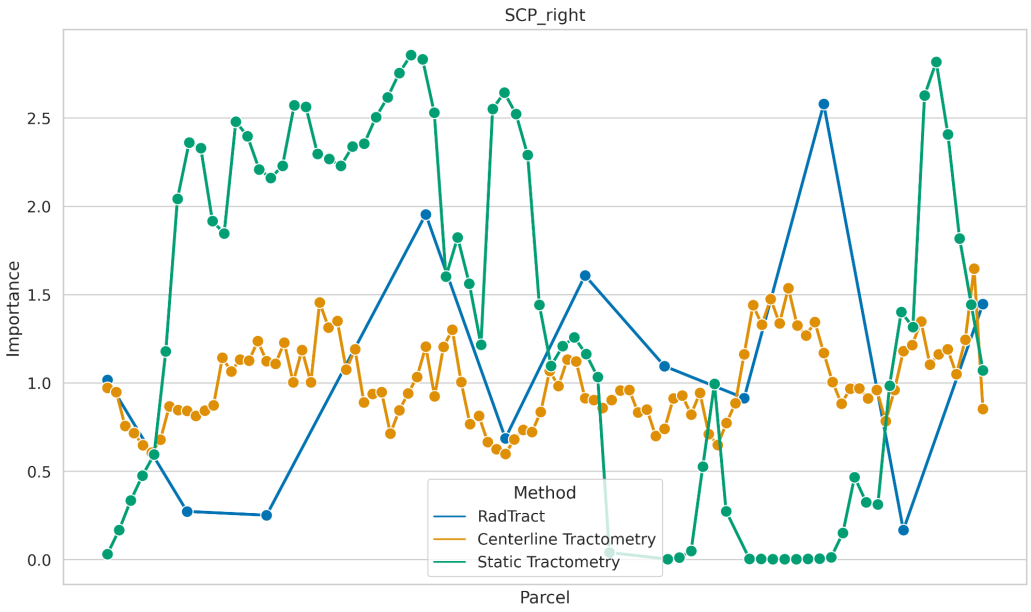

Supplementary Figure 71. Parcel importances for all three methods along the tract (ADNI, SCP_right), aggregated over features.

PPMI

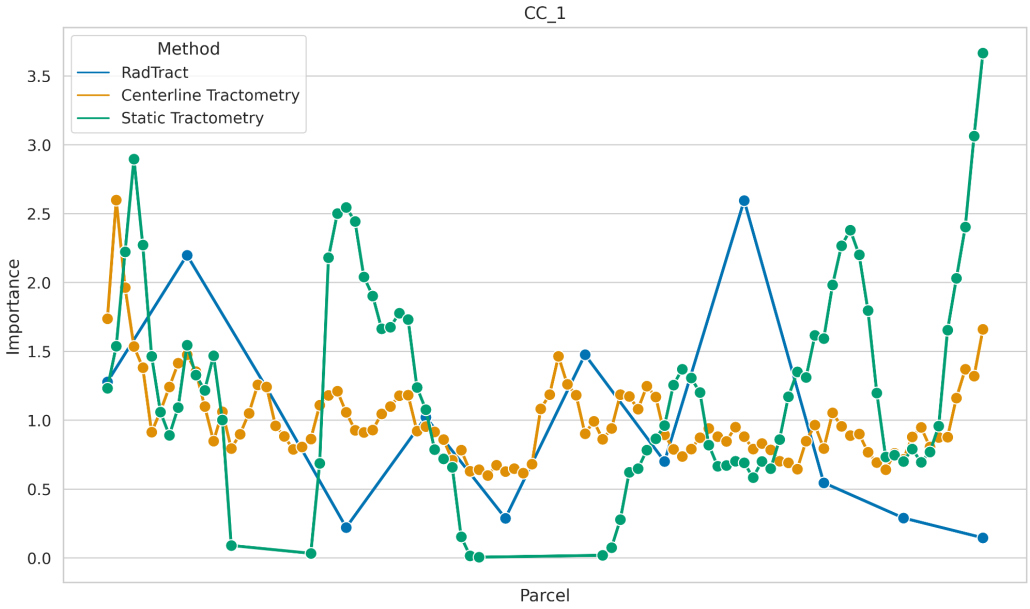

Supplementary Figure 72. Parcel importances for all three methods along the tract (PPMI, CC_1), aggregated over features.

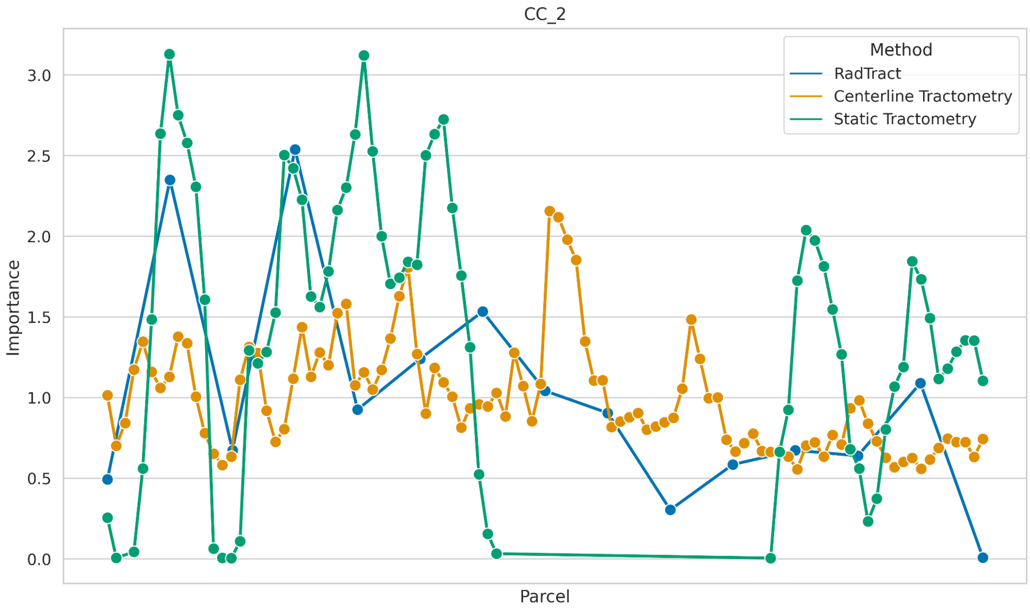

Supplementary Figure 73. Parcel importances for all three methods along the tract (PPMI, CC_2), aggregated over features.

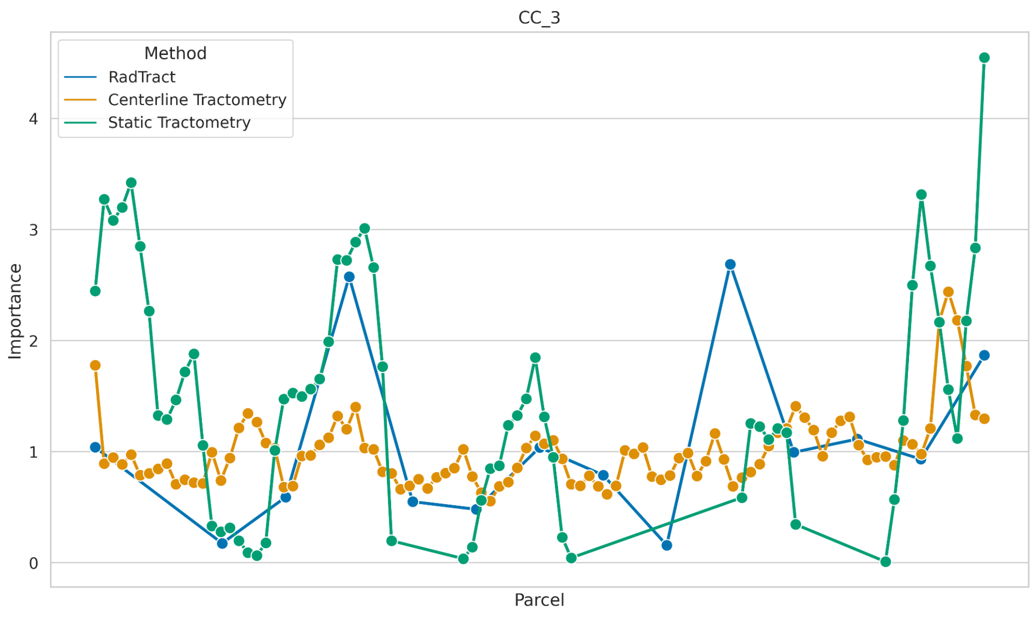

Supplementary Figure 74. Parcel importances for all three methods along the tract (PPMI, CC_3), aggregated over features.

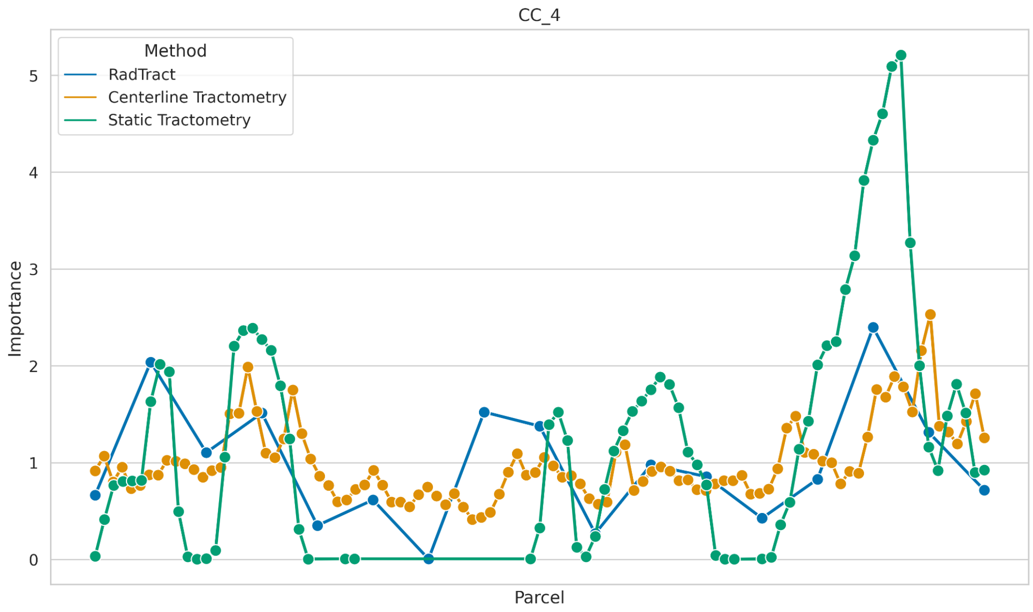

Supplementary Figure 75. Parcel importances for all three methods along the tract (PPMI, CC_4), aggregated over features.

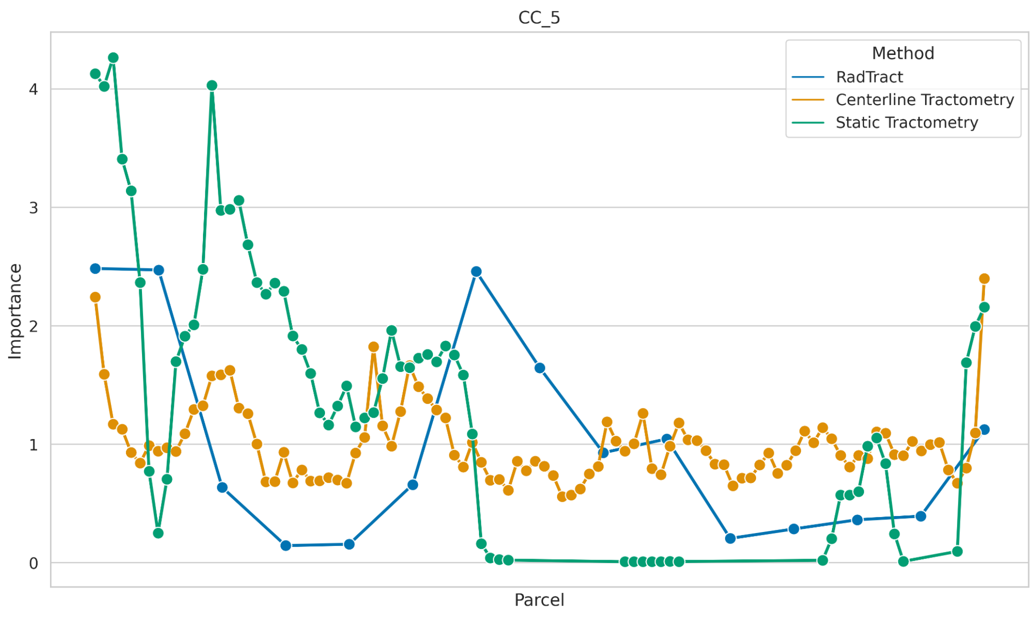

Supplementary Figure 76. Parcel importances for all three methods along the tract (PPMI, CC_5), aggregated over features.

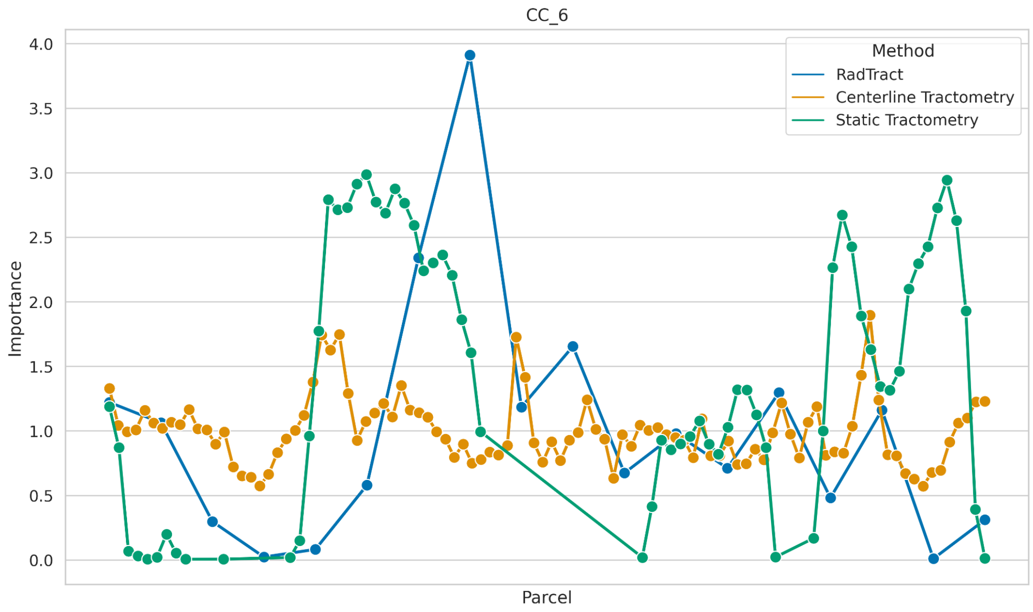

Supplementary Figure 77. Parcel importances for all three methods along the tract (PPMI, CC_6), aggregated over features.

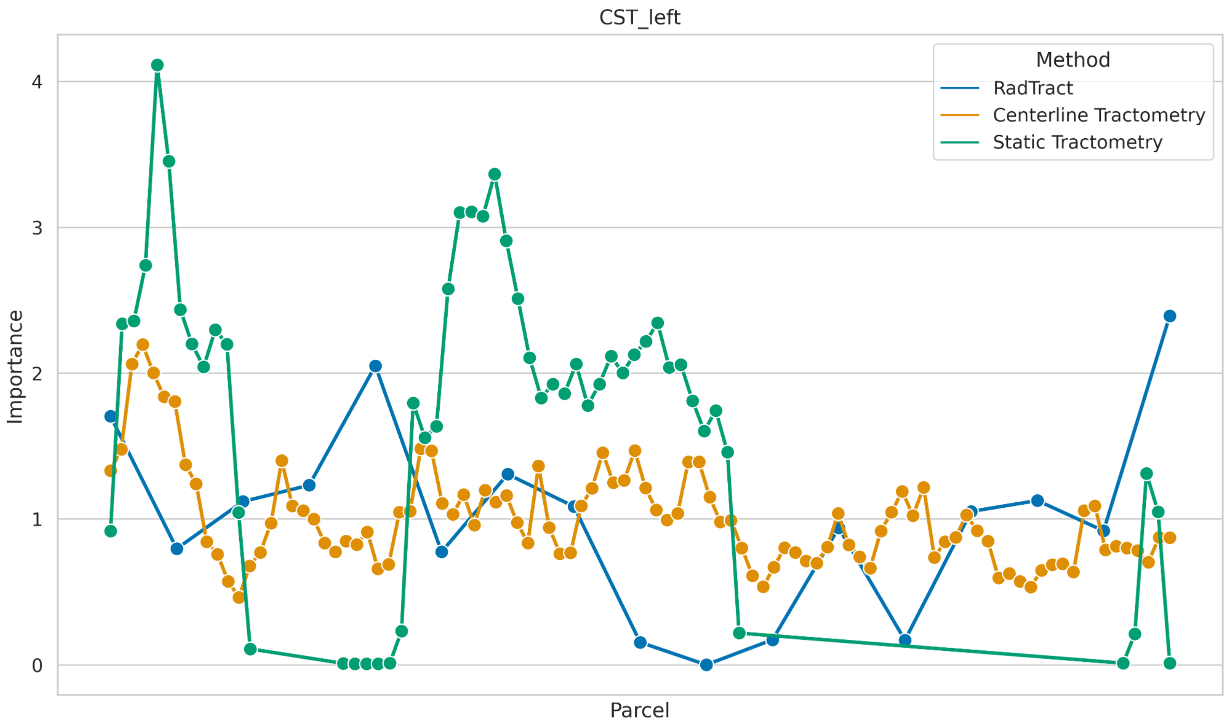

Supplementary Figure 78. Parcel importances for all three methods along the tract (PPMI, CST_left), aggregated over features.

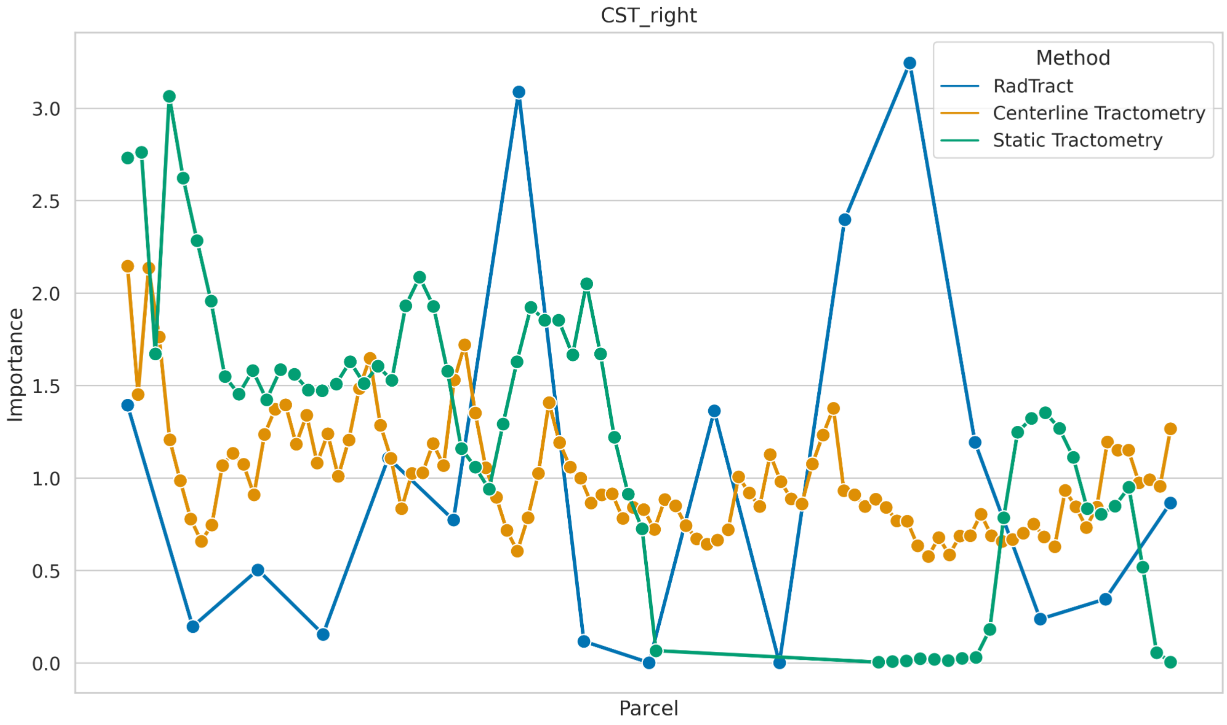

Supplementary Figure 79. Parcel importances for all three methods along the tract (PPMI, CST_right), aggregated over features.

Supplementary Note 6 – Subject IDs

Supplementary Table 7. A complete list of all subject IDs from the openly available ADNI, PPMI, and SCHZ datasets used in the automatic diagnosis experiments.

ADNI	PPMI	SCHZ
027_S_6648_I1108065	3178_I315641	sub-50004
070_S_6911_I1417075	42240_I757826	sub-10448
135_S_6104_I1512341	3104_I226395	sub-50005
127_S_5056_I353205	3780_I337754	sub-10193
013_S_6970_I1469367	60036_I436440	sub-50006
029_S_4279_I356049	3106_I235248	sub-10912
027_S_4962_I354311	3752_I353408	sub-50007
168_S_6634_I1081480	41283_I643379	sub-10524
168_S_6413_I1346145	3109_I232176	sub-50008
127_S_5058_I354635	50485_I665300	sub-10290
016_S_7002_I1493842	75418_I1362240	sub-50010
029_S_4384_I273990	3114_I253902	sub-10871
027_S_4802_I354526	3176_I305943	sub-50013
036_S_6099_I922791	74940_I1199295	sub-10316
020_S_6185_I1117161	3115_I253992	sub-50014
168_S_6828_I1398880	3124_I337673	sub-10235
070_S_6236_I968286	73616_I1224368	sub-50015
032_S_4429_I944321	3151_I243568	sub-10530
168_S_6843_I1275425	3863_I340681	sub-50016
041_S_6731_I1350259	50143_I482348	sub-10189
168_S_6131_I951189	3157_I243695	sub-50020
027_S_4964_I355392	3184_I351036	sub-10345
127_S_6512_I1349365	74375_I1125043	sub-50021
003_S_4350_I1252856	3165_I243772	sub-10271
027_S_4801_I355549	3808_I269575	sub-50022
141_S_6075_I1256860	75419_I1199467	sub-10321
007_S_4620_I337616	3169_I275543	sub-50023
016_S_5032_I405641	3575_I287980	sub-11062
016_S_6789_I1193761	17608_I473072	sub-50025
168_S_6233_I1348601	3171_I275552	sub-11050
036_S_6179_I957114	41488_I951756	sub-50027
041_S_0679_I1555336	60043_I440307	sub-10159
041_S_6292_I1354017	3172_I282109	sub-50029
052_S_5062_I356499	3757_I269527	sub-10388
037_S_6125_I940679	60044_I440312	sub-50032
016_S_4097_I279162	3188_I367305	sub-10624
116_S_6100_I1120781	3179_I344268	sub-50033

168_S_6467_I1235496	50225_I449718	sub-10274
129_S_4422_I404340	3300_I226408	sub-50034
127_S_4992_I356871	3835_I373037	sub-11097
027_S_6034_I872018	60074_I449728	sub-50035
003_S_6067_I1235543	3389_I367351	sub-10325
127_S_5067_I409420	4082_I357642	sub-50036
013_S_6206_I1471783	60035_I416918	sub-10340
005_S_0602_I925145	3390_I374738	sub-50038
126_S_4494_I281598	3831_I353444	sub-10440
137_S_6685_I1149061	51689_I615320	sub-50043
094_S_4649_I953653	3554_I249265	sub-10460
033_S_6824_I1235095	3552_I374744	sub-50047
099_S_6632_I1401549	41473_I449693	sub-10855
021_S_4421_I404204	3563_I265357	sub-50048
127_S_4940_I393251	4024_I344314	sub-11030
037_S_0377_I1117324	50961_I475698	sub-50049
016_S_4121_I280315	3570_I249288	sub-10934
127_S_6549_I1073648	4083_I357645	sub-50050
168_S_6817_I1236279	41471_I449688	sub-10575
027_S_6183_I959458	3571_I265399	sub-50051
016_S_4591_I338984	3789_I356006	sub-11066
003_S_6432_I1016020	59483_I864979	sub-50052
041_S_6785_I1358537	3572_I287929	sub-10977
005_S_4910_I393741	4037_I373057	sub-50053
098_S_6593_I1066561	50328_I437351	sub-11019
041_S_6192_I959652	3750_I353401	sub-50054
016_S_4963_I339510	3556_I226446	sub-10746
168_S_6821_I1244560	52274_I689645	sub-50055
027_S_6001_I808826	3756_I269523	sub-10877
168_S_6827_I1239545	3182_I334523	sub-50056
002_S_1155_I843517	51302_I507842	sub-10339
137_S_6826_I1242439	3759_I282154	sub-50058
032_S_6602_I1058578	3753_I282128	sub-11131
012_S_6073_I1239425	15761_I449529	sub-50059
168_S_6098_I925579	3765_I288121	sub-10697
098_S_4215_I395507	3778_I322976	sub-50060
027_S_6463_I1026594	52432_I665349	sub-11044
068_S_0473_I932021	3767_I288148	sub-50061
098_S_6601_I1061750	3763_I288074	sub-10292
168_S_6875_I1319227	52362_I713734	sub-50064
029_S_4290_I279354	3768_I301542	sub-10227

032_S_6600_I1061846	3131_I367285	sub-50066
014_S_6765_I1351672	85236_I497277	sub-10704
116_S_6133_I1122110	3769_I301547	sub-50067
127_S_6433_I1351391	3181_I357576	sub-10438
070_S_6229_I969045	60033_I475723	sub-50069
099_S_6038_I1276993	3803_I365111	sub-10882
126_S_4686_I344140	3838_I367370	sub-50075
027_S_6002_I812926	51392_I515262	sub-10975
168_S_6108_I930638	3804_I269554	sub-50076
003_S_6833_I1254315	41282_I554301	sub-11068
033_S_7079_I1586139	71903_I1199433	sub-50077
023_S_1190_I929046	3805_I269559	sub-11112
127_S_5028_I346689	3167_I259598	sub-50080
135_S_6446_I1012945	50451_I466967	sub-10707
035_S_6160_I950893	3806_I269564	sub-50081
114_S_6595_I1078732	3154_I243604	sub-11149
003_S_6258_I973301	75414_I1130193	sub-50083
016_S_6790_I1359943	3807_I269571	sub-11098
029_S_4307_I267604	3574_I287960	sub-50085
021_S_0178_I1432271	50172_I466960	sub-10455
029_S_4652_I350104	3812_I269595	
016_S_4353_I267938	4013_I344311	
137_S_6906_I1454390	60073_I440322	
070_S_6191_I958925	3813_I275574	
016_S_6839_I1263266	3111_I243547	
027_S_6370_I1018309	16644_I482291	
016_S_4952_I353273	3816_I340641	
135_S_6840_I1263427	4012_I344306	
014_S_6087_I926924	75512_I1199326	
003_S_4441_I277112	3850_I238035	
005_S_4707_I351694	3327_I362476	
033_S_6889_I1508480	72138_I1003473	
168_S_6371_I1352201	3851_I395238	
003_S_4373_I274608	3127_I337704	
109_S_6215_I972973	51680_I1044925	
024_S_6184_I958736	3855_I243827	
168_S_6142_I946990	3567_I249275	
941_S_6052_I876893	74000_I1199274	
011_S_0021_I957753	4004_I275713	
016_S_4009_I273932	53060_I831139	
052_S_1352_I487156	60075_I467057	

033_S_4176_I1117704	4032_I367384	
098_S_4201_I282897	3122_I305873	
114_S_6597_I1080323	60091_I507854	
168_S_6107_I930695	4067_I356065	
127_S_4500_I283516	4135_I377950	
168_S_6908_I1416091	85242_I482360	
041_S_6786_I1359843	4085_I377943	
126_S_6683_I1129348	3828_I340677	
016_S_6904_I1412978	60006_I554332	
027_S_6582_I1057808	4139_I377980	
003_S_4152_I287952		
109_S_6406_I1028364		
041_S_6136_I944430		
027_S_6849_I1287078		
126_S_7060_I1573349		
098_S_4275_I601142		
016_S_5057_I1413015		
009_S_6402_I1017806		
109_S_4499_I338053		
127_S_5095_I361475		
137_S_6880_I1360099		
021_S_6896_I1395576		
007_S_4568_I287485		
052_S_0671_I501928		
021_S_4558_I337418		
052_S_4959_I361968		
137_S_6693_I1417029		
098_S_4050_I262869		
135_S_6687_I1142638		
022_S_6280_I983060		
099_S_4076_I920986		
033_S_6705_I1147608		
168_S_6860_I1300012		
127_S_4604_I1500932		
005_S_5119_I364834		
014_S_6944_I1450072		
007_S_4387_I355628		
168_S_6921_I1428772		
012_S_6503_I1052054		
041_S_4427_I915921		
033_S_7066_I1570697		

127_S_1427_I1059963		
301_S_6224_I1387628		
126_S_6721_I1157969		
941_S_6068_I893051		
041_S_6159_I949932		
022_S_6013_I844189		
037_S_6141_I952054		
003_S_4900_I379027		
003_S_5165_I373248		
137_S_6557_I1043644		
116_S_4453_I1539922		
168_S_6938_I1444126		
016_S_6809_I1236028		
016_S_6381_I1359928		
127_S_4749_I307104		
109_S_6373_I1073429		
003_S_4288_I915007		
007_S_5196_I376233		
099_S_6691_I1365258		
098_S_4003_I261446		
003_S_6264_I1005746		
027_S_6643_I1283749		
168_S_6151_I947554		
003_S_5187_I375948		
003_S_6479_I1246028		
005_S_6093_I924231		
168_S_6735_I1175371		
141_S_6964_I1466176		
021_S_4276_I529855		
094_S_4737_I309922		
135_S_6110_I1256455		
127_S_4843_I343540		
033_S_6976_I1463014		
116_S_6428_I1181056		
027_S_6577_I1384718		
129_S_6763_I1186912		
168_S_6426_I1241424		
016_S_6834_I1256816		
129_S_6784_I1194008		
037_S_0150_I1117308		
033_S_0734_I1058042		

016_S_5251_I385049		
036_S_6134_I942641		
016_S_6892_I1373765		
003_S_4142_I254902		
016_S_6800_I1426463		
033_S_1016_I1262206		
011_S_4827_I898889		
022_S_6716_I1162717		
041_S_6314_I1371447		
021_S_4924_I331255		
109_S_6221_I1022947		
035_S_6156_I948834		
135_S_7003_I1494221		
003_S_6678_I1119581		
033_S_1098_I941822		

Supplementary Note 7 – Commands and parameters

Supplementary Table 8. A complete list of the commands and parameters used for preprocessing the data.

Processing Step	Tool	Command
Denoising	`MRtrix`	`dwidenoise dwi.nii.gz dwi_denoise.nii.gz`
Gibbs ringing removal	`MRtrix`	`mrdegibbs dwi_denoise.nii.gz dwi_denoise_degibbs.nii.gz`
Eddy current and inhomogeneity distortion correction	`MRtrix`	`dwifslpreproc dwi_denoise_degibbs.nii.gz dwi_denoise_degibbs_fslpreproc.nii.gz -fslgrad dwi.bvecs dwi.bvals -rpe_none -pe_dir PA -export_grad_fsl dwi_denoise_degibbs_fslpreproc.bvecs dwi_denoise_degibbs_fslpreproc.bvals`
Bias field correction	`MRtrix`	`dwibiascorrect fsl dwi_denoise_degibbs_fslpreproc.nii.gz dwi_denoise_degibbs_fslpreproc_biascorrect.nii.gz -bias estimated_biasfield.nii.gz -fslgrad dwi_denoise_degibbs_fslpreproc.bvecs dwi_denoise_degibbs_fslpreproc.bvals`
MNI registration	`MITK Diffusion`	`MitkDReg --version -f MNI_FA_template.nii.gz -m dwi_denoise_degibbs_fslpreproc_biascorrect.nii.gz -o Diffusion_MNI.nii.gz --resample`
Brain mask estimation	`FSL`	`bet Diffusion_MNI.nii.gz bet_brain.nii.gz -f 0.1 -m`

Tensor calculation	MRtrix	dwi2tensor Diffusion_MNI.nii.gz Diffusion_MNI_tensors.nii.gz -fslgrad Diffusion_MNI.bvecs Diffusion_MNI.bvals -mask bet_brain_mask.nii.gz
FA map calculation	MRtrix	tensor2metric Diffusion_MNI_tensors.nii.gz -fa Diffusion_MNI_tensors_fa.nii.gz -mask bet_brain_mask.nii.gz
Response function calculation	MRtrix	dwi2response tournier Diffusion_MNI.nii.gz response.txt -mask bet_brain_mask.nii.gz -fslgrad Diffusion_MNI.bvecs Diffusion_MNI.bvals
Constrained spherical deconvolution	MRtrix	dwi2fod csd Diffusion_MNI.nii.gz response.txt WM_FODs.nii.gz -mask bet_brain_mask.nii.gz -fslgrad Diffusion_MNI.bvecs Diffusion_MNI.bvals
Peak calculation	MRtrix	sh2peaks WM_FODs.nii.gz peaks.nii.gz
Peak flip (only for ADNI and PPMI)	TractSeg	flip_peaks -i peaks.nii.gz -o peaks.nii.gz -a x
TractSeg tract segmentation	TractSeg	TractSeg -i peaks.nii.gz -o tractseg_output/ --verbose --keep_intermediate_files
TractSeg endpoint segmentation	TractSeg	TractSeg -i peaks.nii.gz -o tractseg_output/ --verbose --keep_intermediate_files --output_type endings_segmentation
Tractseg TOM calculation	TractSeg	TractSeg -i peaks.nii.gz -o tractseg_output/ --verbose --keep_intermediate_files --output_type TOM
Tractography of CC	TractSeg	Tracking -i peaks.nii.gz -o tractseg_output/ --tracking_format trk --nr_fibers 30000 --bundles CC
Tractography of all other tracts	TractSeg	Tracking -i peaks.nii.gz -o tractseg_output/ --tracking_format trk --nr_fibers 10000 --bundles BUNDLE_NAME

Supplementary Note 8 – CC_7 tractography issues

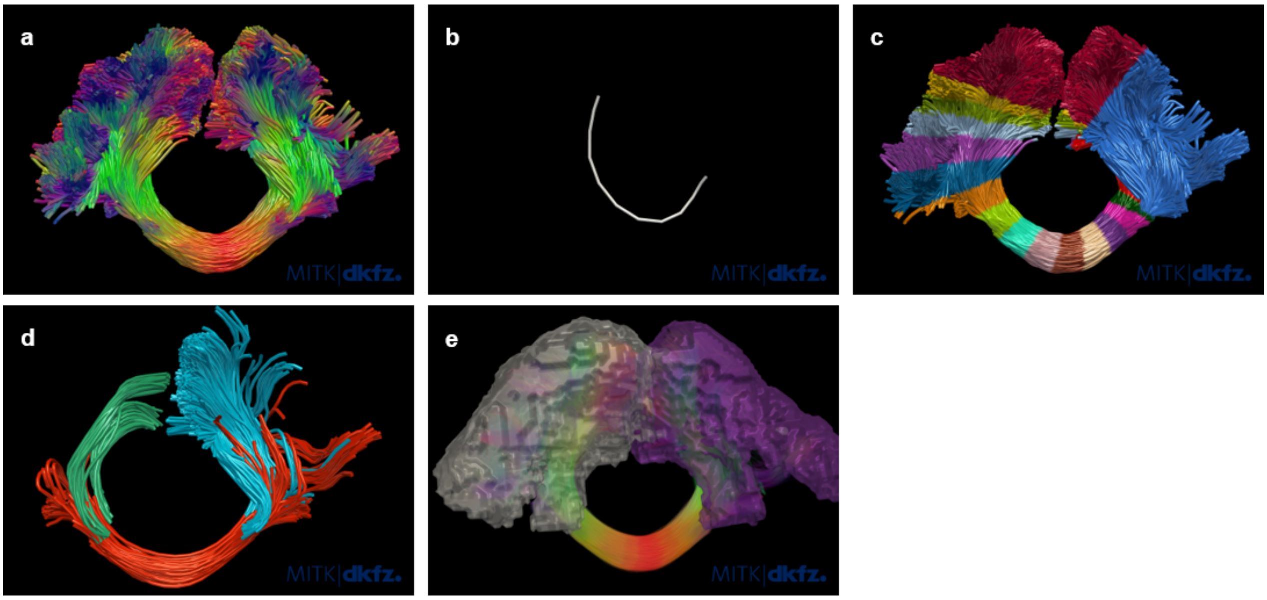

Supplementary Figure 80. Illustration of the adverse effects of tractography errors on the subsequent parcellation, independent of the used parcellation method. **a** The original tract, which seems fine. **b** The resulting centroid on the other hand seems truncated. **c** The parcellation shows severe errors. **d** These errors are caused by broken subparts of the tractogram, which end in wrong regions. **e** These errors are again caused by a faulty segmentation of the tract's start- and end-regions, which here are frayed and partly overlapping.

Supplementary Note 9 – Pyradiomics features

Supplementary Table 9. Complete list of all features calculated per parcel. For implementation details, please refer to https://pyradiomics.readthedocs.io/en/v3.0.1/

Nr.	Feature Name
1	original_shape_Elongation
2	original_shape_Flatness
3	original_shape_LeastAxisLength
4	original_shape_MajorAxisLength
5	original_shape_Maximum2DDiameterColumn
6	original_shape_Maximum2DDiameterRow
7	original_shape_Maximum2DDiameterSlice
8	original_shape_Maximum3DDiameter
9	original_shape_MeshVolume
10	original_shape_MinorAxisLength
11	original_shape_Sphericity
12	original_shape_SurfaceArea
13	original_shape_SurfaceVolumeRatio
14	original_shape_VoxelVolume
15	original_firstorder_10Percentile
16	original_firstorder_90Percentile

17	original_firstorder_Energy
18	original_firstorder_Entropy
19	original_firstorder_InterquartileRange
20	original_firstorder_Kurtosis
21	original_firstorder_Maximum
22	original_firstorder_MeanAbsoluteDeviation
23	original_firstorder_Mean
24	original_firstorder_Median
25	original_firstorder_Minimum
26	original_firstorder_Range
27	original_firstorder_RobustMeanAbsoluteDeviation
28	original_firstorder_RootMeanSquared
29	original_firstorder_Skewness
30	original_firstorder_TotalEnergy
31	original_firstorder_Uniformity
32	original_firstorder_Variance
33	original_glcm_Autocorrelation
34	original_glcm_JointAverage
35	original_glcm_ClusterProminence
36	original_glcm_ClusterShade
37	original_glcm_ClusterTendency
38	original_glcm_Contrast
39	original_glcm_Correlation
40	original_glcm_DifferenceAverage
41	original_glcm_DifferenceEntropy
42	original_glcm_DifferenceVariance
43	original_glcm_JointEnergy
44	original_glcm_JointEntropy
45	original_glcm_Imc1
46	original_glcm_Imc2
47	original_glcm_Idm
48	original_glcm_Idmn
49	original_glcm_Id
50	original_glcm_Idn
51	original_glcm_InverseVariance
52	original_glcm_MaximumProbability
53	original_glcm_SumEntropy
54	original_glcm_SumSquares

55	original_glrlm_GrayLevelNonUniformity
56	original_glrlm_GrayLevelNonUniformityNormalized
57	original_glrlm_GrayLevelVariance
58	original_glrlm_HighGrayLevelRunEmphasis
59	original_glrlm_LongRunEmphasis
60	original_glrlm_LongRunHighGrayLevelEmphasis
61	original_glrlm_LongRunLowGrayLevelEmphasis
62	original_glrlm_LowGrayLevelRunEmphasis
63	original_glrlm_RunEntropy
64	original_glrlm_RunLengthNonUniformity
65	original_glrlm_RunLengthNonUniformityNormalized
66	original_glrlm_RunPercentage
67	original_glrlm_RunVariance
68	original_glrlm_ShortRunEmphasis
69	original_glrlm_ShortRunHighGrayLevelEmphasis
70	original_glrlm_ShortRunLowGrayLevelEmphasis
71	original_glszm_GrayLevelNonUniformity
72	original_glszm_GrayLevelNonUniformityNormalized
73	original_glszm_GrayLevelVariance
74	original_glszm_HighGrayLevelZoneEmphasis
75	original_glszm_LargeAreaEmphasis
76	original_glszm_LargeAreaHighGrayLevelEmphasis
77	original_glszm_LargeAreaLowGrayLevelEmphasis
78	original_glszm_LowGrayLevelZoneEmphasis
79	original_glszm_SizeZoneNonUniformity
80	original_glszm_SizeZoneNonUniformityNormalized
81	original_glszm_SmallAreaEmphasis
82	original_glszm_SmallAreaHighGrayLevelEmphasis
83	original_glszm_SmallAreaLowGrayLevelEmphasis
84	original_glszm_ZoneEntropy
85	original_glszm_ZonePercentage
86	original_glszm_ZoneVariance
87	original_gldm_DependenceEntropy
88	original_gldm_DependenceNonUniformity
89	original_gldm_DependenceNonUniformityNormalized
90	original_gldm_DependenceVariance
91	original_gldm_GrayLevelNonUniformity
92	original_gldm_GrayLevelVariance

93	original_gldm_HighGrayLevelEmphasis
94	original_gldm_LargeDependenceEmphasis
95	original_gldm_LargeDependenceHighGrayLevelEmphasis
96	original_gldm_LargeDependenceLowGrayLevelEmphasis
97	original_gldm_LowGrayLevelEmphasis
98	original_gldm_SmallDependenceEmphasis
99	original_gldm_SmallDependenceHighGrayLevelEmphasis
100	original_gldm_SmallDependenceLowGrayLevelEmphasis
101	original_ngtdm_Busyness
102	original_ngtdm_Coarseness
103	original_ngtdm_Complexity
104	original_ngtdm_Contrast
105	original_ngtdm_Strength

Supplementary Note 10 – Age and Sex Statistics

Supplementary Table 10. Age and sex statistics for all datasets and subjects used for the classification tasks.

Dataset	Age per class	Sex per class
SCHZ	Class 1: 36.2 +/- 8.8<br>Class 2: 36.1 +/- 8.8	Class 1: 37M / 12F<br>Class 2: 37M / 12F
CAT	Class 1: 39.4 +/- 10.3<br>Class 1: 38.0 +/- 11.1<br>Class 3: 35.14 +/- 14.18	Class 1: 16M / 14F<br>Class 2: 14M / 15F<br>Class 3: 20M / 8F
ADNI	Class 1: 74.6 +/- 8.5<br>Class 2: 74.9 +/- 8.1<br>Class 3: 74.4 +/- 8.4	Class 1: 39M / 33F<br>Class 2: 39M / 33F<br>Class 3: 39M / 33F
PPMI	Class 1: 62.6 +/- 7.6<br>Class 2: 62.6 +/- 7.6<br>Class 3: 62.6 +/- 7.8	Class 1: 33M / 20F<br>Class 2: 33M / 20F<br>Class 3: 33M / 20F
