# Supplementary material for: Radiomic tractometry reveals tract-specific imaging biomarkers in white matter
Source: Nat Commun. 2024 Jan 5;15:303. doi: 10.1038/s41467-023-44591-3 (PMC10770385; doi:10.1038/s41467-023-44591-3)
Supplement: Supplementary file 3 — Description of Additional Supplementary Files [file 41467_2023_44591_MOESM3_ESM.pdf]

Description of Additional Supplementary Files

Title: Supplementary Software

Description: Radtract code
