# Supplementary figures and images for: Radiomic tractometry reveals tract-specific imaging biomarkers in white matter
Source: Nat Commun. 2024 Jan 5;15:303. doi: 10.1038/s41467-023-44591-3 (PMC10770385; doi:10.1038/s41467-023-44591-3)

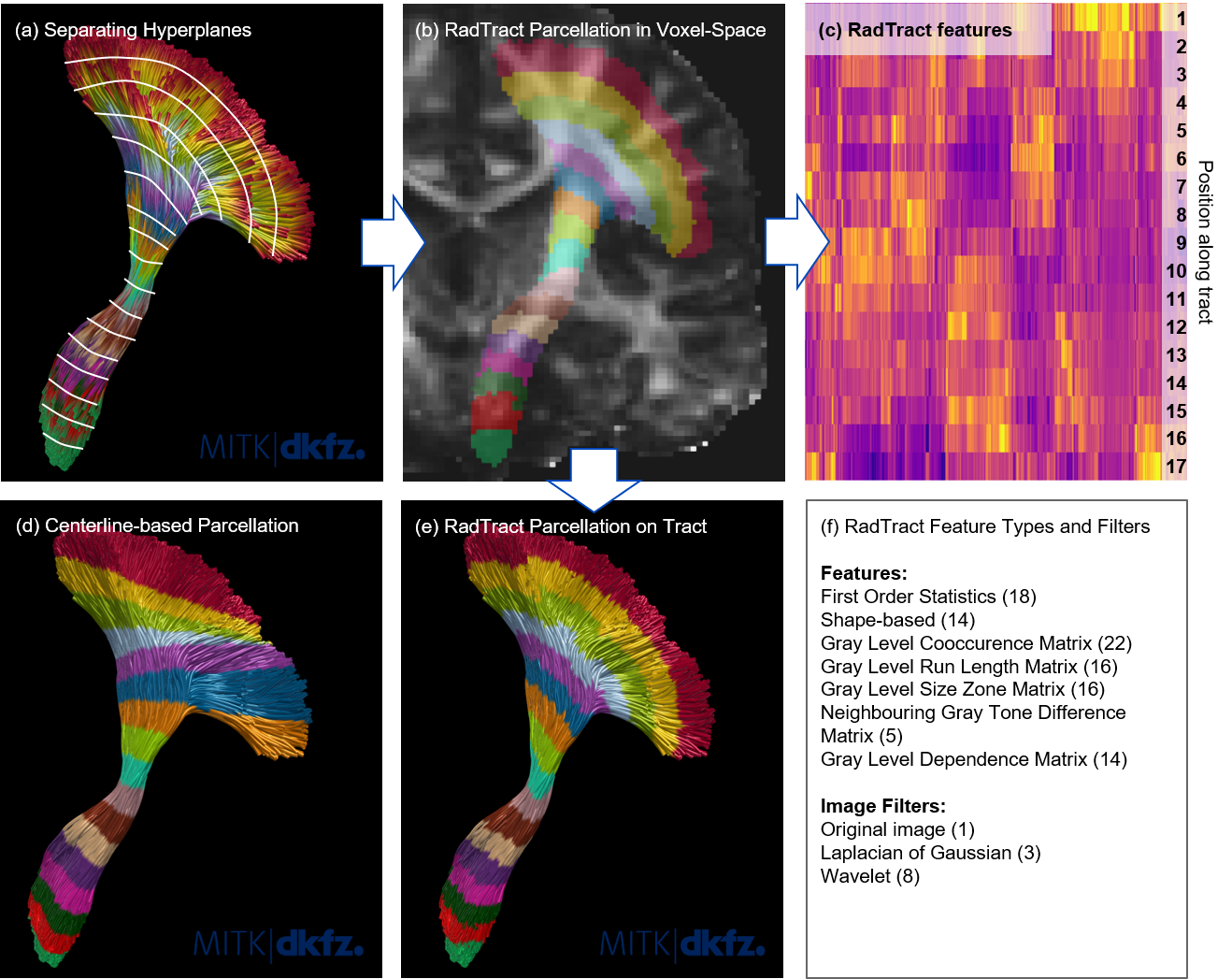

Supplement: Supplementary file 4 — Supplementary Software [file 41467_2023_44591_MOESM4_ESM.zip › radtract-main/resources/radtract_overview.png]
