# Supplementary material for: Radiomic tractometry reveals tract-specific imaging biomarkers in white matter
Source: Nat Commun. 2024 Jan 5;15:303. doi: 10.1038/s41467-023-44591-3 (PMC10770385; doi:10.1038/s41467-023-44591-3)
Supplement: Supplementary file 5 — Reporting Summary [file 41467_2023_44591_MOESM5_ESM.pdf]

nature portfolio

Corresponding author(s): Peter Neher

Last updated by author(s): Dec 6, 2023

Reporting Summary

Nature Portfolio wishes to improve the reproducibility of the work that we publish. This form provides structure for consistency and transparency in reporting. For further information on Nature Portfolio policies, see our Editorial Policies and the Editorial Policy Checklist.

Statistics

For all statistical analyses, confirm that the following items are present in the figure legend, table legend, main text, or Methods section.

n/a	Confirmed	
☐	☒	The exact sample size (*n*) for each experimental group/condition, given as a discrete number and unit of measurement
☐	☒	A statement on whether measurements were taken from distinct samples or whether the same sample was measured repeatedly
☐	☒	The statistical test(s) used AND whether they are one- or two-sided<br>*Only common tests should be described solely by name; describe more complex techniques in the Methods section.*
☒	☐	A description of all covariates tested
☐	☒	A description of any assumptions or corrections, such as tests of normality and adjustment for multiple comparisons
☐	☒	A full description of the statistical parameters including central tendency (e.g. means) or other basic estimates (e.g. regression coefficient) AND variation (e.g. standard deviation) or associated estimates of uncertainty (e.g. confidence intervals)
☐	☒	For null hypothesis testing, the test statistic (e.g. *F*, *t*, *r*) with confidence intervals, effect sizes, degrees of freedom and *P* value noted<br>*Give P values as exact values whenever suitable.*
☒	☐	For Bayesian analysis, information on the choice of priors and Markov chain Monte Carlo settings
☒	☐	For hierarchical and complex designs, identification of the appropriate level for tests and full reporting of outcomes
☐	☒	Estimates of effect sizes (e.g. Cohen's *d*, Pearson's *r*), indicating how they were calculated

Our web collection on statistics for biologists contains articles on many of the points above.

Software and code

Policy information about availability of computer code

Data collection	No software was used for data collection
Data analysis	RadTract code is included in the submission and available on https://github.com/mic-dkfz/radtract and https://pypi.org/project/radtract/. We used the support vector classification as well as random forest classification and regression implemented in scikit-learn (v1.1.2) in our implementation of the RadTract parcellation function as well as all classification and regression experiments. Default parameterization was used if not stated otherwise. Pyradiomics v3.0.1 was used for all radiomics feature calculations. Further used python packages include numpy (v1.23.3), scipy (v1.9.1), pydicom (v2.3.0), nibabel (v4.0.2), skimage (v0.19.3), dipy (v1.5.0), TractSeg (v2.7) and vtk (v9.2.0). Python version 3.10 was used in all experiments.

For manuscripts utilizing custom algorithms or software that are central to the research but not yet described in published literature, software must be made available to editors and reviewers. We strongly encourage code deposition in a community repository (e.g. GitHub). See the Nature Portfolio guidelines for submitting code & software for further information.

Data

Policy information about availability of data

All manuscripts must include a data availability statement. This statement should provide the following information, where applicable:

- Accession codes, unique identifiers, or web links for publicly available datasets
- A description of any restrictions on data availability
- For clinical datasets or third party data, please ensure that the statement adheres to our policy

No new data was collected in this work. Four datasets were included in the presented study:

1. The ADNI data used in this study are available in the Image and Data Archive (IDA, https://ida.loni.usc.edu/). Access can be obtained via https://adni.loni.usc.edu/data-samples/access-data/.
2. The PPMI data used in this study are available in the Image and Data Archive (IDA, https://ida.loni.usc.edu/). Access can be obtained via https://www.ppmi-info.org/access-data-specimens/download-data.
3. The SCHZ data used in this study are available in the OpenNeuro database under accession code ds000030 https://openneuro.org/datasets/ds000030/versions/00016/download.
4. CAT: a non-public dataset acquired at the Central Institute of Mental Health (CIMH, https://www.zi-mannheim.de/en/). The CAT dataset is not publicly available.

The IDs of all included subjects of the public datasets can be found in Supplementary Table 7 and the corresponding imaging parameters can be accessed via the dataset webpage. Source data are provided with this paper.

Research involving human participants, their data, or biological material

Policy information about studies with human participants or human data. See also policy information about sex, gender (identity/presentation), and sexual orientation and race, ethnicity and racism.

Reporting on sex and gender	Sex was considered in the study design in the form that all groups were matched in this regard. Gender was neither collected nor used in the presented study.
Reporting on race, ethnicity, or other socially relevant groupings	No variables in this category were collected or used in the presented study.
Population characteristics	Population characteristics observed in this study are age, sex, and diagnosis. Groups were matched/Balanced for these variables. In case of the CAT dataset, further analyzes characteristics are:<br>Pack-Years: the number of packs of cigarettes smoked per day by the number of years the person has smoked.<br>Education: the number of years the person spent in an educational institution, such as high school or university.<br>BPRS total: aggregated score on the Brief Psychiatric Rating Scale (BPRS), measuring the severity of various psychiatric symptoms.<br>PANSS total: aggregated score on the Positive and Negative Syndrome Scale (PANSS), measuring symptom severity of patients with schizophrenia.<br>GAF: score on the Global Assessment of Functioning scale, measuring the social, occupational, and psychological functioning of the person.<br>OLZe: indicating the daily doses of antipsychotic medication in terms of Olanzapine equivalents (OLZe).
Recruitment	No new participants were recruited, only retrospective data was used in the presented study.
Ethics oversight	The local Ethics Committee I (Medical Faculty Heidelberg at Heidelberg University, Germany) approved the CAT study. All other data is publicly available.

Note that full information on the approval of the study protocol must also be provided in the manuscript.

Field-specific reporting

Please select the one below that is the best fit for your research. If you are not sure, read the appropriate sections before making your selection.

☒ Life sciences ☐ Behavioural & social sciences ☐ Ecological, evolutionary & environmental sciences

For a reference copy of the document with all sections, see nature.com/documents/nr-reporting-summary-flat.pdf

Life sciences study design

All studies must disclose on these points even when the disclosure is negative.

Sample size	No sample size calculation was performed. All groups were chosen from the available retrospective data as large as possible given the

Sample size	constraint to be matched for age and sex. Since group differences were analyzed using machine learning (ML) approaches for subject level predictions and not classical statistical analysis, no sample size calculation was necessary.
Data exclusions	Datasets were excluded if the image preprocessing pipeline (see methods section) failed and were therefore not processable any further.
Replication	No manual steps are involved in the complete pipeline and results can be reproduced 100%, given the same system setup.
Randomization	All experimental groups were matched for age and sex to avoid bias. No randomization was performed.
Blinding	Blinding was not relevant for this study. All data was processed by the same data pipeline.

Reporting for specific materials, systems and methods

We require information from authors about some types of materials, experimental systems and methods used in many studies. Here, indicate whether each material, system or method listed is relevant to your study. If you are not sure if a list item applies to your research, read the appropriate section before selecting a response.

Materials & experimental systems

n/a	Involved in the study	
☒	☐	Antibodies
☒	☐	Eukaryotic cell lines
☒	☐	Palaeontology and archaeology
☒	☐	Animals and other organisms
☒	☐	Clinical data
☒	☐	Dual use research of concern
☒	☐	Plants

Methods

n/a	Involved in the study	
☒	☐	ChIP-seq
☒	☐	Flow cytometry
☒	☐	MRI-based neuroimaging
